# Towards Asymmetrical Methylene Blue Analogues: Synthesis and Reactivity of 3-*N*′-Arylaminophenothiazines

**DOI:** 10.3390/molecules27093024

**Published:** 2022-05-08

**Authors:** Alena Khadieva, Mansur Rayanov, Ksenia Shibaeva, Alexandr Piskunov, Pavel Padnya, Ivan Stoikov

**Affiliations:** 1 A.M. Butlerov’ Chemistry Institute, Kazan Federal University, 18 Kremlevskaya Street, 420008 Kazan, Russia; as-alex93@mail.ru (A.K.); mrrayanov@stud.kpfu.ru (M.R.); alleoks@mail.ru (K.S.); 2 G.A. Razuvaev Institute of Organometallic Chemistry, Russian Academy of Sciences, 49 Tropinin Street, 603137 Nizhny Novgorod, Russia; pial@iomc.ras.ru

**Keywords:** Methylene Blue, phenothiazine, synthesis, reactivity, DFT

## Abstract

The search for new ways to obtain analogues of the well-known Methylene Blue dye is an important synthetic task. Herein, we proposed and developed an approach to the synthesis of 3-*N*′-arylaminophenothiazines and asymmetrical 3,7-di(*N*′-arylamino)phenothiazines. This approach included the optimization of synthetic strategy by quantification analysis of the positive charge distribution in the cation of 3-*N*′-arylaminophenothiazine derivative. The obtained experimental data are confirmed by DFT studies. Two synthetic routes for asymmetrical phenothiazine diarylamino derivatives were suggested and verified. The developed convenient and versatile synthetic approach makes it easy to obtain aromatic Methylene Blue isostructural analogues with various substituents. As a result, a series of novel 3-*N*′-arylaminophenothiazines and asymmetrical 3,7-di(*N*′-arylamino)phenothiazines containing ester, *tert*-butoxycarbonyl, sulfonic acid, hydroxyl and amine groups were obtained in high yields.

## 1. Introduction

Phenothiazines are a class of heterocyclic compounds, bright representatives of which are Methylene Blue (MB) and its derivatives. Phenothiazine dyes are promising candidates for therapeutic agents against local bacterial infections [1,2], tuberculosis [3], trypanosomiasis [4], malaria [5], yeast infections [6,7], and cancer [8,9,10]. Despite many years of research on phenothiazine derivatives, the search for new ways of their functionalization is still an urgent task for organic chemists [11,12,13,14,15,16,17,18,19,20]. Most examples of phenothiazine modification in the literature are 3,7-substituted phenothiazine derivatives. This is explained by the fact that the 3 and 7 positions of phenothiazine are the most reactive [21], due to the electron-donating effect of the nitrogen atom in the 10 position. A wide series of phenothiazine derivatives with unique physical and physicochemical characteristics (redox activity, conjugation with the nitrogen atom, extended charge delocalization, formation of stable cationic radicals, and dications) can be obtained via the formation of new C–C and N–C bonds in the 3 and 7 positions. 

**Scheme 1:**
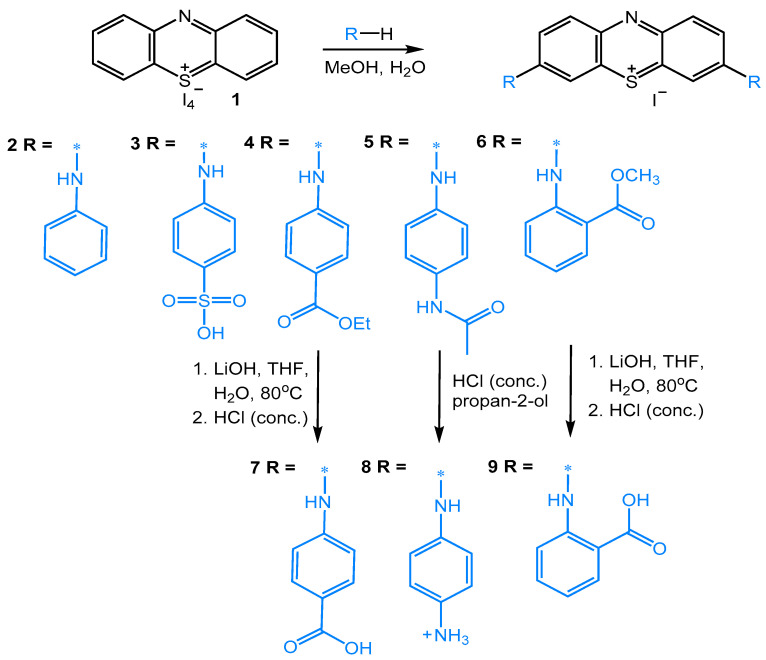
The synthesis of 3,7-bis(*N*′-arylamino)phenothiazines **2–9** [23,24,25,26].

There are some examples of the synthesis of symmetrical 3,7-bis(*N*′-arylamino)phenothiazines containing identical aromatic substituents in the 3 and 7 positions [22]. Synthetic protocols for 3,7-bis(*N*′-arylamino)phenothiazines **2**–**9** with ester, carboxylic acid, sulfonic acid, amide, and amine groups were previously developed in our scientific group (Scheme 1) [23,24,25,26]. Supramolecular colorimetric [27] and electrochemical sensors [28] based on synthesized 3,7-bis(*N*′-arylamino)phenothiazines’ derivatives have been developed. Earlier it was shown that the introduction of aniline derivatives into the 3 and 7 positions of phenothiazine increases the intensity of absorption in the near infrared region [29]. The results obtained are relevant for the design of solar cell materials, as well as for medicine, since the near infrared radiation has a high penetrating power in tissues [30]. The unique electrochemical behavior of these derivatives, and the possibility of “tuning” intermolecular interactions and interactions with biologically important objects, were also demonstrated by the functionalization of the aromatic fragments [24,25]. 

However, many biological and optical applications, as well as the precise supramolecular tuning of non-covalent interactions, require the development of the design of asymmetric disubstituted arylaminophenothiazines as aromatic MB isostructural analogues. Such a synthetic task has not been completely solved and is relevant, since its solution may open prospects for the development of new materials and drugs. This study is devoted to the synthesis of a series of 3-*N*′-arylamino derivatives of phenothiazine, the investigation of their reactivity, and the development of a universal synthetic approach to obtain asymmetric 3,7-di(*N*′-arylamino)phenothiazines.

## 2. Results and Discussion

The stated synthetic problem can be divided into several stages. Initially, the development of a route is planned for the synthesis of monosubstituted in the 3 position phenothiazine derivatives containing fragments of substituted anilines. The next step is the study of the reactivity of the obtained 3-*N*′-arylaminophenothiazines with aniline and its derivatives, in order to optimize the synthesis conditions and develop a versatile synthetic route to obtain differently substituted phenothiazine derivatives as MB isostructural analogues. We also plan to find the optimal methodology for obtaining these compounds by counter syntheses. The use of quantum chemical calculations allowed us to confirm our assumptions about the reactivity of 3-*N*′-arylaminophenothiazines.

### 2.1. Synthesis of 3-N′-Arylaminophenothiazines

The first stage of this work was the development of a synthetic procedure of 3-*N*′-arylaminophenothiazines as precursors for obtaining diarylamino derivatives of phenothiazines, containing different substituents in the 3 and 7 positions. It should be noted that only a few examples of 3-*N*′-arylamino derivatives of phenothiazine are presented in the literature [31], and the structural diversity of 3-amino derivatives of phenothiazine is limited to individual examples of 3-*N*′-alkylamino derivatives [32,33,34,35,36,37,38,39,40]. The synthesis of the 3-substituted compounds remains poorly understood, probably due to the close reactivity of the starting phenothiazin-5-ium tetraiodide **1** and 3-substituted phenothiazin-5-ium in reactions with amines, which leads to low yields of 3-amino derivatives of phenothiazin-5-ium. 

**Scheme 2:**
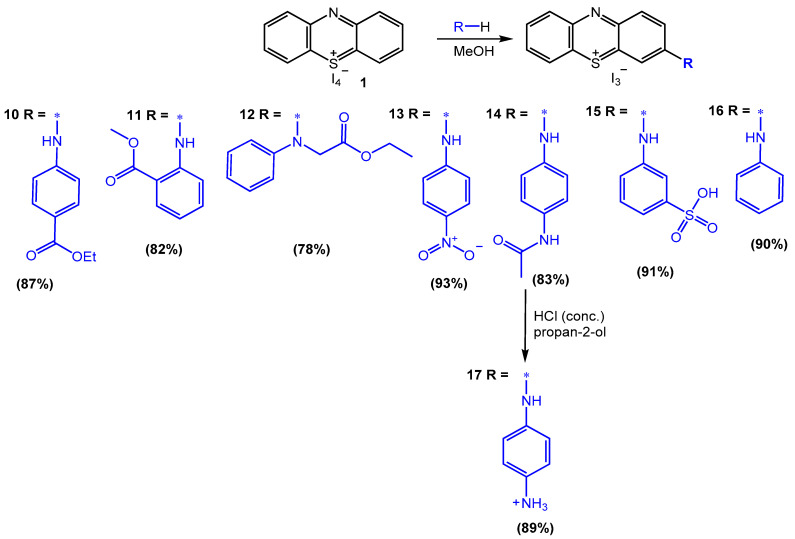
The synthesis of 3-*N*′-arylaminophenothiazines **10**–**17**.

At first, the interaction of the phenothiazin-5-ium tetraiodide **1** with a series of aniline derivatives was studied, to determine the optimal synthetic conditions (Scheme 2). The solvent (methanol or water) was chosen according to the conditions of homogeneous reaction. Methanol was used as a solvent to obtain compounds **10**–**14** and **16**, by analogy with the literature data for 3-*N*′-alkylaminophenothiazine synthesis [32,33,34,35,36,37,38,39,40]. Water was used as a solvent in the synthesis of the compound **15**. Varying the ratios of the compound **1** and aniline derivatives revealed that the most efficient ratio is 1:1. An increase in the amount of arylamine leads to the formation of by-products, namely 3,7-bis(*N*′-arylamino)phenothiazines. The temperature effect on the yield of target compounds was also studied in the range from 0 to 60 °C. A mixture of the compound **1**, the target 3-arylamino derivative, and the by-product 3,7-bis(*N*′-arylamino)phenothiazine derivative was already formed at room temperature. Thus, the optimal conditions for synthesis of 3-*N*′-arylaminophenothiazines were a reaction mixture temperature of 0 °C and the slow dropping of the arylamine to a suspension of phenothiazin-5-ium tetraiodide **1**. When one fragment of an aniline derivative was added to the compound **1** under these conditions, the monosubstituted product precipitated. However, the amount of the obtained precipitate was small (yield 15–63%), so further optimization was carried out to isolate the target 3-substituted products **10**–**16**. The solvent was evaporated off, and the residue was reprecipitated three times from a mixture of methanol-diethyl ether (1:9) at 0 °C. Thus, it was possible to achieve yields of 78–93% for the compounds **10**–**16**. Therefore, a route was developed to obtain 3-substituted phenothiazine derivatives containing fragments of aniline and its derivatives. It consists in the use of polar solvents (methanol, water), low temperatures (0 °C), the ratio of the starting compound **1** to the aniline derivative as 1:1, and isolation and purification by reprecipitation from a mixture of methanol-diethyl ether (1:9). 

As mentioned before, most phenothiazine derivatives are lipophilic. However, phenothiazines with high solubility in water and polar solvents are helpful for many tasks of supramolecular chemistry and materials science. So, the next stage of the work was the hydrolysis of the compound **14** with an acetanilide fragment to obtain a 3-substituted phenothiazine containing one primary amine group in its structure. Previously, the experimental conditions for successful hydrolysis for 3,7-bis(*N*′-arylamino) derivatives of phenothiazine were developed in our scientific group [25]. Therefore, the hydrolysis of the compound **14** was carried out in propan-2-ol in the presence of concentrated hydrochloric acid at the solvent boiling point. The compound **17** was obtained as hydrochloride in 89% yield (Scheme 2).

The structure and the composition of the obtained compounds **10**–**17** were confirmed by ^1^H, ^13^C NMR, IR spectroscopy, mass spectrometry, and elemental analysis (Figures S1–S68). HR ESI mass spectra of the compounds **10**–**17** have shown the presence of a single signal corresponding to the molecular ion peak of the target compounds (Figures S53–S60).

The unambiguous identification of structures by ^1^H NMR spectroscopy of compounds containing triiodide anions can be difficult due to the formation of polyiodides: an exchange process such as [I]^−^ + I_2_ = [I_3_]^−^ can occur in a deuterated solvent [41,42]. Therefore, the characterization of the structures of these compounds by ^1^H NMR spectroscopy was carried out in comparison with the ^1^H NMR spectra of the leuco forms of these compounds. As shown in the literature, in order to characterize the structures of phenothiazine derivatives containing iodide anion, the anion is replaced by another one, most often the chloride anion [35]. However, this approach is not applicable here due to the low solubility of 3-*N*′-arylamino derivatives of phenothiazine in water and alcohols. So, ^1^H NMR spectra of the leuco forms of the compounds **10**–**17** were recorded in a deuterated solvent, with the addition of hydrazine hydrate as a reducing agent (reduction was carried out in- -situ).

**Figure 1:**
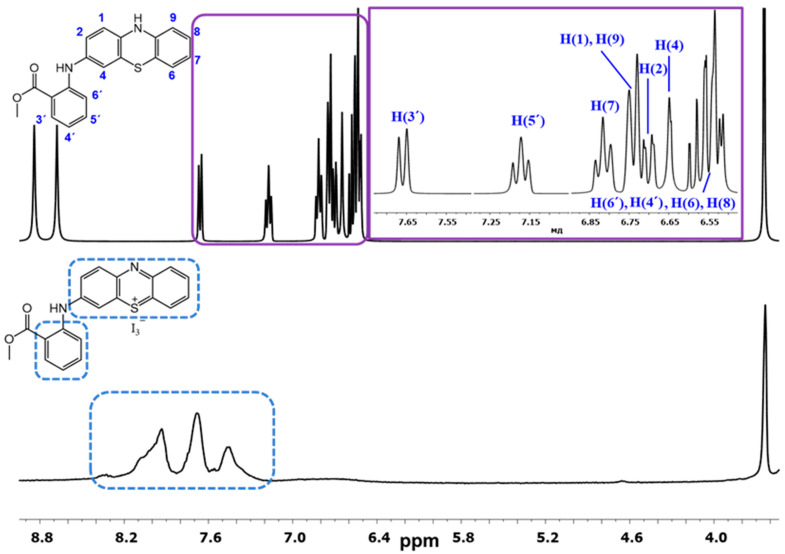
^1^H NMR spectra of the compound **11** (**bottom**) (DMSO-*d*_6_, 25 °C, 400.0 MHz) and its leuco form (**top**) (DMSO-*d*_6_ + 2% N_2_H_4_·H_2_O, 25 °C, 400.0 MHz).

The ^1^H NMR spectrum of the compound **11** shows characteristic signal of methoxy fragment as a singlet, with a chemical shift of 3.80 ppm (Figure 1). The signals of aromatic protons in the form of broadened multiplets are in the region of 7.44–8.32 ppm. In the ^1^H NMR spectrum of the leuco form of the compound **11**, the aromatic proton signals are upshifted (6.50–7.70 ppm) and have the best resolution, which makes it possible to unambiguously identify the structure of the product.

### 2.2. Synthesis of 3,7-di(N′-Arylamino)phenothiazines Containing Different Substituents in the 3 and 7 Positions

**Scheme 3:**
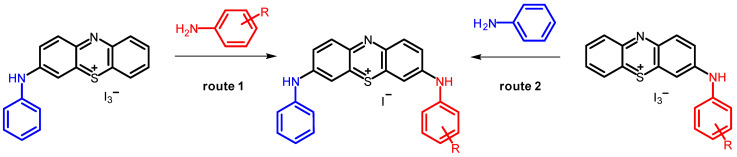
Synthetic routes for phenothiazine diarylamino derivatives.

The next stage of this work was the study of the reactivity of 3-*N*′-arylamino derivatives of phenothiazine in reactions with aromatic amines, to determine the optimal synthetic route for diarylamino derivatives of phenothiazine containing different substituents in the 3 and 7 positions. Synthetic routes for asymmetrical phenothiazine diarylamino derivatives can be divided into two main groups: (Route 1) reactions of the 3-(phenylamino)phenothiazin-5-ium triiodide **16** with a series of arylamines; and (Route 2) reactions of the 3-substituted derivatives **10**–**12** and **14** with aniline (Scheme 3). The reactions were carried out in a mixture of methylene chloride/methanol (*v*/*v* = 1:1), similar to the approaches to the synthesis of phenothiazin-5-ium derivatives described in the literature [33].

**Scheme 4:**
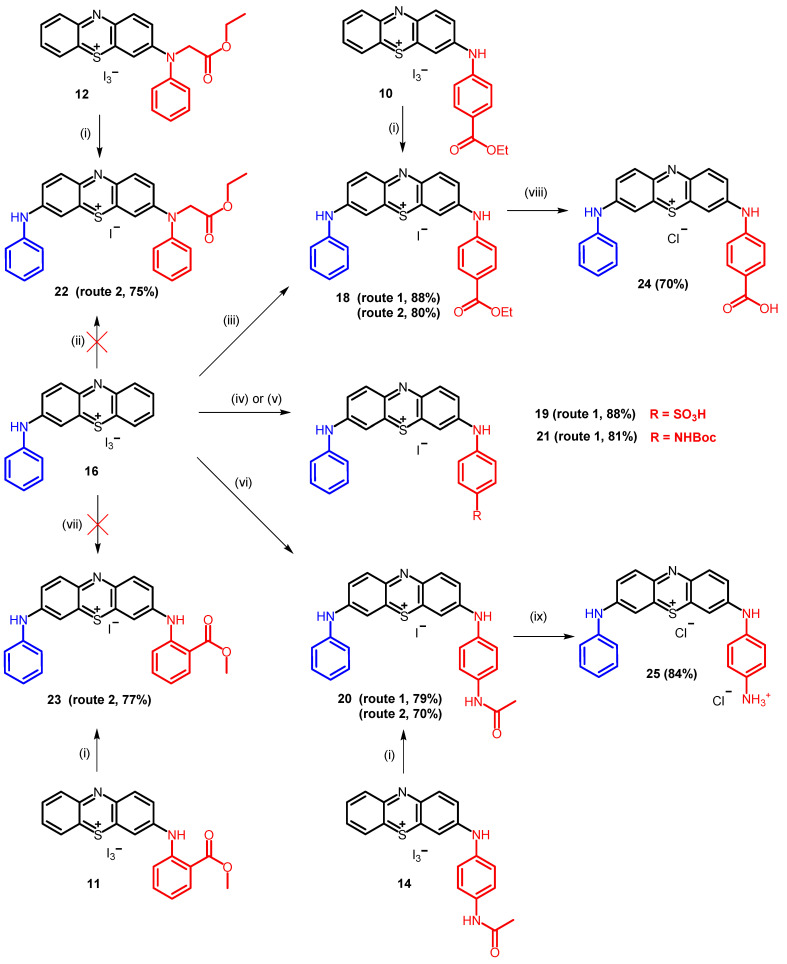
The synthesis of 3,7-di(*N*′-arylamino)phenothiazines **18**–**25**. Reagents: (i) aniline; (ii) *N*-phenylglycine ethyl ester; (iii) ethyl-4-aminobenzoate; (iv) sodium 4-aminobenzenesulfate; (v) *tert*-butyl-(4-aminophenyl)carbamate; (vi) 4-aminoacetanilide; (vii) methyl-2-aminobenzoate; (viii) (a) LiOH, THF/H_2_O, 80 °C, (b) HCl (conc.); (ix) HCl (conc.), propan-2ol.

Due to the two synthetic routes to obtain the diarylamino derivatives of phenothiazine, it is reasonable to carry out “counter” syntheses in order to determine the optimal strategy for obtaining the compounds **18**, **20**, **22**, **23** (Scheme 4). It was shown that the compounds **22** and **23** were obtained only by Route 2, i.e., the reaction of the compounds **11** and **12** with aniline. In the reaction of methyl-2-aminobenzoate or *N*-phenylglycine ethyl ester with the compound **16**, the replacement of the solvent with methylene chloride, a mixture of methylene chloride and methanol, and an increase in temperature also did not lead to the formation of the target products **22** and **23**. The electron-withdrawing effect of the ester group in methyl-2-aminobenzoate and ethyl-4-aminobenzoate was observed. Therefore, the low reactivity of methyl-2-aminobenzoate can be explained by the steric effect of the closely located ester fragment. *N*-Phenylglycine ethyl ester is a reagent containing a secondary amino group, and the low reactivity of N-phenylglycine is due to steric hindrance and an electric inductive effect of ester group. 

The initial procedure for isolating the compounds (washing the precipitate with methanol) led to low yields (37–69%). The main loss was in partial solubility of the target compounds in methanol. To increase the yield of the target diarylamino derivatives of phenothiazine, a procedure was used that is similar to compounds **10**–**16**, namely, three-fold reprecipitation from a mixture of methanol-diethyl ether (1:9) at room temperature. This procedure helped to increase the yields of target compounds **18**–**23** to 70–88%. It should be noted that Route 1 was characterized by higher yields than Route 2.

Hydrolysis reactions of the compounds **18** and **20** have been studied to obtain asymmetric carboxyl and amine phenothiazine derivatives. The compound **18** was hydrolyzed in a THF-water mixture in the presence of lithium hydroxide at 80 °C, followed by treatment with concentrated hydrochloric acid to remove base residues and convert the compound into a salt form. The compound **24** was obtained in 70% yield (Scheme 4). There are no signals of ethoxy protons in the ^1^H NMR spectrum of the compound **24** (Figure S18). The compound **20** was hydrolyzed in propan-2-ol in the presence of concentrated hydrochloric acid at the solvent boiling point. The compound **25** was obtained in 84% yield as hydrochloride (Scheme 4).

**Figure 2:**
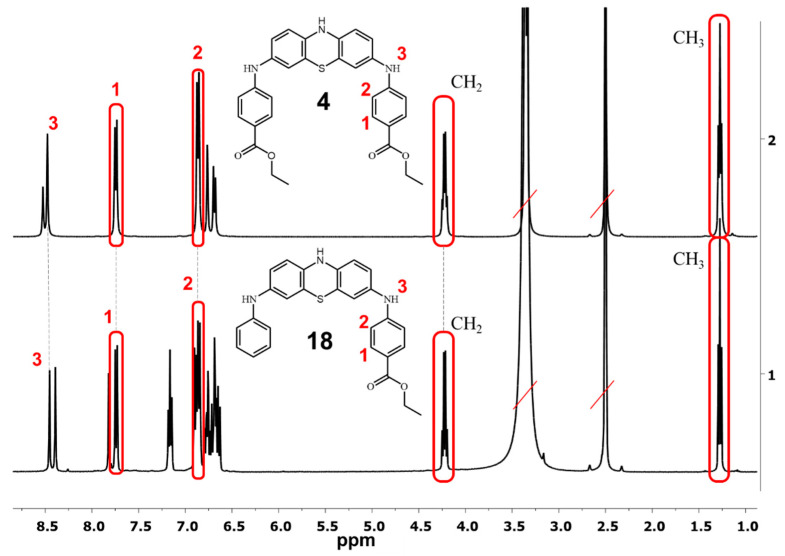
^1^H NMR spectra of leuco forms of the compounds **18** (**bottom**) and **4** (**top**) (DMSO-*d*_6_ + 2% N_2_H_4_·H_2_O, 25 °C, 400.0 MHz).

It should be noted that the chemical shifts of the signals of protons and carbons of the obtained derivatives are close to those of 3,7-bis(N′-arylamino) derivatives of phenothiazine **2**–**9** [23,24,25,26]. Thus, chemical shifts and spin–spin interaction constants of the proton signals of the benzocaine fragment are close in the ^1^H NMR spectra of the leuco forms of the compound **18** and the compound **4** (Figure 2).

**Figure 3:**
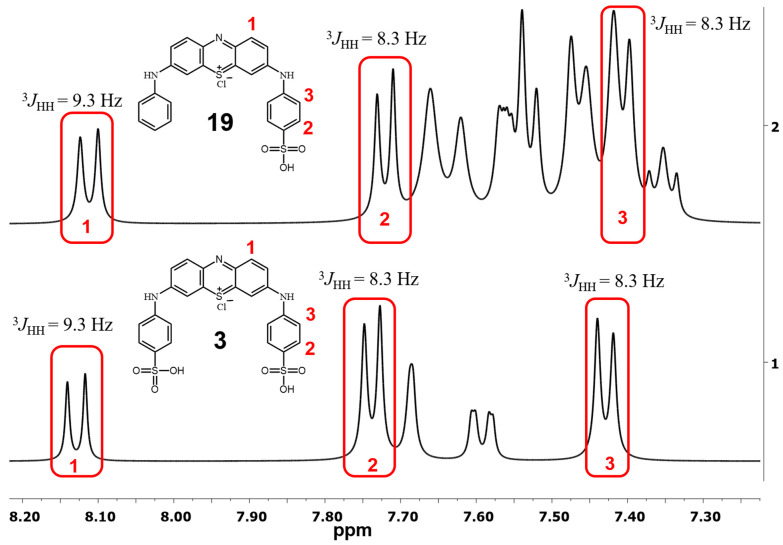
^1^H NMR spectra of the compounds **3** (**bottom**) and **19** (**top**) (DMSO-*d*_6_, 25 °C, 400.0 MHz).

When comparing the ^1^H NMR spectra of salt forms of the compound **19** and the compound **3** [24], the similarity of chemical shifts and the spin–spin coupling constant of the proton signals of the sulfanilic acid fragment is also observed, as well as the phenothiazin-5-ium fragment (Figure 3).

Summarizing, phenothiazin-5-ium and aromatic substituent fragments in the 3 and 7 positions in the ^1^H NMR spectra of the compounds’ **10**–**15** salt and leuco forms can be easily identified, due to the similarity of their structures with the previously obtained 3,7-bis(*N*′-arylamino)phenothiazine derivatives **2**–**9**.

Thus, two synthetic routes for asymmetrical phenothiazine diarylamino derivatives were suggested and verified, i.e., (Route 1) reactions of the 3-(phenylamino)phenothiazin-5-ium triiodide **16** with a series of arylamines, and (Route 2) reactions of the 3-derivatives **10**–**12** and **14** with aniline (Scheme 3). The developed, convenient and versatile synthetic approach makes it easy to obtain aromatic MB isostructural analogues with various substituents. Although synthetic Route 1 was characterized by higher yields, it had limitations. It should be noted that the target compounds **22** and **23** cannot be obtained by synthetic Route 1 when using sterically loaded arylamines (methyl-2-aminobenzoate or *N*-phenylglycine ethyl ester). However, this can be associated not only with steric effects, but also with the reactivity of the 3-(phenylamino)phenothiazin-5-ium triiodide **16**. 

### 2.3. Quantum-Mechanical Calculations

**Figure 4:**
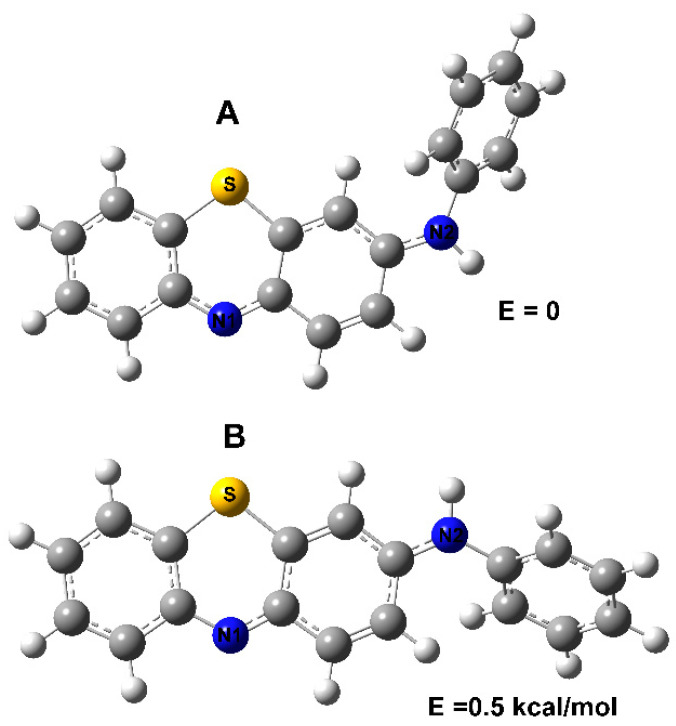
Conformers A and B for the cation of the compound **16** (B3LYP/6-311++G(d,p) DFT calculations).

The next stage of this work was the use of quantum chemical methods (DFT and Hirshfeld charge analysis) to explain the reactivity of phenothiazine derivatives. Geometry optimization for cations of the compounds **1** and **10**–**16** by DFT calculations at the B3LYP/6-311++G(d,p) level of theory found two minima on the potential energy surface respective to conformers A and B for all of the compounds under consideration (Figure 4). The conformation A was slightly advantageous for all cations of the compounds **1** and **10**–**16**, and the discussions are given for this conformation.

**Figure 5:**
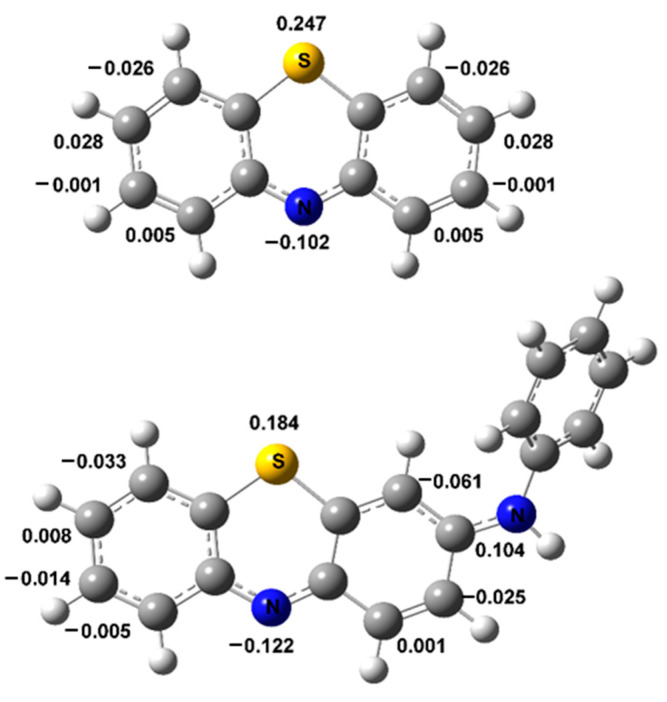
Hirshfeld charges in cations of the compounds **1** (top) and **16** (bottom), according to DFT calculations (B3LYP/6-311++G(d,p)).

The reactions studied in this work are the interaction of phenothiazine tetraiodide with nucleophilic agents. Therefore, one can estimate the electron density distribution by calculating the values of the Hirshfeld positive charges [43] of the atoms in the molecule to assess the reactivity. The more significant positive charge on the carbon atom will promote the nucleophilic attack. The calculated charges of atoms in the unsubstituted phenothiazine cation are presented in Figure 5. The positive charge prevails on the sulfur atom and on the carbons corresponding to the 3 and 7 positions. The data obtained are consistent with the literature [44], as well as with experimental data.

The positive charge is redistributed throughout the molecule when an arylamine substituent is introduced into the 3 position of phenothiazine. The charges were calculated for the compounds **10**–**16** to study the effect of substituents (Table 1). The charge distribution in the phenothiazine fragment of the compound **16** cation is also shown in Figure 5. It should be noted that the carbon at the 7 position has the most positive charge among hydrogen-bonded carbons. It keeps availability for nucleophilic attack when the next *N*-aryl substituent is introduced.

It may be concluded based on calculated values that the positive charge on the seventh carbon atom of the phenothiazine-5 molecule increases in a row of substituents from a positive mesomeric effect to a negative one, i.e., methyl-2-aminobenzoate < 4-aminoacetanilide < ethyl-4-aminobenzoate < 3-aminobenzenesulfonic acid < 4-nitroaniline.

Monosubstituted phenothiazine derivatives **10**–**15** are structural analogues of the compound **16**, so the structure of this compound is discussed as an example. The sum of angles around the nitrogen atom N2 is 359.97°, and the atom has a planar trigonal environment that promotes the conjugation of the phenothiazinium cation aromatic system with a nitrogen lone pair. Nitrogen N2 deviates from the plane of the phenothiazine fragment by only 0.007 Å. The angle between the planes of the phenothiazine fragment and the aniline plane is 56.74°. It indicates the presence of a partial conjugation throughout the phenothiazine fragment and the arylamine substituent.

**Figure 6:**
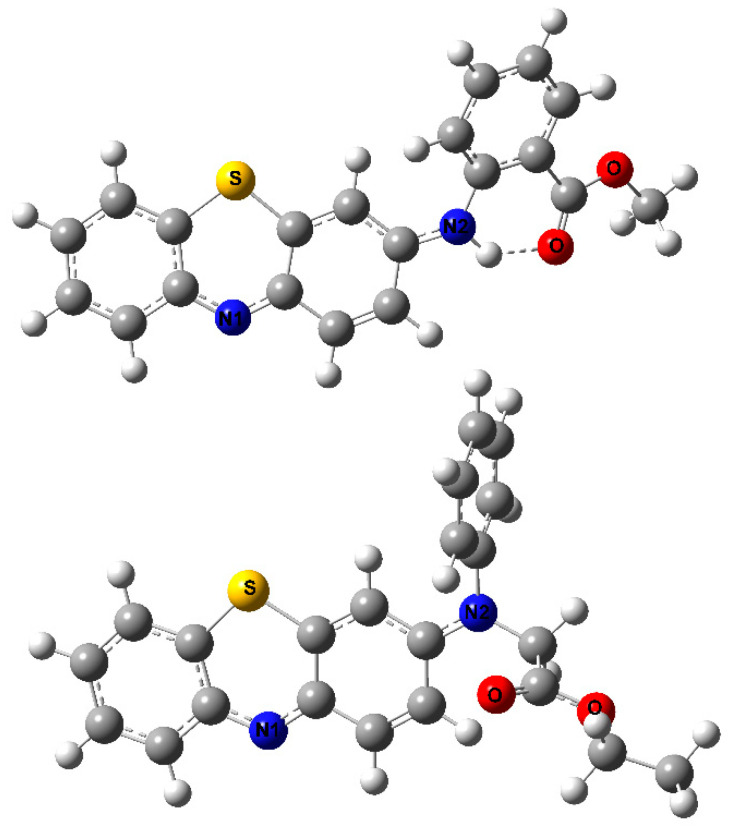
View of the optimized structure of cations of the compounds **11** (**top**) and **12** (**bottom**) (B3LYP/6-311++G(d,p) DFT calculations).

The cation of the compound **11** is characterized by the formation of an intramolecular hydrogen bond between the ester oxygen atom and the NH fragment. It leads to an additional spatial orientation of the arylamine fragment. Consequently, a smaller angle between the planes of aromatic rings up to 38.40° contributed to more efficient conjugation (Figure 6). Nitrogen atom N2 is tertiary in the compound **12**. It can be concluded that there is minimal conjugation between aromatic systems due to the complete release of the arylamine fragment from the plane of the phenothiazine system (angle is 86.19°). The absence of such conjugation leads to a minimal delocalization of the positive charge into the substituent fragment.

**Figure 7:**
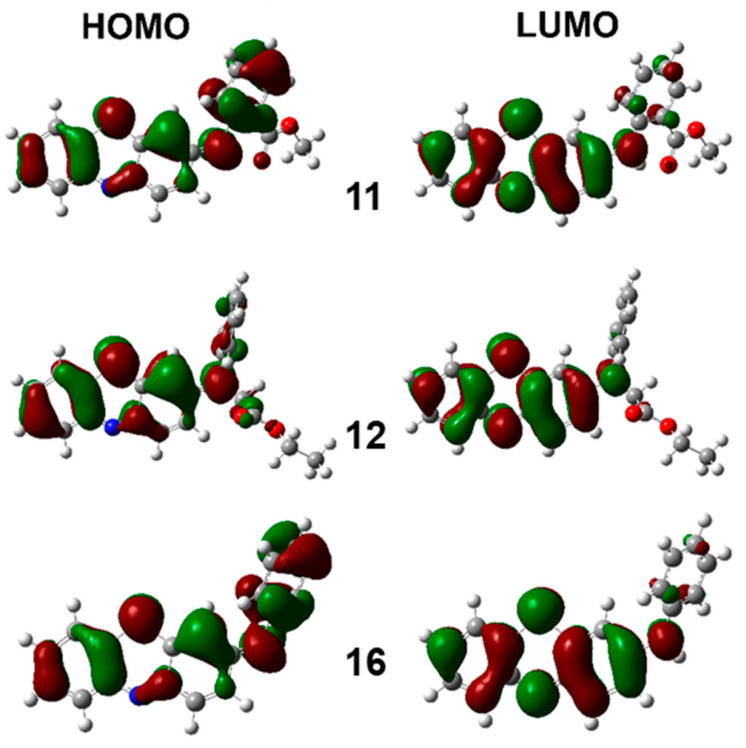
Frontier orbitals for cations of the compounds **11** (**top**), **12** (**middle**), and **16** (**bottom**).

The presence of a delocalized π-system is confirmed by analyzing the shape of the frontier orbitals of cations of the compounds **1** and **10**–**16** (Figure 7). The HOMO orbital is delocalized throughout the phenothiazinium molecule, including the *N*-aryl substituent. The delocalization of the LUMO orbital responsible for the positive charge in the cation into the aniline part also occurs. The exception is the compound **12**. Both frontier orbitals in the cation of the compound **12** do not appreciably delocalize into the *N*-aryl fragment. Thus, substituents in the arylamine fragment at the 3 position of phenothiazine (donor or acceptor groups) can affect the further reactivity at the 7 position, due to the redistribution of the electron density of the heterocyclic system.

Quantum-mechanical calculations are consistent with experimental data, i.e., further reactivity of monosubstitution products of substituted anilines with phenothiazin-5-ium tetraiodide can be predicted by evaluating the positive charge at the seventh carbon atom of the phenothiazin-5-ium fragment. Thus, it can be concluded that the substituents in the aromatic fragment of the 3-aminoaryl derivative of phenothiazin-5-ium affect its reactivity.

### 2.4. Study of Photophysical Properties

**Figure 8:**
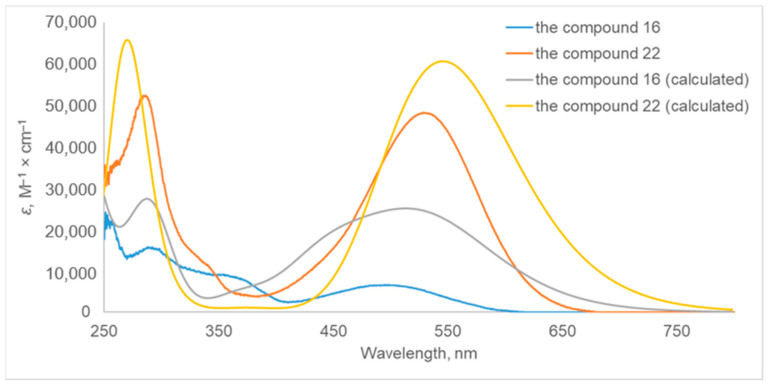
UV-Vis spectra (THF, 1 × 10^−5^ M) and calculated TD-DFT(M06-HF/6-311++G(d,p) UV-Vis spectra of the compounds **16** and **22**.

To study the photophysical properties of the obtained compounds, the UV-Vis spectra of a series of phenothiazines (the compounds **10**, **11**, **12**, **16**, **22**, and **23**) were recorded in THF (Figure 8, Figures S69 and S70). The choice of these compounds was due to their structures, i.e., the compounds **10**, **11**, **12**, and **16** were products of the monosubstitution of the phenothiazine molecule by various aniline derivatives, while the compounds **22** and **23** were their structural disubstituted derivatives. The obtained compounds have a strong absorption in the visible region 450–570 nm with high extinction coefficients (up to ε ≈ 5 × 10^5^ M^–1^ × cm^–1^). It should be noted that the disubstituted derivatives have significantly greater absorption. This absorption determines the deep color inherent in the synthesized compounds. 

The UV-Vis spectra calculated at the M06-HF/6-311++G(d,p) level well reproduce experimental ones. According to the TD-DFT, the orbitals involved in the main low-energy electronic π → π* transitions are HOMO and LUMO for monosubstituted as well as disubstituted phenothiazines. The selected linear response vertical excitation energies and oscillator strengths calculated for **16** and **22** are presented in Table 2.

## 3. Materials and Methods

### 3.1. Instruments and Methods

All reagents and solvents were used directly as purchased, or purified according to the standard procedures. The ^1^H and ^13^C NMR spectra were recorded on a Bruker Avance 400 spectrometer (Bruker Corp., Billerica, MA, USA) (400 MHz for H-atoms) for 3–5% solutions in DMSO-*d*_6_ and DMSO-*d*_6_ with vol. 2% of N_2_H_4_·H_2_O. The residual solvent peaks were used as an internal standard. Elemental analysis was performed on the PerkinElmer 2400 Series II instruments (Perkin Elmer, Waltham, MA, USA). The FTIR ATR spectra were recorded on the Spectrum 400 FT-IR spectrometer (PerkinElmer, Seer Green, Lantrisant, UK) with a Diamond KRS-5 attenuated total internal reflectance attachment (resolution 0.5 cm^−1^, accumulation of 64 scans, recording time 16 s in the wavelength range 400–4000 cm^−1^). High-resolution mass spectra (HRMS) were obtained on a quadrupole time-of-flight (t, qTOF) AB Sciex Triple TOF 5600 mass spectrometer (AB SCIEX PTE. Ltd., Singapore) using a turbo-ion spray source (nebulizer gas nitrogen, a positive ionization polarity, needle voltage 5500 V). Recording of the spectra was performed in “TOF MS” mode with collision energy 10 eV, declustering potentially 100 eV, and with a resolution of more than 30,000 full-width half-maximum. Samples with the analyte concentration of 5 µmol/L were prepared by dissolving the test compounds in the mixture of methanol (HPLC-UV Grade, LabScan, Bangkok, Thailand). Melting points were determined using the Boetius Block apparatus (VEB Kombinat Nagema, Radebeul, Germany).

**Phenothiazin-5-ium tetraiodide (1)** was synthesized by literature [32]. Dp. 170 °C (lit. 170 °C). ^1^H NMR (acetone-*d*_6_, δ, ppm, *J*/Hz): 8.19–8.05 (m, 2H), 8.04–7.82 (m, 2H), 7.81–7.59 (m, 4H).

**3,7-Bis(phenylamino)phenothiazin-5-ium iodide (2)** was synthesized by the previously shown procedure [23]. 

**3,7-Bis((4-sulfophenyl)amino)phenothiazin-5-ium iodide (3)**, **3,7-bis((4-acetamidophenyl)amino)phenothiazin-5-ium iodide (5)(3,7-bis((4-aminophenyl)amino)phenothiazin-5-ium chloride dihydrochloride (8)** were synthesized by the previously shown procedure [24]. 

**3,7-Bis((4-(ethoxycarbonyl)phenyl)amino)phenothiazin-5-ium iodide (4)** and **3,7-bis((4-carboxyphenyl)amino)phenothiazin-5-ium chloride (7)** were synthesized by the previously shown procedure [25].

**3,7-Bis((2-(methoxycarbonyl)phenyl)amino)phenothiazin-5-ium iodide (6)** and **3,7-bis((2-(carboxyl)phenyl)amino)phenothiazin-5-ium chloride (9)** were synthesized by the previously shown procedure [26].

### 3.2. General Procedure for the Synthesis of the Compounds ***10***–***16***

A solution of the corresponding arylamine (0.414 mmol) in 10 mL of methanol or water was added to a suspension of 0.30 g (0.414 mmol) phenothiazin-5-ium tetraiodide (**1**) in 20 mL of methanol (for synthesis of **10**–**14**, **16**) or water (**15**), and the mixture was intensively stirred for 48 h at 0 °C. The solvent was evaporated off, and the residue was reprecipitated three times from a mixture of methanol-diethyl ether (1:9) at 0 °C. 

#### 3.2.1. **3-((4-(Ethoxycarbonyl)phenyl)amino)phenothiazin-5-ium triiodide (10)**

Ethyl-4-aminobenzoate was used as an arylamine. Yield 0.271 g (87%), Mp: 183 °C. ^1^H NMR (DMSO-*d*_6_, δ, ppm, *J*/Hz): 1.35 (t, 3H, ^3^*J*_HH_ = 7.1 Hz, COOCH_2_CH_3_), 4.36 (q, 2H, ^3^*J*_HH_ = 7.0 Hz, COOCH_2_CH_3_), 7.58–7.74 (m, 2H, H(2′), H(6′)), 7.83 (d, 1H, ^3^*J*_HH_ = 8.5 Hz, H(8)), 7.87–8.10 (m, 3H, H(4), H(6), H(2)), 8.15 (d, 2H, ^3^*J*_HH_ = 8.0 Hz, H(3′), H(5′)), 8.18–8.56 (m, 3H, H(1), H(9), H(7)). ^13^C NMR (DMSO-*d*_6_, δ, ppm): 14.7 (CH_3_), 61.2 (CH_2_), 115.6, 116.0, 120.3, 123.9, 129.0, 129.6, 130.2, 131.6, 132.3, 133.2, 136.9, 138.7, 144.0, 148.2, 165.3 (C(O)O). FTIR ATR (ν/cm^−1^): 1717 (C(O)O), 1588 (C-N), 1479 (C = S^+^), 1367, 1119 (C-N). HRMS (ESI): calculated [M–I_3_^−^]^+^ *m*/*z* = 361.1005, found [M–I_3_^−^]^+^ *m*/*z* = 361.1010. El. Anal. found (%): C, 34.07; H, 2.33; I, 51.21; N, 3.82; S, 4.26. C_21_H_17_I_3_N_2_O_2_S. Calculated (%): C, 33.99; H, 2.31; I, 51.30; N, 3.77; S, 4.32.

#### 3.2.2. **3-((2-(Methoxycarbonyl)phenyl)amino)phenothiazin-5-ium triiodide (11)**

Methyl-2-aminobenzoate was used as an arylamine. Yield 0.249 g (82%), Mp: 170 °C. ^1^H NMR (DMSO-*d*_6_, δ, ppm, *J*/Hz): 3.80 (s, 3H, COOCH_3_), 7.44–7.71 (m, 3H, H(4′), H(4), H(6)), 7.72–7.97 (m, 4H, H(5′), H(6′), H(2), H(8)), 8.02–8.32 (m, 4H, H(1), H(9), H(7), H(3′)). ^1^H NMR (DMSO-*d*_6_ + 2% N_2_H_4_·H_2_O, δ, ppm, *J*/Hz): 3.64 (s, 3H, COOCH_3_), 6.50–6.57 (m, 3H, H(4′), H(6), H(8)), 6.59 (dd, 1H, ^3^*J*_HH_ = 7.4 Hz, ^4^*J*_HH_ = 0.9 Hz, H(6′)), 6.65 (d, 1H, ^4^*J*_HH_ = 2.0 Hz, H(4)), 6.70 (dd, 1H, ^3^*J*_HH_ = 8.5 Hz, ^4^*J*_HH_ = 1.9 Hz, H(2)), 6.74 (d, 2H, ^3^*J*_HH_ = 8.1 Hz, H(1), H(9)), 6.82 (t, 1H, ^3^*J*_HH_ = 7.6 Hz, H(7)), 7.17 (t, 1H, ^3^*J*_HH_ = 7.8 Hz, H(5′)), 7.66 (d, 1H, ^3^*J*_HH_ = 8.0 Hz, H(3′)), 8.68 (s, 1H, NH), 8.84 (s, 1H, NH). ^13^C NMR (DMSO-*d*_6_, δ, ppm): 53.1 (CH_3_), 107.7, 126.8, 128.4, 129.9, 131.1, 132.2, 134.6, 136.9, 142.2, 153.3, 165.9 (C(O)O). FTIR ATR (ν/cm^−1^): 1704 (C(O)O), 1579 (C-N), 1479 (C = S^+^), 1399, 1120 (C-N). HRMS (ESI): calculated [M–I_3_^−^]^+^ *m*/*z* = 347.0849, found [M–I_3_^−^]^+^ *m*/*z* = 347.0854. El. Anal. found (%): C, 32.98; H, 2.14; I, 52.32; N, 3.83; S, 4.44. C_20_H_15_I_3_N_2_O_2_S. Calculated (%): C, 32.99; H, 2.08; I, 52.29; N, 3.85; S, 4.40.

#### 3.2.3. **3-((2-Ethoxy-2-oxoethyl)(phenyl)amino)phenothiazin-5-ium triiodide (12)**

*N*-Phenylglycine ethyl ester was used as an arylamine. Yield 0.246 g (78%), Mp: 146 °C. ^1^H NMR (DMSO-*d*_6_, δ, ppm, *J*/Hz): 1.24 (t, 3H, ^3^*J*_HH_ = 7.1 Hz, CH_3_), 4.24 (q, 2H, ^3^*J*_HH_ = 7.1 Hz, COOCH_2_), 5.26 (s, 2H, NCH_2_COO), 7.22–7.42 (m, 1H, H(2)), 7.52 (d, 2H, ^3^*J*_HH_ = 6.6 Hz, H(2′), H(6′)), 7.59–7.66 (m, 1H, H(4′)), 7.69 (t, 2H, ^3^*J*_HH_ = 7.3 Hz, H(3′), H(5′)), 7.90–8.58 (m, 6H, H(1), H(9), H(6), H(4), H(7), H(8)). ^1^H NMR (DMSO-*d*_6_ + 2% N_2_H_4_·H_2_O, δ, ppm, *J*/Hz): 1.17 (t, 3H, ^3^*J*_HH_ = 7.1 Hz, CH_3_), 4.10 (q, 2H, ^3^*J*_HH_ = 7.1 Hz, COOCH_2_), 4.41 (s, 2H, NCH_2_COO), 6.65 (d, 2H, ^3^*J*_HH_ = 8.1 Hz, H(1), H(9)), 6.69 (d, 2H, ^3^*J*_HH_ = 8.1 Hz, H(2′), H(6′)), 6.72–6.79 (m, 3H, H(4), H(4′), H(7)), 6.84 (d, 1H, ^3^*J*_HH_ = 8.5 Hz, H(2)), 6.91 (d, 1H, ^3^*J*_HH_ = 7.6 Hz, H(6)), 7.00 (t, 1H, ^3^*J*_HH_ = 7.6 Hz, H(8)), 7.15 (t, 2H, ^3^*J*_HH_ = 7.8 Hz, H(3′), H(5′)), 8.61 (s, 1H, NH). ^13^C NMR (DMSO-*d*_6_, δ, ppm): 14.7 (CH_3_), 50.1, 61.1, 116.1, 122.0, 123.7, 126.2, 129.0, 129.4, 129.6, 130.2, 131.6, 133.4, 136.8, 143.3, 145.1, 146.7, 169.1 (C(O)O). FTIR ATR (ν/cm^−1^): 1742 (C(O)O), 1585 (C-N), 1459 (C = S^+^), 1370, 1122 (C-N). HRMS (ESI): calculated [M–I_3_^−^]^+^ *m*/*z* = 375.1162, found [M–I_3_^−^]^+^ *m*/*z* = 375.1167. El. Anal. found (%): C, 34.89; H, 2.54; I, 50.43; N, 3.77; S, 4.16. C_22_H_19_I_3_N_2_O_2_S. Calculated (%): C, 34.94; H, 2.53; I, 50.35; N, 3.7; S, 4.24.

#### 3.2.4. **3-((4-Nitrophenyl)amino)phenothiazin-5-ium triiodide (13)**

4-Nitroaniline was used as an arylamine. Yield 0.279 g (93%), Dp: 126 °C. ^1^H NMR (DMSO-*d*_6_ + 2% N_2_H_4_·H_2_O, δ, ppm, *J*/Hz): 6.66–6.79 (m, 3H, H(1), H(2), H(6)), 6.81 (s, 1H, H(4)), 6.84–6.95 (m, 4H, H(9), H(7), H(2′), H(6′)), 7.00 (t, 1H, ^3^*J*_HH_ = 7.0 Hz, H(8)), 8.04 (d, 2H, ^3^*J*_HH_ = 8.5 Hz, H(3′), H(5′)), 8.65 (s, 1H, NH), 9.07 (s, 1H, NH). ^13^C NMR (DMSO-*d*_6_, δ, ppm): 116.3, 123.9, 126.0, 129.0, 129.6, 130.2, 131.6, 133.2, 136.9, 138.7, 141.1, 144.0, 155.2. FTIR ATR (ν/cm^−1^): 1591 (NO_2_), 1542, 1472 (C = S^+^), 1361, 1144 (C-N). HRMS (ESI): calculated [M–I_3_^−^]^+^ *m*/*z* = 334.0645, found [M–I_3_^−^]^+^ *m*/*z* = 334.0650. El. Anal. found (%): C, 30.25; H, 1.63; I, 53.26; N, 5.84; S, 4.49. C_18_H_12_I_3_N_3_O_2_S. Calculated (%): C, 30.23; H, 1.69; I, 53.24; N, 5.88; S, 4.48.

#### 3.2.5. **3-((4-Acetamidophenyl)amino)phenothiazin-5-ium triiodide (14)**

*N*-(4-aminophenyl)acetamide was used as an arylamine. Yield 0.249 g (83%), Mp: 195 °C. ^1^H NMR (DMSO-*d*_6_, δ, ppm, *J*/Hz): 2.10 (s, 3H, CH_3_), 7.55 (d, 2H, ^3^*J*_HH_ = 8.0 Hz, H(2′), H(6′)), 7.72–7.93 (m, 5H, H(5′), H(3′), H(2), H(7), H(8)), 7.96 (s, 1H, H(4)), 8.12–8.23 (m, 2H, H(9), H(6)), 8.27 (d, ^3^*J*_HH_ = 6.6 Hz, 1H, H(1)), 10.31 (s, 1H, NH). ^13^C NMR (DMSO-*d*_6_, δ, ppm): 23.8, 116.0, 120.4, 122.3, 125.9, 127.0, 127.6, 130.2, 131.6, 134.2, 135.9, 138.7, 140.4, 149.0, 169.4. FTIR ATR (ν/cm^−1^): 2923 (NH), 1674 (C = O), 1587 (C-N), 1501 (C-C), 1390, 1120 (C-N). HRMS (ESI): calculated [M–I_3_^−^]^+^ *m*/*z* = 346.1009, found [M–I_3_^−^]^+^ *m*/*z* = 346.1014. El. Anal. found (%): C, 32.94; H, 2.24; I, 52.37; N, 5.79; S, 4.38. C_20_H_16_I_3_N_3_OS. Calculated (%): C, 33.04; H, 2.22; I, 52.36; N, 5.78; S, 4.41.

#### 3.2.6. **3-((3-Sulfophenyl)amino)phenothiazin-5-ium chloride (15)**

The sodium 3-aminobenzene-1-sulfate was used as the arylamine. The resulting precipitate was treated with concentrated hydrochloric acid (20 mL). Yield 0.281 g (91%), Mp: 160 °C. ^1^H NMR (DMSO-*d*_6_, δ, ppm, *J*/Hz): 7.58–7.66 (m, 2H, H(8), H(2)), 7.69–7.78 (m, 2H, H(5′), H(7)), 7.83 (d, 1H, ^3^*J*_HH_ = 8.4 Hz, H(4′)), 7.87–7.99 (m, 3H, H(4), H(6′), H(2′)), 8.21–8.29 (m, 2H, H(9), H(6)), 8.33 (d, 1H, ^3^*J*_HH_ = 6.3 Hz, H(1)), 11.10 (s, 1H, NH). ^13^C NMR (DMSO-*d*_6_, δ, ppm): 116.0, 122.3, 123.9, 124.2, 125.6, 127.6, 129.0, 129.6, 130.2, 131.6, 133.2, 136.9, 138.7, 142.4, 143.9, 144.0. FTIR ATR (ν/cm^−1^): 1558 (C-N), 1359 (C = S^+^), 1230 (C-N), 1131 (SO_3_), 1117 (SO_3_), 1027 (C-S-C), 995, 841, 680 (C-S). HRMS (ESI): calculated [M–Cl^−^]^+^ *m*/*z* = 369.0362, found [M–Cl^−^]^+^ *m*/*z* = 369.0367. El. Anal. found (%): C, 53.39; H, 3.32; Cl, 8.71; N, 6.90; S, 15.76. C_18_H_13_ClN_2_O_3_S_2_ Calculated (%): C, 53.40; H, 3.24; Cl, 8.76; N, 6.92; S, 15.84.

#### 3.2.7. **3-(Phenylamino)phenothiazin-5-ium triiodide (16)**

Aniline was used as an arylamine. Yield 0.249 g (90%), Mp: 160 °C. ^1^H NMR (DMSO-*d*_6_, δ, ppm, *J*/Hz): 7.42–7.58 (m, 3H, H(7), H(8), H(2)), 7.60–7.68 (m, 2H, H(6′), H(2′)), 7.81 (d, 1H, ^3^*J*_HH_ = 9.3 Hz, H(6)), 7.84–7.95 (m, 3H, H(4′), H(3′), H(5′)), 8.06–8.36 (m, 3H, H(1), H(9), H(4)), 11.06 (s, 1H, NH). ^1^H NMR (DMSO-*d*_6_ + 2% N_2_H_4_·H_2_O, δ, ppm, *J*/Hz): 6.60–6.64 (m, 1H, H(9)), 6.64–6.67 (m, 2H, H(2), H(1)), 6.67–6.76 (m, 3H, H(4′), H(6′), H(2′)), 6.83–6.90 (m, 3H, H(4), H(6), H(8)), 7.00 (td, 1H, ^3^*J*_HH_ = 7.9 Hz, ^4^*J*_HH_ = 1.3 Hz, H(7)), 7.13 (t, 2H, ^3^*J*_HH_ = 7.9 Hz, H(3′), H(5′)), 7.98 (s, 1H, NH), 8.65 (s, 1H, NH). ^13^C NMR (DMSO-*d*_6_, δ, ppm): 116.0, 118.9, 118.9, 121.6, 123.9, 129.0, 129.3, 129.6, 130.2, 131.6, 133.2, 136.9, 138.7, 143.2, 144.0. FTIR ATR (ν/cm^−1^): 1586 (C-N), 1486 (C = S^+^), 1375 (C = S^+^), 1121 (C-N). HRMS (ESI): calculated [M–I_3_^−^]^+^ *m*/*z* = 289.0794, found [M–I_3_^−^]^+^ *m*/*z* = 289.0797. El. Anal. found (%): C, 32.23; H, 1.86; I, 56.8; N, 4.27; S, 4.84. C_18_H_13_I_3_N_2_S. Calculated (%): C, 32.26; H, 1.96; I, 56.82; N, 4.18; S, 4.78.

### 3.3. Procedure for the Synthesis of 3-((4-ammoniophenyl)amino)phenothiazin-5-ium chloride ***(17)***

In a round-bottom flask equipped with a magnetic stirrer and a reflux condenser with a calcium chloride tube, 10 mL of concentrated hydrochloric acid solution was added to a solution of the compound **14** (0.218 g, 0.3 mmol) in 10 mL of propan-2-ol. The reaction mixture was refluxed for 40 h, after which the propan-2-ol was evaporated in a rotary evaporator. The precipitate formed was filtered off, washed with aqueous 10% ammonia solution (2 × 15 mL), diethyl ether (2 × 15 mL), concentrated hydrochloric acid solution (2 × 30 mL), water (2 × 30 mL).

#### **3-((4-Ammoniophenyl)amino)phenothiazin-5-ium chloride** **(17)**

Yield 0.101 g (89%), Mp: 175 °C. ^1^H NMR (DMSO-*d*_6_ + 2% N_2_H_4_·H_2_O, δ, ppm, *J*/Hz): 6.41 (d, 1H, ^4^*J*_HH_ = 1.6 Hz, H(4)), 6.47–6.56 (m, 3H, H(1), H(3′), H(5′)), 6.65 (d, 1H, ^3^*J*_HH_ = 8.3 Hz, H(2)), 6.69 (d, 1H, ^3^*J*_HH_ = 7.5 Hz, H(9)), 6.72 (d, 2H, ^3^*J*_HH_ = 8.3 Hz, H(2′), H(6′)), 6.87 (d, 1H, ^3^*J*_HH_ = 7.5 Hz, H(6)), 6.94 (t, 1H, ^3^*J*_HH_ = 7.1 Hz, H(8)), 7.01 (t, 1H, ^3^*J*_HH_ = 7.1 Hz, H(7)), 7.21 (br.s, 2H, NH_2_), 8.32 (s, 1H, NH). ^13^C NMR (DMSO-*d*_6_, δ, ppm): 116.0, 116.1, 120.4, 123.9, 129.0, 129.6, 130.2, 131.6, 133.2, 136.1, 136.9, 138.7, 143.1, 144.0. FTIR ATR (ν/cm^−1^): 3209 (NH_3_^+^), 1587 (C-N), 1487 (C = S^+^), 1370, 1121 (C-N). HRMS (ESI): calculated [M–HCl–Cl^−^]^+^ *m*/*z* = 304.0903, found [M–HCl–Cl^−^]^+^ *m*/*z* = 304.0908. El. Anal. found (%): C, 57.47; H, 4.08; Cl, 18.86; N, 11.15; S, 8.44. C_18_H_14_Cl_3_N_3_S. Calculated (%): C, 57.45; H, 4.02; Cl, 18.84; N, 11.17; S, 8.52.

### 3.4. General Procedure for the Synthesis of the Compounds ***18***–***23***

**Route 1**: A solution of the corresponding amine (0.9 mmol) in 10 mL of methanol or water was added to a suspension of 0.2 g (0.3 mmol) of the compound **16** in 20 mL of a mixture of methanol and methylene chloride (1:1 *v*/*v*), and the mixture was intensively stirred for 48 h at room temperature. The solvent was evaporated off, and the residue was reprecipitated three times from a mixture of methanol-diethyl ether (1:9) at room temperature.

**Route 2**: A solution of aniline 0.083 g (0.9 mmol) in methanol was added to a suspension of 0.3 mmol of 3-substituted phenothiazine derivative (the compounds **10**–**12** and **14**) in 20 mL of methanol, and the mixture was intensively stirred for 48 h at room temperature. The solvent was evaporated off, and the residue was reprecipitated three times from a mixture of methanol-diethyl ether (1:9) at room temperature.

#### 3.4.1. **3-((4-(Ethoxycarbonyl)phenyl)amino)-7-(phenylamino)phenothiazin-5-ium iodide (18)**

Obtained by Route 1. Ethyl-4-aminobenzoate was used as an arylamine. Yield 0.151 g (88%). Obtained by Route 2. Yield 0.137 g (80%). Mp: 197 °C. ^1^H NMR (DMSO-*d*_6_, δ, ppm, *J*/Hz): 1.33 (t, 3H, ^3^*J*_HH_ = 7.1 Hz, COOCH_2_CH_3_), 4.33 (q, 2H, ^3^*J*_HH_ = 7.0 Hz, COOCH_2_CH_3_), 6.62–6.74 (m, 1H, H(8)), 8.12–8.20 (m, 2H, H(3″), H(5″)), 7.09 (t, 1H, ^3^*J*_HH_ = 7.3 Hz, H(4″)), 7.33–7.65 (m, 7H, H(1), H(9), H(2), H(6″), H(2′), H(6′), H(2″)), 7.80 (s, 1H, H(4)), 7.68 (s, 1H, H(6)), 8.06 (d, 2H, ^3^*J*_HH_ = 8.5 Hz, H(5′), H(3′)). ^1^H NMR (DMSO-*d*_6_ + 2% N_2_H_4_·H_2_O, δ, ppm, *J*/Hz): 1.27 (t, 3H, ^3^*J*_HH_ = 7.1 Hz, COOCH_2_CH_3_), 4.22 (q, 2H, ^3^*J*_HH_ = 7.1 Hz, COOCH_2_CH_3_), 6.64 (d, 1H, ^3^*J*_HH_ = 8.5 Hz, H(8)), 6.66–6.74 (m, 3H, H(2), H(6), H(4″)), 6.76 (d, 1H, ^4^*J*_HH_ = 2.1 Hz, H(4)), 6.78 (d, 1H, ^3^*J*_HH_ = 8.5 Hz, H(1)), 6.83–6.87 (m, 3H, H(2′), H(6′), H(9)), 6.89 (d, 2H, ^3^*J*_HH_ = 8.0 Hz, H(2″), H(6″)), 7.16 (t, 2H, ^3^*J*_HH_ = 7.8 Hz, H(3″), H(5″)), 7.74 (d, 2H, ^3^*J*_HH_ = 8.7 Hz, H(3′), H(5′)), 7.84 (s, 1H, NH), 8.41 (s, 1H, NH), 8.47 (s, 1H, NH). ^13^C NMR (DMSO-*d*_6_, δ, ppm): 60.1 (CH_3_), 113.1, 115.0, 115.2, 115.4, 116.8, 117.0, 118.5, 118.9, 119.5, 121.0, 129.4, 131.3, 149.9, 166.0 (C(O)O). FTIR ATR (ν/cm^−1^): 1712 (C(O)O), 1586 (C-N), 1479 (C = S^+^), 1386, 1122 (C-N). HRMS (ESI): calculated [M–I^−^]^+^ *m*/*z* = 452.1427, found [M–I^−^]^+^ *m*/*z* = 452.1431. El. Anal. found (%): C, 55.92; H, 3.88; I, 21.92; N, 7.37; S, 5.44. C_27_H_22_IN_3_O_2_S. Calculated (%): C, 55.97; H, 3.83; I, 21.9; N, 7.25; S, 5.53.

#### 3.4.2. **3-(Phenylamino)-7-((4-sulfophenyl)amino)phenothiazin-5-ium chloride (19)**

Obtained by Route 1. The sodium 4-aminobenzenesulfate was used as the arylamine. The resulting precipitate was treated with concentrated hydrochloric acid (20 mL). Yield 0.137 g (88%), Mp: 160 °C. ^1^H NMR (DMSO-*d*_6_ + 2% N_2_H_4_·H_2_O, δ, ppm, *J*/Hz): 6.60–6.82 (m, 9H, H(2′), H(6′), H(9), H(1), H(4), H(2), H(6), H(4″), H(8)), 6.86 (d, 2H, ^3^*J*_HH_ = 7.9 Hz, H(2″), H(6″)), 7.14 (t, 2H, ^3^*J*_HH_ = 7.7 Hz, H(3″), H(5″)), 7.41 (d, 2H, ^3^*J*_HH_ = 8.5 Hz, H(3′), H(5′)), 8.01 (s, 1H, NH), 8.25 (s, 1H, NH), 8.55 (s, 1H, NH). ^13^C NMR (DMSO-*d*_6_, δ, ppm): 116.0, 118.9, 119.7, 121.6, 123.9, 127.8, 129.3, 133.2, 136.2, 138.7, 140.7, 143.2, 144.0. FTIR ATR (ν/cm^−1^): 1579 (C-N), 1340 (C = S^+^), 1225 (C-N), 1155 (SO_3_), 1117 (SO_3_), 1029 (C-S-C), 1005, 794, 687 (C-S). HRMS (ESI): calculated [M–Cl^−^]^+^ *m*/*z* = 460.0784, found [M–Cl^−^]^+^ *m*/*z* = 460.0789. El. Anal. found (%): C, 58.02; H, 3.73; Cl, 7.23; N, 8.48; S, 12.81. C_24_H_18_ClN_3_O_3_S_2_. Calculated (%): C, 58.12; H, 3.66; Cl, 7.15; N, 8.47; S, 12.93.

#### 3.4.3. **3-((4-Acetamidophenyl)amino)-7-(phenylamino)phenothiazin-5-ium iodide (20)**

Obtained by Route 1. *N*-(4-aminophenyl)acetamide was used as an arylamine. Yield 0.134 g (79%). Obtained by Route 2. Yield 0.118 g (70%). Mp: 206 °C. ^1^H NMR (DMSO-*d*_6_ + 2% N_2_H_4_·H_2_O, δ, ppm, *J*/Hz): 1.98 (s, 3H, CH_3_), 6.58–6.72 (m, 6H, H(9), H(4), H(2), H(6), H(8), H(4″)), 6.75 (dd, 1H, ^3^*J*_HH_ = 8.4 Hz, ^4^*J*_HH_ = 2.4 Hz, H(1)), 6.82–6.89 (m, 4H, H(2″), H(6″), H(2′), H(6′)), 7.14 (t, 2H, ^3^*J*_HH_ = 7.9 Hz, H(3″), H(5″)), 7.35 (d, 2H, ^3^*J*_HH_ = 8.8 Hz, H(3′), H(5′)), 7.78 (s, 1H, NH), 7.88 (s, 1H, NH), 8.33 (s, 1H, NH), 9.91 (s, 1H, NH). ^13^C NMR (DMSO-*d*_6_, δ, ppm): 24.5, 120.4, 123.1, 123.9, 126.8, 129.4, 129.8, 130.3, 132.5, 136.8, 137.5, 138.1, 138.4, 138.8, 151.6, 152.0, 168.9. FTIR ATR (ν/cm^−1^): 3028 (NH), 1669 (C = O), 1595 (C-N), 1509 (C-C), 1372 (C = S^+^), 1134 (C-N). HRMS (ESI): calculated [M–I^−^]^+^ *m*/*z* = 437.1431, found [M–I^−^]^+^ *m*/*z* = 437.1436. El. Anal. found (%): C, 55.28; H, 3.73; I, 22.53; N, 9.85; S, 5.7. C_26_H_21_IN_4_OS. Calculated (%): C, 55.33; H, 3.75; I, 22.48; N, 9.93; S, 5.68.

#### 3.4.4. **3-((4-((*Tert*-butoxycarbonyl)amino)phenyl)amino)-7-(phenylamino)phenothiazin-5-ium iodide (21)**

Obtained by Route 1. *tert*-Butyl-(4-aminophenyl)carbamate was used as an arylamine. Yield 0.152 g (81%), Mp: 196 °C. ^1^H NMR (DMSO-*d*_6_, δ, ppm, *J*/Hz): 1.47 (s, 9H, CH_3_), 7.19–7.25 (m, 3H, H(9), H(6′), H(2′)), 7.31–7.39 (m, 2H, H(8), H(2)), 7.46 (t, 2H, ^3^*J*_HH_ = 8.9 Hz, H(3″), H(5″)), 7.49–7.58 (m, 6H, H(3′), H(5′), H(4), H(6), H(6″), H(2″)), 7.58–7.64 (m, 1H, H(1)), 8.01–8.10 (m, 1H, H(4″)), 9.61 (s, 1H, NHBOC), 10.92 (s, 1H, NH), 11.10 (s, 1H, NH). ^13^C NMR (DMSO-*d*_6_, δ, ppm): 79.8, 119.3, 119.5, 123.2, 123.8, 124.1, 124.2, 125.9, 126.8, 130.3, 131.6, 136.8, 137.6, 138.1, 138.9, 139.7, 153.1. FTIR ATR (ν/cm^−1^): 2980 (CH_3_), 1700 (C(O)O), 1589 (C-N), 1477(C-N), 1407 (CH_3_), 1369, 1127 (C-N). HRMS (ESI): calculated [M–I]^+^ *m*/*z* = 495.1849, found [M–I]^+^ *m*/*z* = 495.1854. El. Anal. found (%): C, 55.87; H, 4.37; I, 20.45; N, 8.98; S, 5.14. C_29_H_27_IN_4_O_2_S. Calculated (%): C, 55.95; H, 4.37; I, 20.39; N, 9.00; S, 5.15.

#### 3.4.5. **3-((2-Ethoxy-2-oxoethyl)(phenyl)amino)-7-(phenylamino)phenothiazin-5-ium iodide (22)**

Obtained by Route 2. Yield 0.133 g (75%), Mp: 187 °C. ^1^H NMR (DMSO-*d*_6_, δ, ppm, *J*/Hz): 1.21 (t, 3H, ^3^*J*_HH_ = 7.0 Hz, CH_3_), 4.19 (q, 2H, ^3^*J*_HH_ = 7.1 Hz, COOCH_2_), 4.98 (s, 2H, NCH_2_COO), 7.16 (d, 1H, ^3^*J*_HH_ = 9.0 Hz, H(8)), 7.33–7.68 (m, 13H, H(9), H(2), H(4), H(6), H(2′), H(6′), H(3′), H(5′), H(2″), H(6″), H(3″), H(5″), H(4″)), 8.05 (d, 1H, ^3^*J*_HH_ = 9.5 Hz, H(1)), 8.09–8.14 (m, 1H, H(4′)). ^13^C NMR (DMSO-*d*_6_, δ, ppm): 55.1, 61.9, 108.0, 120.8, 123.3, 123.6, 127.0, 127.4, 129.2, 130.4, 130.5, 131.1, 138.2, 139.2, 139.6, 168.7 (C(O)O). FTIR ATR (ν/cm^−1^): 1737 (C(O)O), 1581 (C-N), 1478 (C = S^+^), 1378, 1129 (C-N). HRMS (ESI): calculated [M–I^–^]^+^ *m*/*z* = 466.1589, found [M–I^–^]^+^ *m*/*z* = 466.1591. El. Anal. found (%): C, 56.62; H, 4.13; I, 21.28; N, 7.14; S, 5.50. C_28_H_24_IN_3_O_2_S. Calculated (%): C, 56.67; H, 4.08; I, 21.38; N, 7.08; S, 5.40.

#### 3.4.6. **3-((2-(Methoxycarbonyl)phenyl)amino)-7-(phenylamino)phenothiazin-5-ium iodide (23)**

Obtained by Route 2. Yield 0.129 g (77%), Mp: 204 °C. ^1^H NMR (DMSO-*d*_6_, δ, ppm, *J*/Hz): 3.80 (s, 3H, COOCH_3_), 7.37 (t, 1H, ^3^*J*_HH_ = 7.0 Hz, H(4″)), 7.45–7.60 (m, 8H, H(2), H(8), H(6), H(4′), H(2″), H(6″), H(3″), H(5″)), 7.63 (s, 1H, H(4)), 7.68 (d, 1H, ^3^*J*_HH_ = 8.0 Hz, H(6′)), 7.77 (t, 1H, ^3^*J*_HH_ = 7.6 Hz, H(5′)), 8.03 (d, 1H, ^3^*J*_HH_ = 7.8 Hz, H(3′)), 8.13 (d, 2H, ^3^*J*_HH_ = 9.3 Hz, H(1), H(9)), 10.88 (s, 1H, NH), 11.18 (s, 1H, NH). ^13^C NMR (DMSO-*d*_6_, δ, ppm): 52.9 (CH_3_), 107.6, 123.5, 124.8, 126.3, 127.6, 130.4, 132.1, 134.6, 137.8, 139.2, 139.4, 152.3, 166.2 (C(O)O). FTIR ATR (ν/cm^−1^): 1699 (C(O)O), 1584 (C-N), 1461 (C = S^+^), 1384, 1131 (C-N). HRMS (ESI): calculated [M–I]^+^ *m*/*z* = 438.1271, found [M–I]^+^ *m*/*z* = 438.1276. El. Anal. found (%): C, 55.13; H, 3.62; I, 22.37; N, 7.64; S, 5.61. C_26_H_20_IN_3_O_2_S. Calculated (%): C, 55.23; H, 3.57; I, 22.44; N, 7.43; S, 5.67.

### 3.5. Procedure for the Synthesis of 3-((4-carboxyphenyl)amino)-7-(phenylamino)phenothiazin-5-ium chloride ***(24)***

In a round-bottom flask equipped with a magnetic stirrer and a reflux condenser with a calcium chloride tube, 0.126 g (3 mmol) of lithium hydroxide monohydrate was added to a solution of 0.173 g (0.3 mmol) of the compound **18** in 10 mL of THF and 2 mL of water. The reaction mixture was heated for 48 h, after which THF was evaporated on a rotary evaporator. The precipitate that formed was treated with concentrated hydrochloric acid, then was filtered off, washed with diethyl ether (2 × 15 mL), 2M hydrochloric acid (2 × 30 mL).

#### **3-((4-Carboxyphenyl)amino)-7-(phenylamino)phenothiazin-5-ium chloride** **(24)**

Yield 0.096 g (70%), Mp: 227 °C. ^1^H NMR (DMSO-*d*_6_, δ, ppm, *J*/Hz): 7.17–7.67 (m, 7H, H(2), H(6″), H(2′), H(6′), H(2″), H(4″), H(8)), 7.89–7.68 (m, 4H, H(4), H(6), H(1), H(9)), 7.96–8.21 (m, 4H, H(3″), H(5″), H(5′), H(3′)), 11.65 (s, 1H, NH), 12.04 (s, 1H, NH). ^1^H NMR (DMSO-*d*_6_ + 2% N_2_H_4_·H_2_O, δ, ppm, *J*/Hz): 6.59–6.81 (m, 9H, H(1), H(4), H(2′), H(6′), H(9), H(2), H(6), H(4″), H(9)), 6.86 (d, 2H, ^3^*J*_HH_ = 7.5 Hz, H(2″), H(6″)), 7.13 (t, 2H, ^3^*J*_HH_ = 7.1 Hz, H(3″), H(5″)), 7.64 (d, 2H, ^3^*J*_HH_ = 7.3 Hz, H(3′), H(5′)), 7.97 (s, 1H, NH), 8.15 (s, 1H, NH), 8.50 (s, 1H, NH). ^13^C NMR (DMSO-*d*_6_, δ, ppm): 121.7, 123.5, 127.6, 129.9, 130.4, 131.2, 131.5, 135.0, 137.1, 137.7, 138.4, 138.7, 142.6, 152.6, 167.0 (C(O)O). FTIR ATR (ν/cm^−1^): 1687 (C(O)O), 1579 (C-N), 1509 (C-C), 1372, 1125 (C-N). HRMS (ESI): calculated [M–Cl^−^]^+^ *m*/*z* = 424.1114, found [M–Cl^−^]^+^ *m*/*z* = 424.1119. El. Anal. found (%): C, 65.25; H, 4.02; Cl, 7.69; N, 9.13; S, 7.01 C_25_H_18_ClN_3_O_2_S. Calculated (%): C, 65.28; H, 3.94; Cl, 7.71; N, 9.14; S, 6.97.

### 3.6. Procedure for the Synthesis of 3-((4-ammoniophenyl)amino)-7-(phenylamino)phenothiazin-5-ium chloride ***(25)***

In a round-bottom flask equipped with a magnetic stirrer and a reflux condenser with a calcium chloride tube, 10 mL of concentrated hydrochloric acid solution was added to a solution of the compound **20** (0.169 g, 0.3 mmol) in 10 mL of propan-2-ol. The reaction mixture was refluxed for 40 h, after which the propan-2-ol was evaporated on a rotary evaporator. The precipitate formed was filtered off, washed with aqueous 10% ammonia solution (2 × 15 mL), diethyl ether (2 × 15 mL), concentrated hydrochloric acid solution (2 × 30 mL), water (2 × 30 mL).

#### **3-((4-Ammoniophenyl)amino)-7-(phenylamino)phenothiazin-5-ium chloride** **(25)**

Yield 0.117 g (84%), Mp: 205 °C. ^1^H NMR (DMSO-*d*_6_, δ, ppm, *J*/Hz): 4.81 (s, 3H, NH_3_^+^), 6.95–7.20 (m, 2H, H(3′), H(5′)), 7.28–7.41 (m, 3H, H(2′), H(6′), H(2)), 7.45 (d, 2H, ^3^*J*_HH_ = 7.9 Hz, H(2″), H(6″)), 7.49–7.93 (m, 6H, H(1), H(9), H(4), H(8), H(4″), H(6)), 8.00–8.10 (m, 2H, H(3″), H(5″)), 10.97 (s, 1H, NH), 11.36 (s, 1H, NH). ^1^H NMR (DMSO-*d*_6_ + 2% N_2_H_4_·H_2_O, δ, ppm, *J*/Hz): 6.42 (s, 1H, H(4)), 6.47–6.50 (m, 4H, H(2′), H(6′), H(3′), H(5′)), 6.60 (d, 1H, ^3^*J*_HH_ = 8.5 Hz, H(2)), 6.63–6.75(m, 5H, H(4″), H(1), H(9), H(8), H(6)), 6.84 (d, 2H, ^3^*J*_HH_ = 8.0 Hz, H(2″), H(6″)), 7.13 (t, 2H, ^3^*J*_HH_ = 7.8 Hz, H(3″), H(5″)), 7.24 (s, 2H, NH_2_), 7.88 (s, 1H, NH), 8.21 (s, 1H, NH). ^13^C NMR (DMSO-*d*_6_, δ, ppm): 116.0, 116.1, 118.9, 120.4, 121.6, 123.9, 129.3, 133.2, 136.1, 138.7, 140.7, 143.1, 143.2. FTIR ATR (ν/cm^−1^): 3198 (NH_3_^+^), 1587 (C-N), 1483 (C = S^+^), 1381, 1130 (C-N). HRMS (ESI): calculated [M–HCl–Cl^−^]^+^ *m*/*z* = 395.1325, found [M–HCl–Cl^−^]^+^ *m*/*z* = 395.1325. El. Anal. found (%): C, 61.59; H, 4.31; Cl, 15.08; N, 12.07; S, 6.95. C_24_H_20_Cl_2_N_4_S. Calculated (%): C, 61.67; H, 4.31; Cl, 15.17; N, 11.99; S, 6.86.

### 3.7. The Density Functional Theory (DFT) Calculations

The density functional theory (DFT) calculations were performed using the Gaussian 09 program package [45]. For all calculations, the 6-311++G(d,p) basis set was used. All geometries were optimized by applying the B3LYP functional both in vacuum and in the presence of a solvent (IEFPCM solvent effect model). The stationary points on the potential energy surfaces were located by full geometry optimization with the calculation of force constants. The absence of imaginary frequencies suggested that the molecules are at the minimum of potential energy. No symmetry restrictions were applied during the geometry optimization. Calculations of electronic absorption spectra were performed using TD-DFT. We calculated the first 50 states on the ground state geometries. The spectral lines were plotted using a Gaussian broadening of 0.3 eV half-width at half maximum. The functional was chosen from a benchmark study of different ones-B3LYP, CAM-B3LYP, and M06-HF. The M06-HF functional showed the best reproducibility for both spectral positions and intensities of bands in the spectrum.

## 4. Conclusions

A convenient and versatile approach was developed to the synthesis of 3-*N*′-arylaminophenothiazines and asymmetrical 3,7-di(*N*′-arylamino)phenothiazines as aromatic Methylene Blue isostructural analogues. It was shown that the reaction of the 3-(phenylamino)phenothiazin-5-ium triiodide with a series of arylamines (Route 1) was characterized by higher yields. At same time, the reaction of the 3-*N*′-arylaminophenothiazines with aniline (Route 2) can be used for synthesis of 3,7-di(*N*′-arylamino)phenothiazines with sterically loaded arylamine fragments. Optimization of the synthetic strategy by DFT studies, i.e., quantification analysis of the positive charge distribution in the cation of 3-*N*′-arylaminophenothiazine derivative, was carried out. It was found that the conjugation between aromatic fragments and the mesomeric effect of the substituent affected the further reactivity of 3-*N*′-arylaminophenothiazines in reactions with arylamines. A series of novel 3-*N*′-arylaminophenothiazines and asymmetrical 3,7-di(*N*′-arylamino)phenothiazines containing ester, *tert*-butoxycarbonyl, sulfonic acid, hydroxyl, and amine groups were obtained in high yields. The results obtained can be applied in the design of new arylamino derivatives of phenothiazine in order to “fine tune” non-covalent interactions to obtain materials with the desired photophysical and electrical properties for the utilities of modern organic electronics, sensors, and medicine.

## Figures and Tables

**Table 1:** Hirshfeld charges for sulfur and carbons at the 3 and 7 positions in the cations of the compounds **1** and **10**–**16**.

Compounds	Hirshfeld Charges		
	S	C(3)	C(7)
**1**	0.247	0.028	0.028
**10**	0.186	0.104	0.009
**11**	0.179	0.101	0.006
**12**	0.179	0.105	0.006
**13**	0.194	0.104	0.013
**14**	0.179	0.100	0.007
**15**	0.192	0.105	0.011
**16**	0.184	0.104	0.008

**Table 2:** Selected linear response vertical excitation energies and oscillator strengths calculated (TD-DFT M06-HF/6-311++G(d,p)/IEFPCM) for **16** and **22** in THF.

Dye	λ (nm)	Energy (eV)	Oscillator Strength (au)	Configuration *		%	λ_exp_ (nm)
**16**	534.8	2.318	0.4389	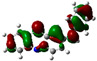	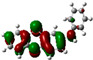	68	497.0
				HOMO → LUMO			
	450.5	2.752	0.3170	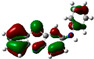	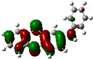	68	
				HOMO-1 → LUMO			
	372.8	3.326	0.0916	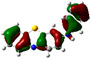	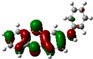	68	365.4
				HOMO-3 → LUMO			
	273.4	4.535	0.1928	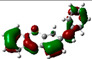	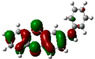	62	287.4
				HOMO-6 → LUMO			
**22**	546.4	2.269	1.2452	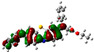	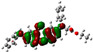	70	533.8
				HOMO → LUMO			
	308.9	4.014	0.0329	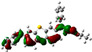	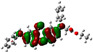	52	301.8
				HOMO-6 → LUMO			
	284.3	4.316	0.1303	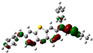	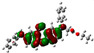	46	286.4
				HOMO-9 → LUMO			
	273.5	4.533	0.6594	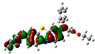	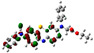	41	
				HOMO → LUMO+4			
	263.9	4.697	0.5330	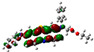	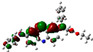	38	
				HOMO-1 → LUMO+1			

*—Major contributions from one-electron excitations to the transitions.

## Data Availability

Data is contained within the article or supplementary material.

## Supplementary Materials

The following supporting information can be downloaded at: https://www.mdpi.com/article/10.3390/molecules27093024/s1, Figures S1–S68: ^1^H, ^13^C NMR, FT-IR, HRMS spectra of the compounds **10**–**25**; Figure S69: UV-Vis spectra of the compounds **10**, **11**, **12**, **16**, **22**, and **23** (THF, 1 × 10^−5^ M), Figure S70. Calculated (TD-DFT M06-HF/6-311++G(d,p)/IEFPCM) UV-Vis absorption spectra of the compounds **10**, **11**, **12**, **16**, **22**, and **23** in THF; Table S1: Absolute energies, minimum frequencies and calculated atomic coordinates for cations **1**, **10**–**16** (DFT B3LYP/6-311++G(d,p)).

## Sample Availability

Samples of all obtained compounds are available from the authors.
