# Supplementary material for: Towards Asymmetrical Methylene Blue Analogues: Synthesis and Reactivity of 3-N′-Arylaminophenothiazines
Source: Molecules. 2022 May 8;27(9):3024. doi: 10.3390/molecules27093024 (PMC9103869; doi:10.3390/molecules27093024)
Supplement: Supplementary file 1 [file molecules-27-03024-s001.zip › molecules-1716901-supplementary.pdf]

*Supplementary information*

# Towards asymmetrical Methylene Blue analogues: synthesis and reactivity of 3-*N*'-arylaminophenothiazines

**Alena Khadieva, Mansur Rayanov, Ksenia Shibaeva, Alexandr Piskunov, Pavel Padnya, Ivan Stoikov**

## Content

Figure S1. $^{1}H$ NMR spectrum of the compound 10. . . . . . . . . . . . 3
Figure S2. $^{1}H$ NMR spectrum of the compound 11. . . . . . . . . . . . 3
Figure S3. $^{1}H$ NMR spectrum of leuco form of the compound 11. . . . . . . . . . . 4
Figure S4. $^{1}H$ NMR spectrum of the compound 12. . . . . . . . . . . . 4
Figure S5. $^{1}H$ NMR spectrum of leuco form of the compound 12. . . . . . . . . . . 5
Figure S6. $^{1}H$ NMR spectrum of the compound 13. . . . . . . . . . . . 5
Figure S7. $^{1}H$ NMR spectrum of the compound 14. . . . . . . . . . . . 6
Figure S8. $^{1}H$ NMR spectrum of the compound 15. . . . . . . . . . . . 6
Figure S9. $^{1}H$ NMR spectrum of the compound 16. . . . . . . . . . . . 7
Figure S10. $^{1}H$ NMR spectrum of leuco form of the compound 16. . . . . . . . . . . 7
Figure S11. $^{1}H$ NMR spectrum of leuco form of the compound 17. . . . . . . . . . . 8
Figure S12. $^{1}H$ NMR spectrum of leuco form of the compound 18. . . . . . . . . . . 8
Figure S13. $^{1}H$ NMR spectrum of leuco form of the compound 19. . . . . . . . . . . 9
Figure S14. $^{1}H$ NMR spectrum of leuco form of the compound 20. . . . . . . . . . . 9
Figure S15. $^{1}H$ NMR spectrum of the compound 21. . . . . . . . . . . . 10
Figure S16. $^{1}H$ NMR spectrum of the compound 22. . . . . . . . . . . . 10
Figure S17. $^{1}H$ NMR spectrum of the compound 23. . . . . . . . . . . . 11
Figure S18. $^{1}H$ NMR spectrum of the compound 24. . . . . . . . . . . . 11
Figure S19. $^{1}H$ NMR spectrum of the compound 25. . . . . . . . . . . . 12
Figure S20. $^{1}H$ NMR spectrum of leuco form of the compound 25. . . . . . . . . . . 12
Figure S21. $^{13}C$ NMR spectrum of the compound 10. . . . . . . . . . . . 13
Figure S22. $^{13}C$ NMR spectrum of the compound 11. . . . . . . . . . . . 13
Figure S23. $^{13}C$ NMR spectrum of the compound 12. . . . . . . . . . . . 14
Figure S24. $^{13}C$ NMR spectrum of the compound 13. . . . . . . . . . . . 14
Figure S25. $^{13}C$ NMR spectrum of the compound 14. . . . . . . . . . . . 15
Figure S26. $^{13}C$ NMR spectrum of the compound 15. . . . . . . . . . . . 15
Figure S27. $^{13}C$ NMR spectrum of the compound 16. . . . . . . . . . . . 16
Figure S28. $^{13}C$ NMR spectrum of the compound 17. . . . . . . . . . . . 16
Figure S29. $^{13}C$ NMR spectrum of the compound 18. . . . . . . . . . . . 17
Figure S30. $^{13}C$ NMR spectrum of the compound 19. . . . . . . . . . . . 17
Figure S31. $^{13}C$ NMR spectrum of the compound 20. . . . . . . . . . . . 18
Figure S32. $^{13}C$ NMR spectrum of the compound 21. . . . . . . . . . . . 18
Figure S33. $^{13}C$ NMR spectrum of the compound 22. . . . . . . . . . . . 19
Figure S34. $^{13}C$ NMR spectrum of the compound 23. . . . . . . . . . . . 19
Figure S35. $^{13}C$ NMR spectrum of the compound 24. . . . . . . . . . . . 20

Figure S36. $^{13}$C NMR spectrum of the compound 25. . . . . . . . . . . . . 20
Figure S37. FT-IR spectrum of the compound 10. . . . . . . . . . . . . . 21
Figure S38. FT-IR spectrum of the compound 11. . . . . . . . . . . . . . 21
Figure S39. FT-IR spectrum of the compound 12. . . . . . . . . . . . . . 22
Figure S40. FT-IR spectrum of the compound 13. . . . . . . . . . . . . . 22
Figure S41. FT-IR spectrum of the compound 14. . . . . . . . . . . . . . 23
Figure S42. FT-IR spectrum of the compound 15. . . . . . . . . . . . . . 23
Figure S43. FT-IR spectrum of the compound 16. . . . . . . . . . . . . . 24
Figure S44. FT-IR spectrum of the compound 17. . . . . . . . . . . . . . 24
Figure S45. FT-IR spectrum of the compound 18. . . . . . . . . . . . . . 25
Figure S46. FT-IR spectrum of the compound 19. . . . . . . . . . . . . . 25
Figure S47. FT-IR spectrum of the compound 20. . . . . . . . . . . . . . 26
Figure S48. FT-IR spectrum of the compound 21. . . . . . . . . . . . . . 26
Figure S49. FT-IR spectrum of the compound 22. . . . . . . . . . . . . . 27
Figure S50. FT-IR spectrum of the compound 23. . . . . . . . . . . . . . 27
Figure S51. FT-IR spectrum of the compound 24. . . . . . . . . . . . . . 28
Figure S52. FT-IR spectrum of the compound 25. . . . . . . . . . . . . . 28
Figure S53. HRMS spectrum of the compound 10. . . . . . . . . . . . . . 29
Figure S54. HRMS spectrum of the compound 11. . . . . . . . . . . . . . 29
Figure S55. HRMS spectrum of the compound 12. . . . . . . . . . . . . . 30
Figure S56. HRMS spectrum of the compound 13. . . . . . . . . . . . . . 30
Figure S57. HRMS spectrum of the compound 14. . . . . . . . . . . . . . 31
Figure S58. HRMS spectrum of the compound 15. . . . . . . . . . . . . . 31
Figure S59. HRMS spectrum of the compound 16. . . . . . . . . . . . . . 32
Figure S60. HRMS spectrum of the compound 17. . . . . . . . . . . . . . 32
Figure S61. HRMS spectrum of the compound 18. . . . . . . . . . . . . . 33
Figure S62. HRMS spectrum of the compound 19. . . . . . . . . . . . . . 33
Figure S63. HRMS spectrum of the compound 20. . . . . . . . . . . . . . 34
Figure S64. HRMS spectrum of the compound 21. . . . . . . . . . . . . . 34
Figure S65. HRMS spectrum of the compound 22. . . . . . . . . . . . . . 35
Figure S66. HRMS spectrum of the compound 23. . . . . . . . . . . . . . 35
Figure S67. HRMS spectrum of the compound 24. . . . . . . . . . . . . . 36
Figure S68. HRMS spectrum of the compound 25. . . . . . . . . . . . . . 36
Figure S69. UV-Vis spectra of the compounds **10**, **11**, **12**, **16**, **22**, and **23** (THF, $1\times10^{-5}$ M) . . . . . . 36
Figure S70. Calculated (TD-DFT M06-HF/6-311++G(d,p)/IEFPCM) UV-Vis absorption spectra of the compounds **10**, **11**, **12**, **16**, **22**, and **23** in THF . . . . . . . . . . . . . . . . 36
Table S1. Absolute energies, minimum frequencies and calculated atomic coordinates for cations 1, 10-16 (DFT B3LYP/6-311++G(d,p)). . . . . . . . . . . . . . . . . . 38

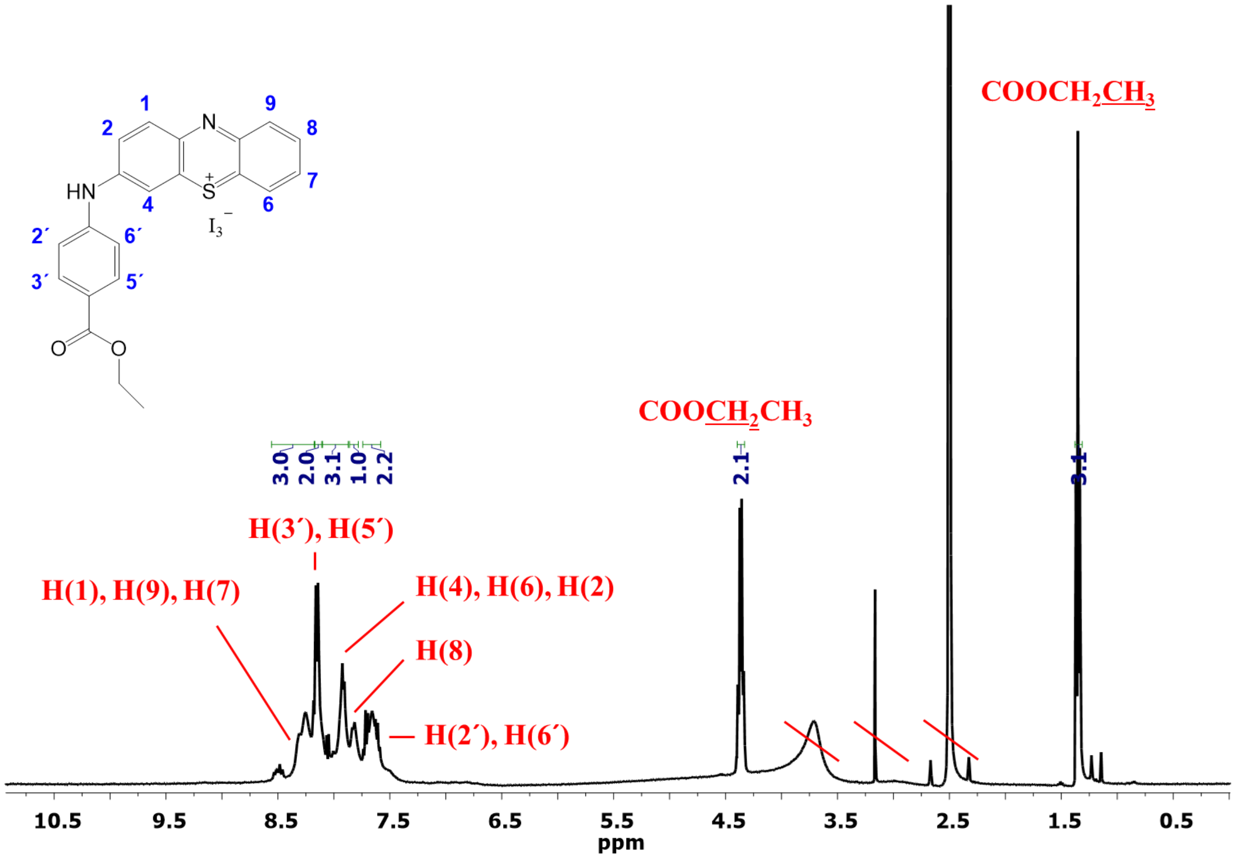


Figure S1. $^{1}$H NMR spectrum of the compound **10**, DMSO-*d*6, 300 K, 400 MHz.

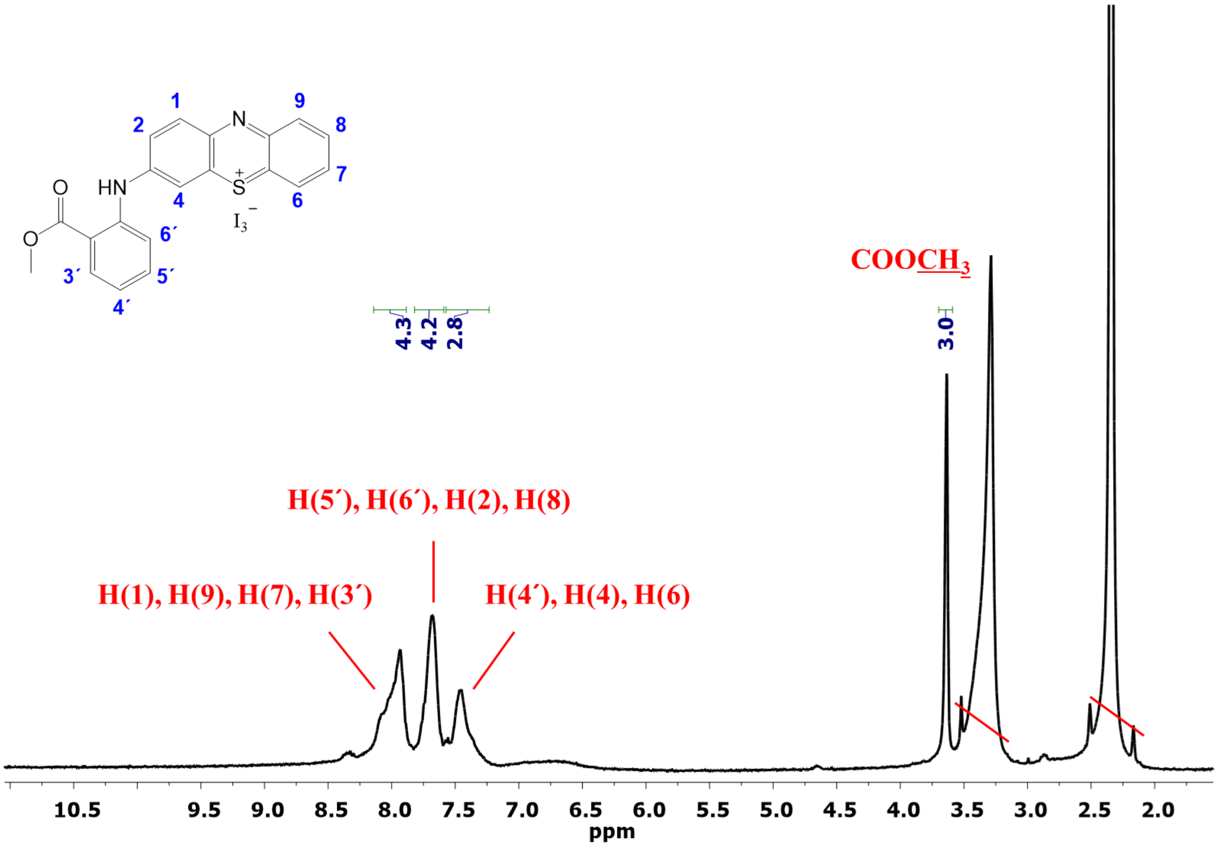


Figure S2. $^{1}$H NMR spectrum of the compound **11**, DMSO-*d*6, 300 K, 400 MHz.

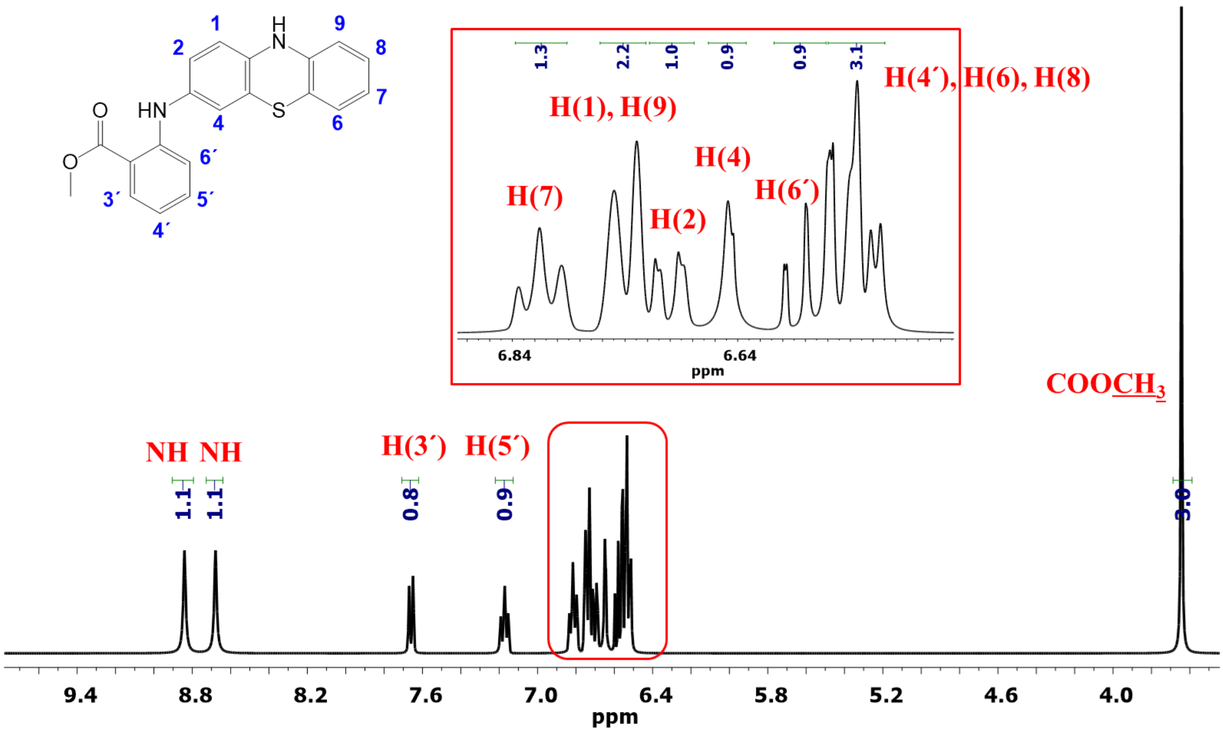


Figure S3. $^{1}$H NMR spectrum of leuco form of the compound **11**, DMSO-$d_6$ + 2 % $N_2H_4{\cdot}H_2O$, 300 K, 400 MHz.

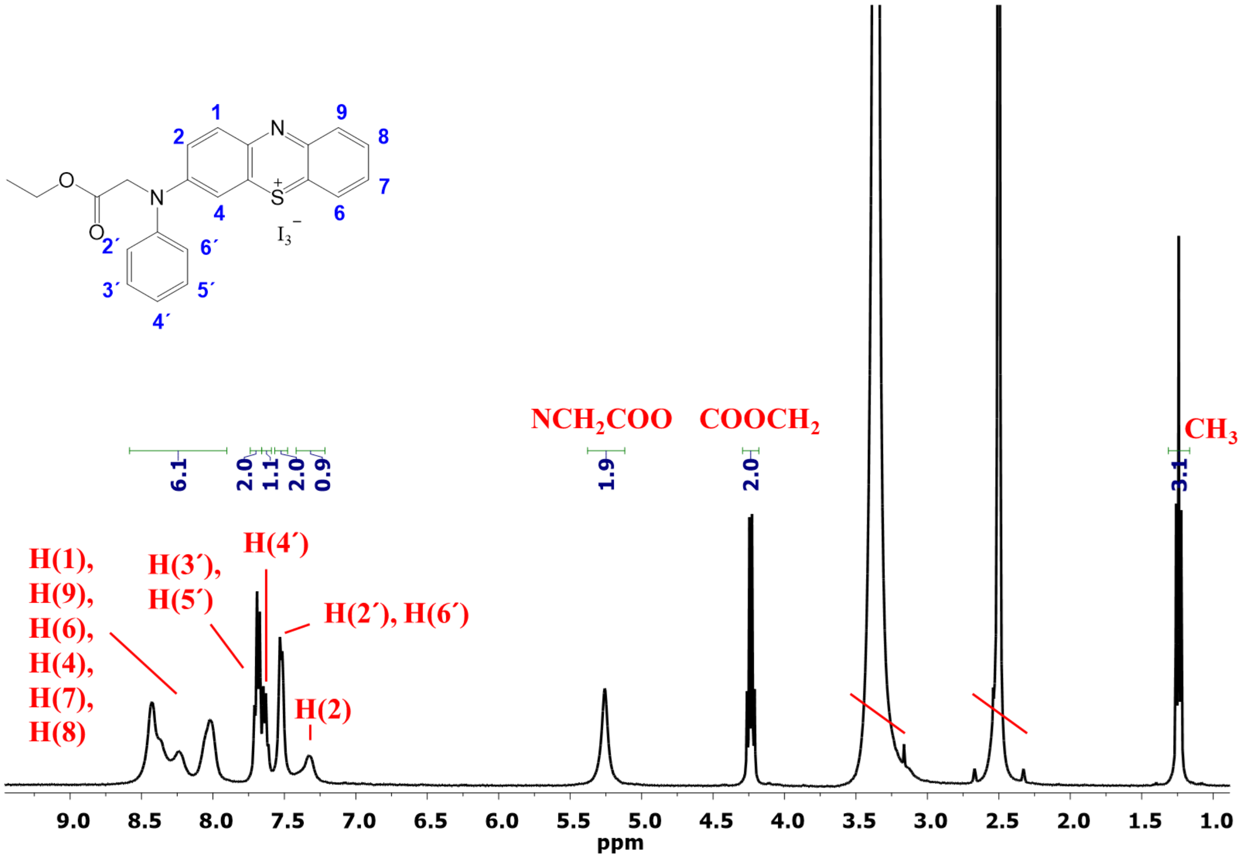


Figure S4. $^{1}$H NMR spectrum of the compound **12**, DMSO-$d_6$, 300 K, 400 MHz.

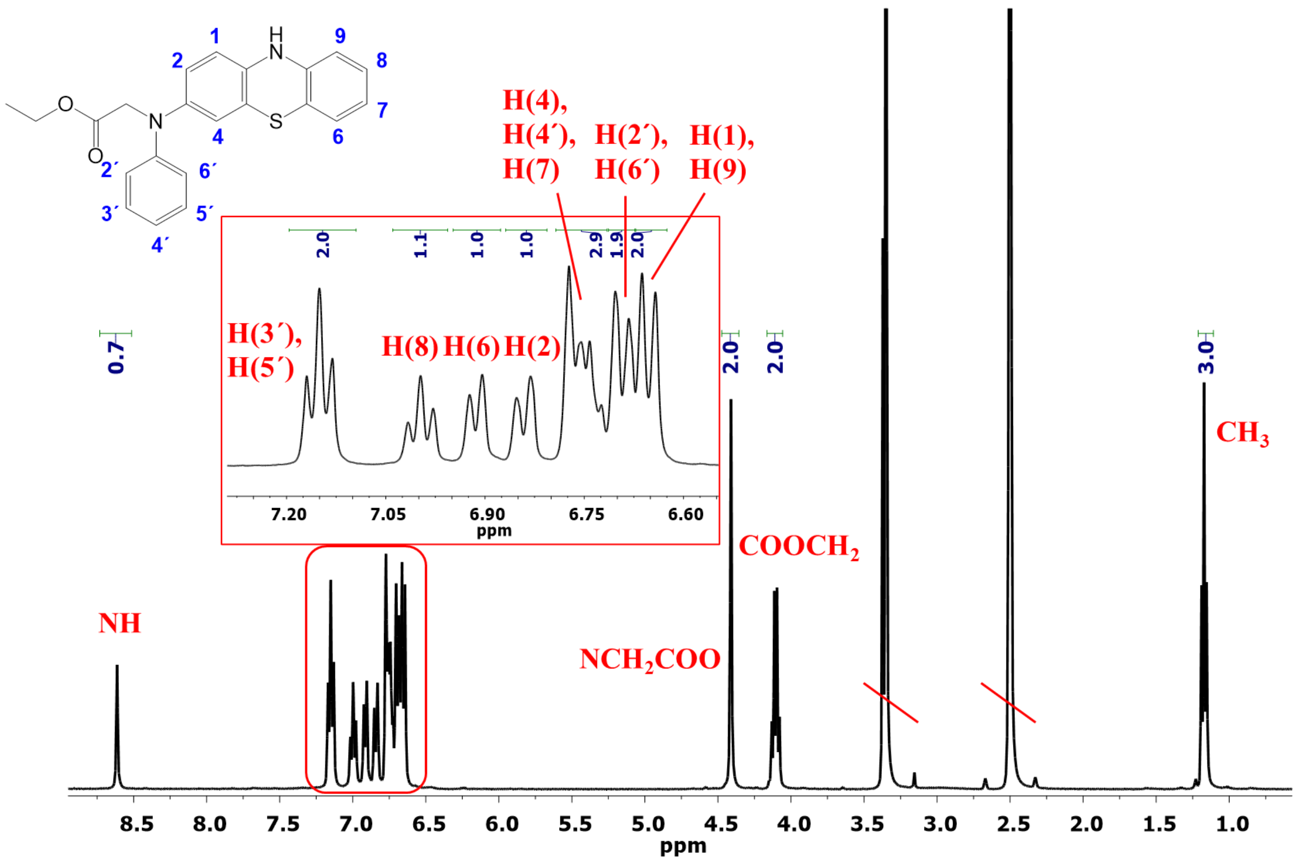


Figure S5. $^{1}$H NMR spectrum of leuco form of the compound **12**, DMSO-*d*6 + 2 % $N_2H_4{\cdot}H_2O$, 300 K, 400 MHz.

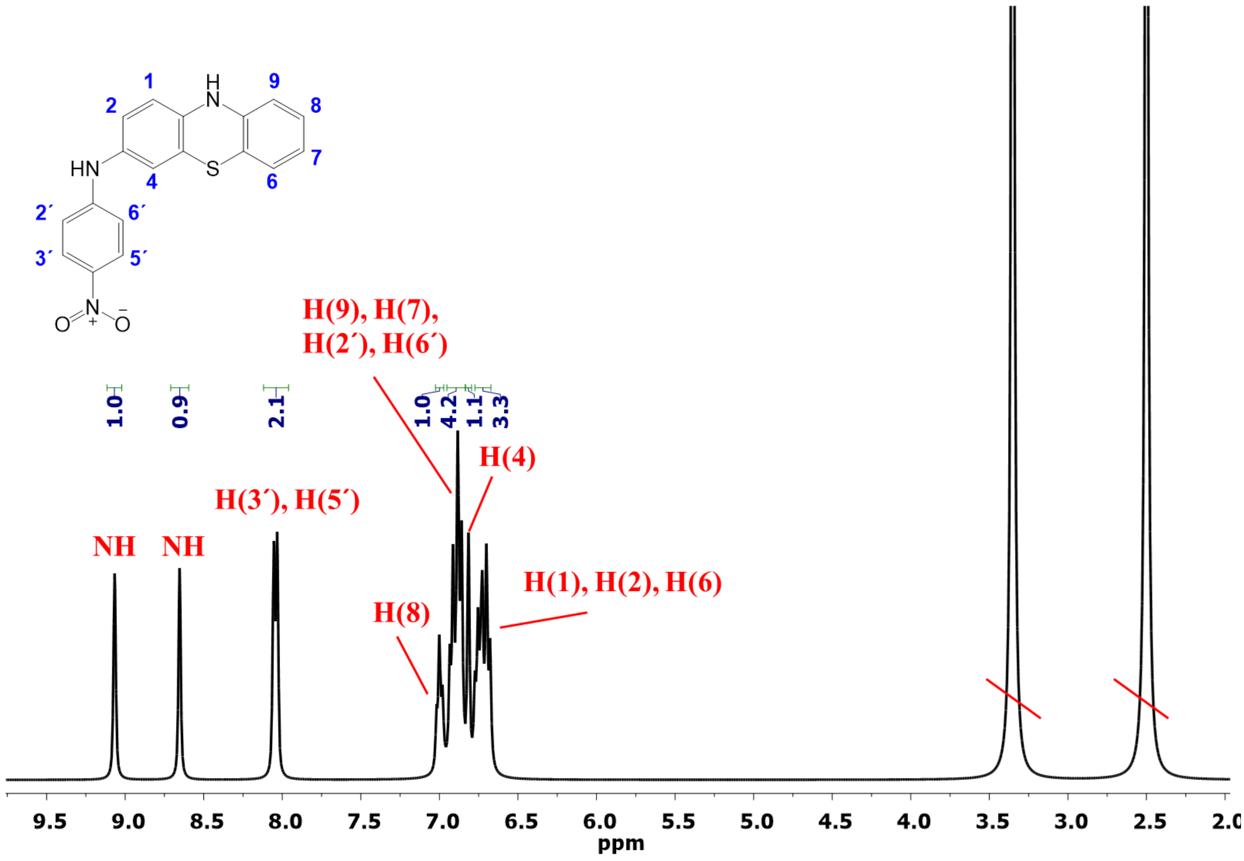


Figure S6. $^{1}$H NMR spectrum of the compound **13**, DMSO-*d*6, 300 K, 400 MHz.

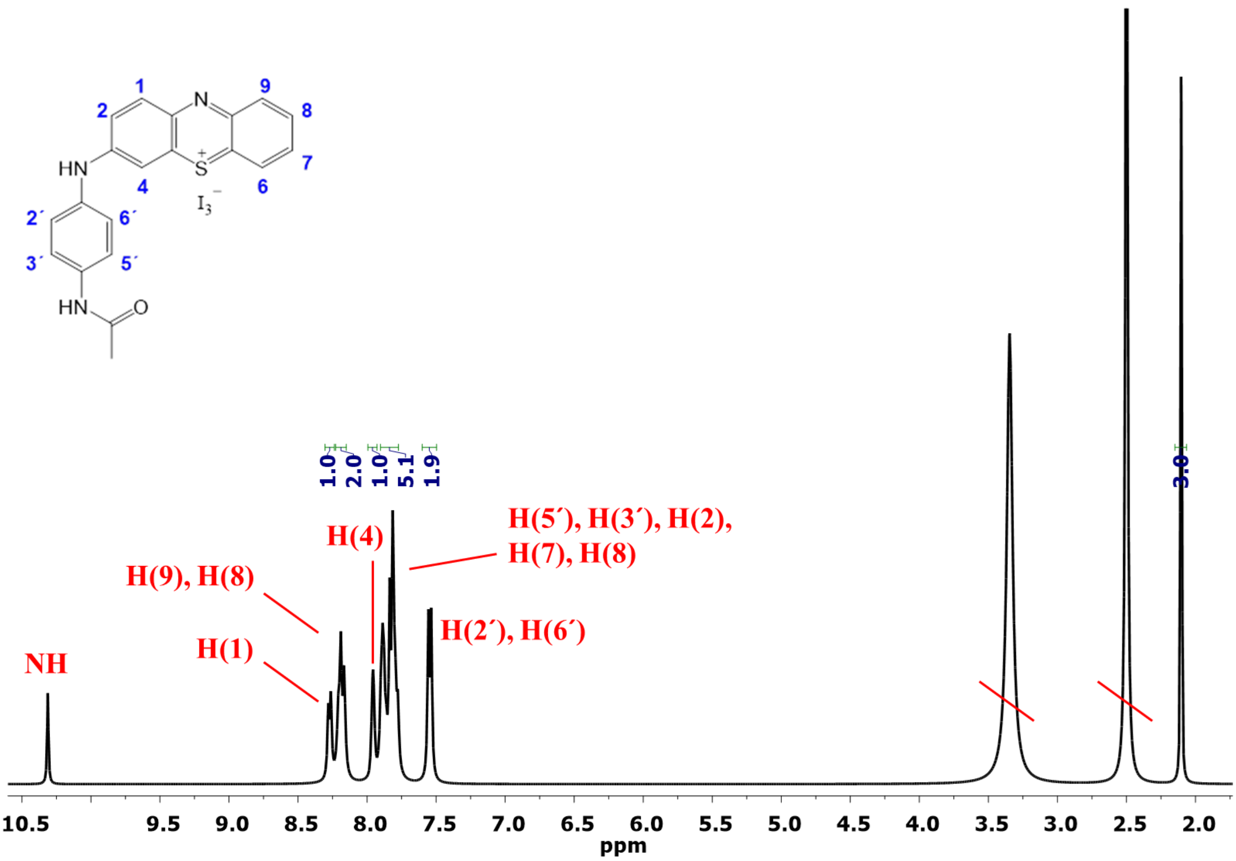


Figure S7. ¹H NMR spectrum of the compound **14**, DMSO-$d_6$, 300 K, 400 MHz.

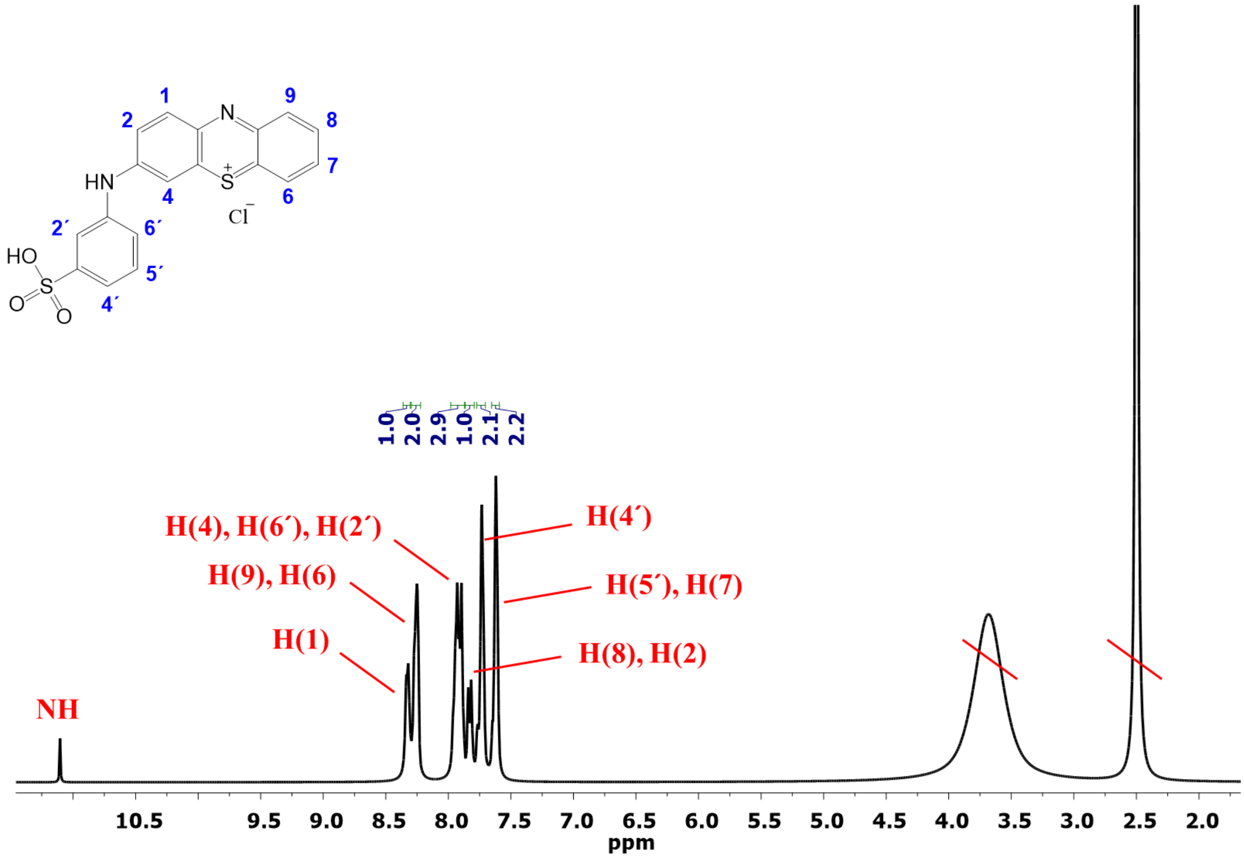


Figure S8. ¹H NMR spectrum of the compound **15**, DMSO-$d_6$, 300 K, 400 MHz.

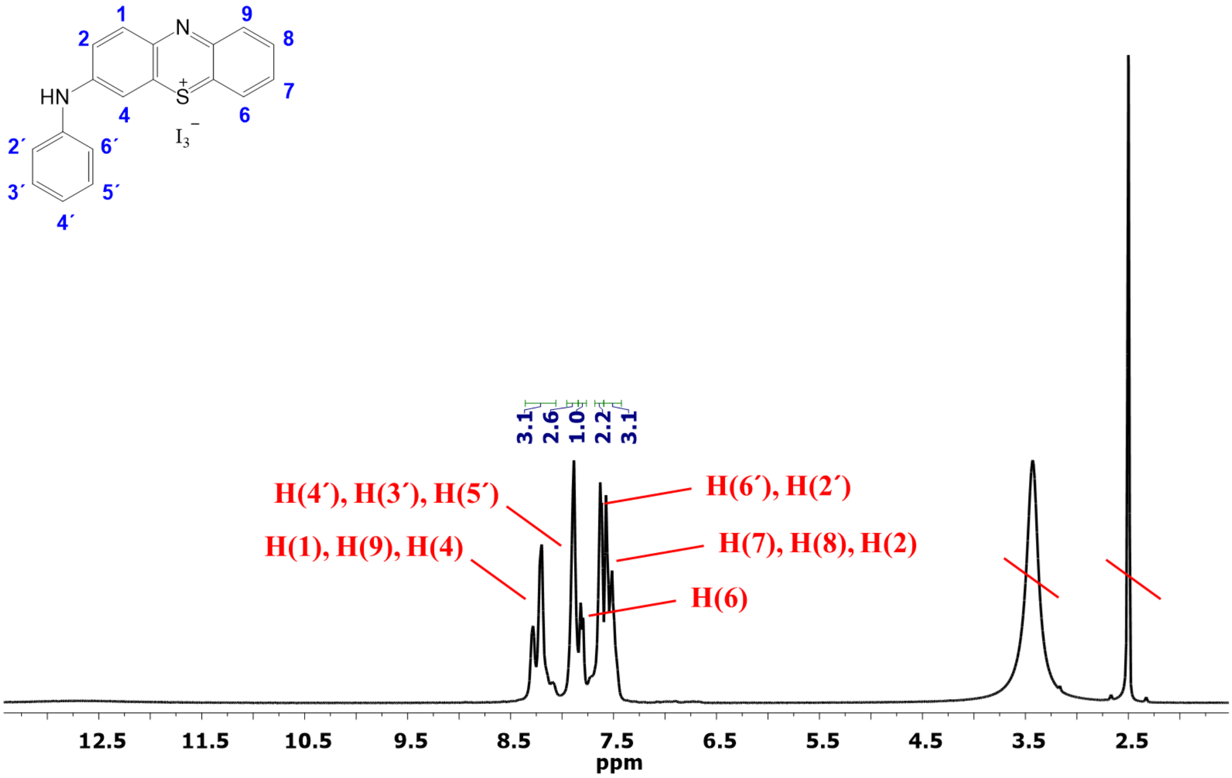


Figure S9. $^{1}$H NMR spectrum of the compound **16**, DMSO-*d*6, 300 K, 400 MHz.

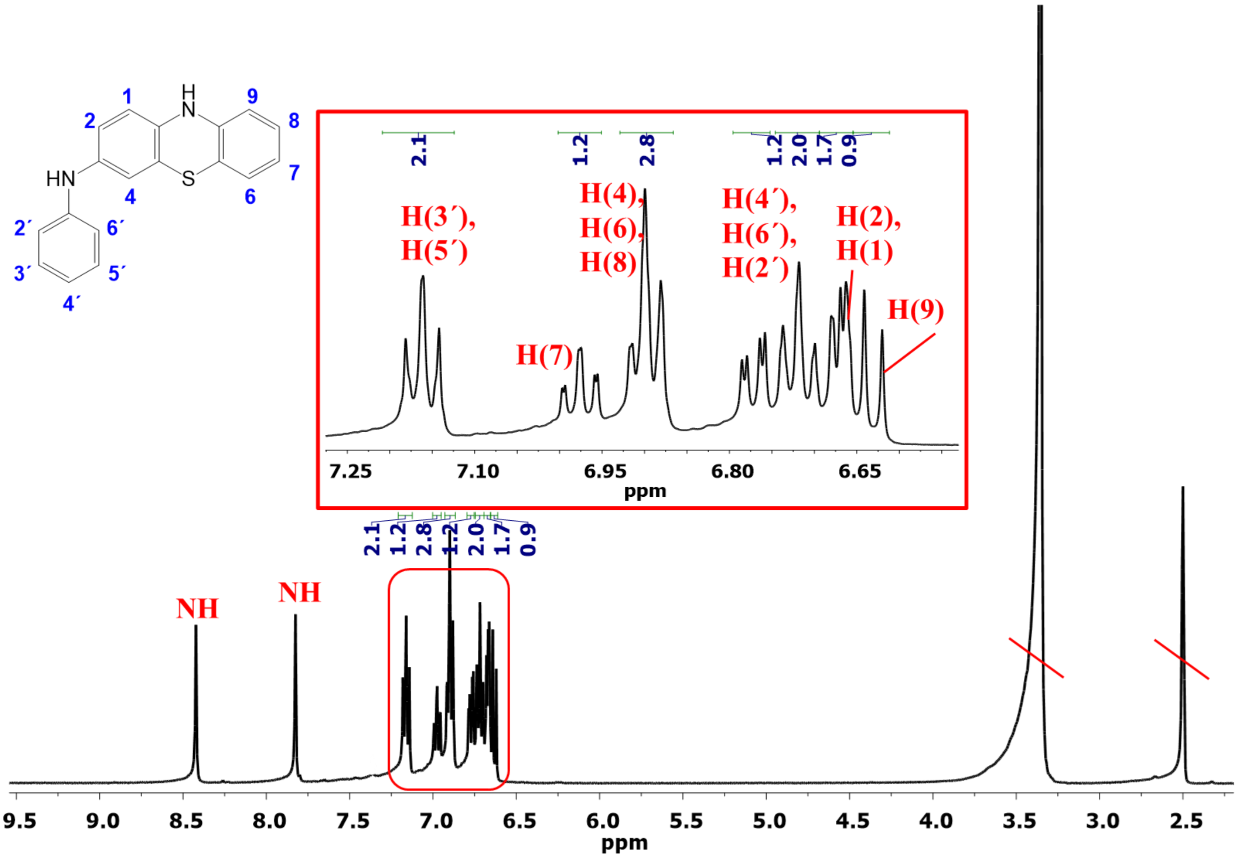


Figure S10. $^{1}$H NMR spectrum of leuco form of the compound **16**, DMSO-*d*6 + 2 % $N_2H_4{\cdot}H_2O$, 300 K, 400 MHz.

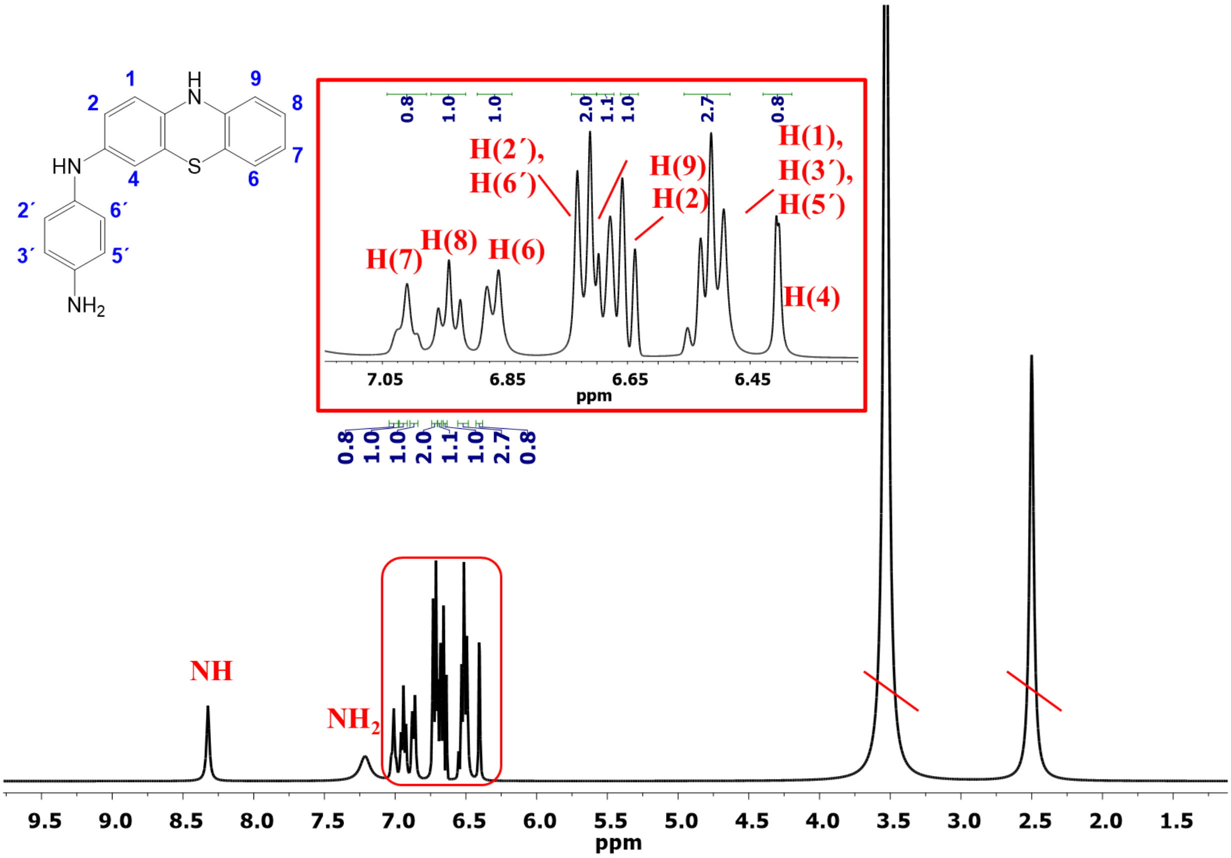


Figure S11. ${}^{1}H$ NMR spectrum of leuco form of the compound **17**, DMSO-$d_6$ + 2 % $N_2H_4 \cdot H_2O$, 300 K, 400 MHz.

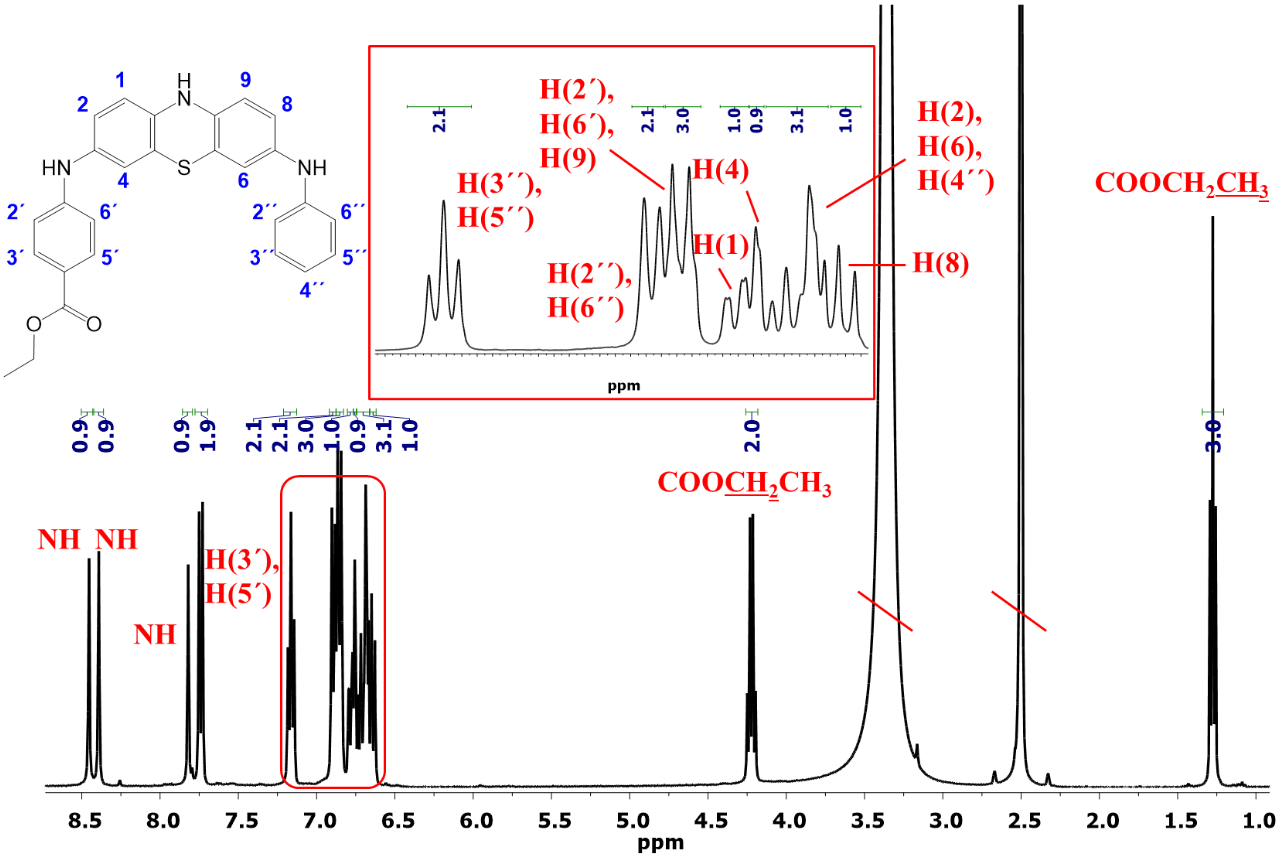


Figure S12. ${}^{1}H$ NMR spectrum of leuco form of the compound **18**, DMSO-$d_6$ + 2 % $N_2H_4 \cdot H_2O$, 300 K, 400 MHz.

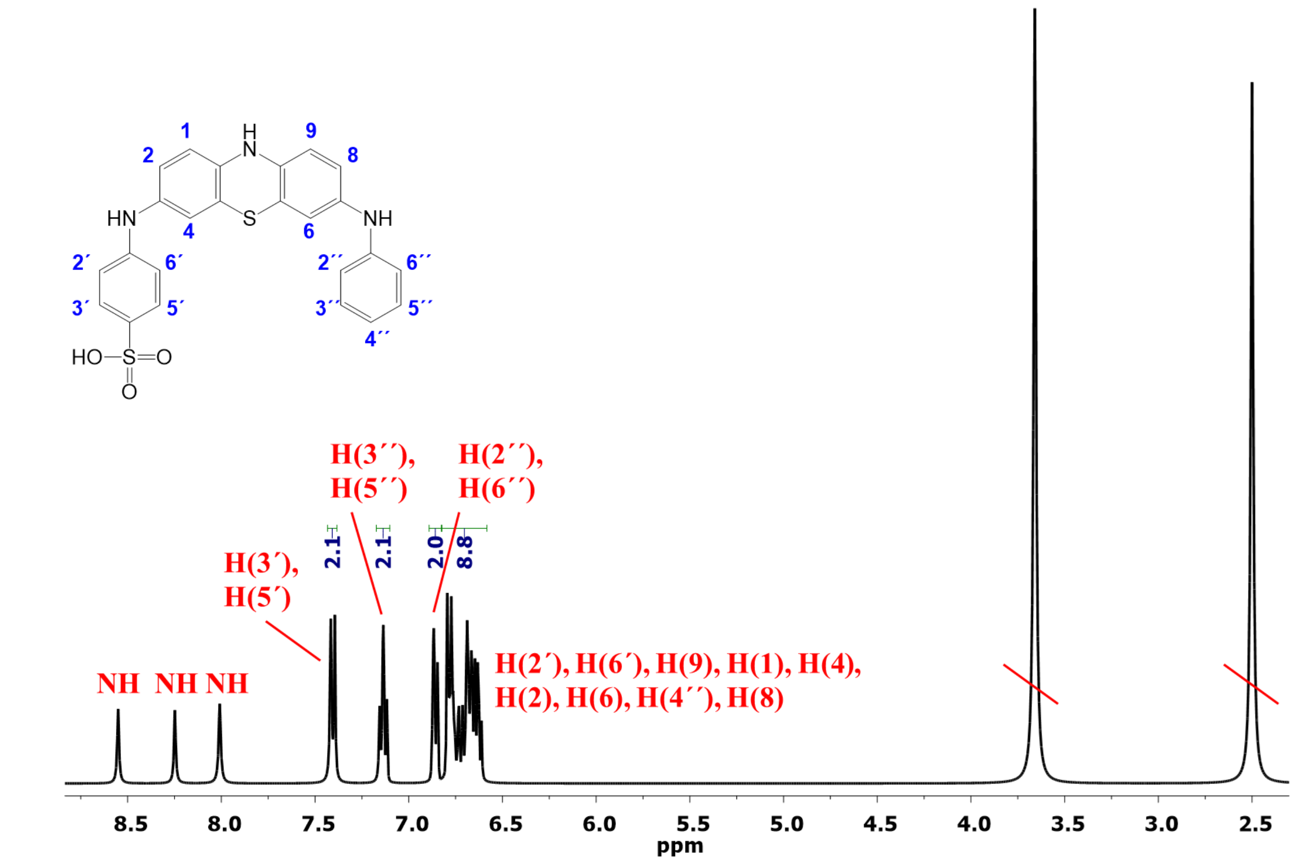


Figure S13. ¹H NMR spectrum of leuco form of the compound **19**, DMSO-$d_6$ + 2 % $N_2H_4 \cdot H_2O$, 300 K, 400 MHz.

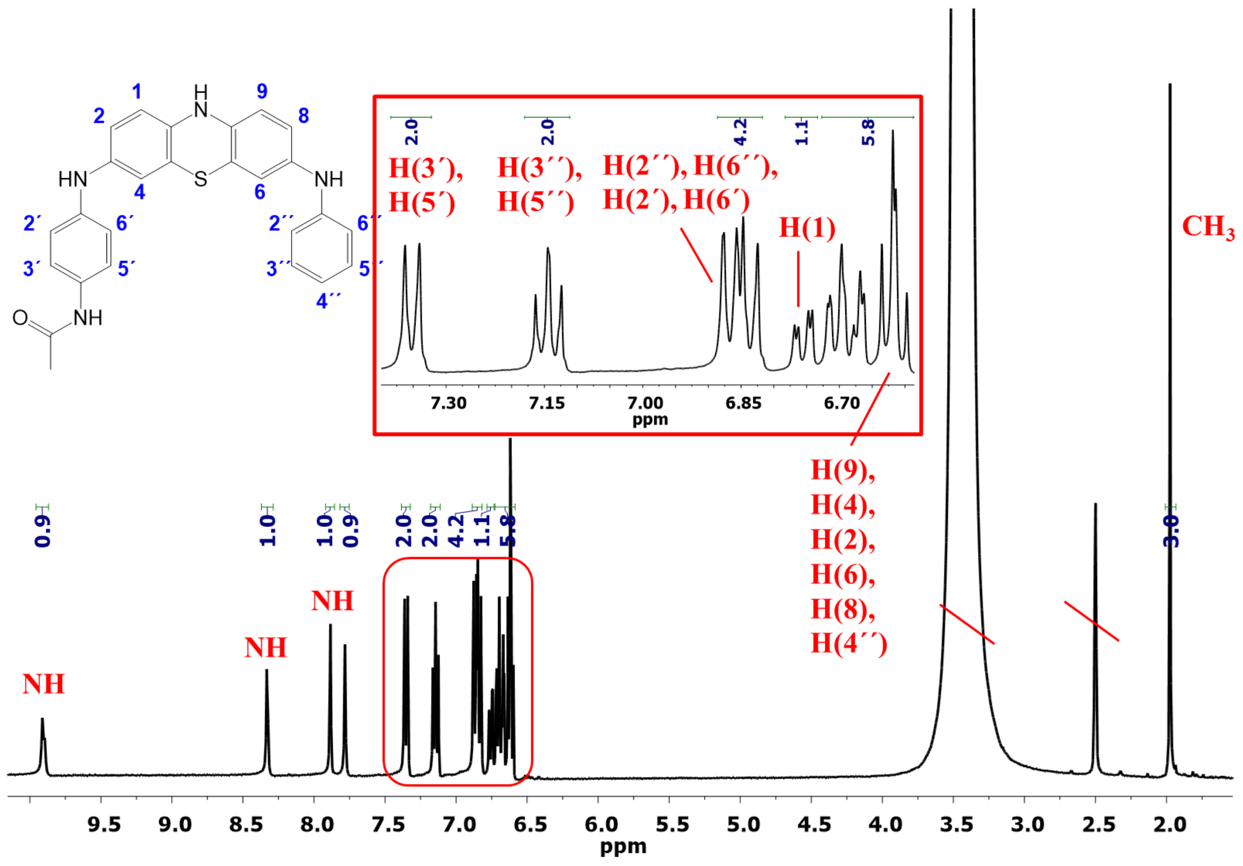


Figure S14. ¹H NMR spectrum of leuco form of the compound **20**, DMSO-$d_6$ + 2 % $N_2H_4 \cdot H_2O$, 300 K, 400 MHz.

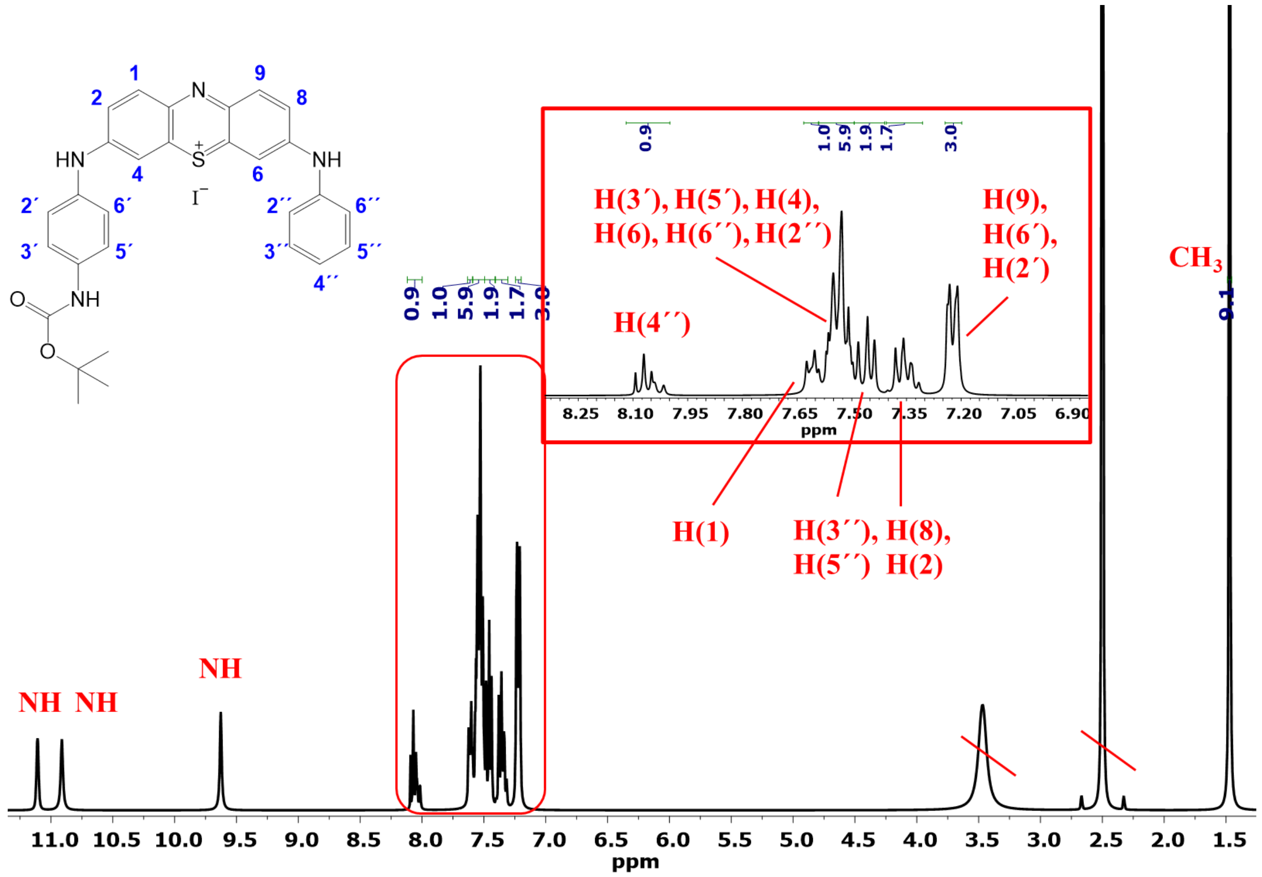


Figure S15. $^{1}$H NMR spectrum of the compound **21**, DMSO-$d_6$, 300 K, 400 MHz.

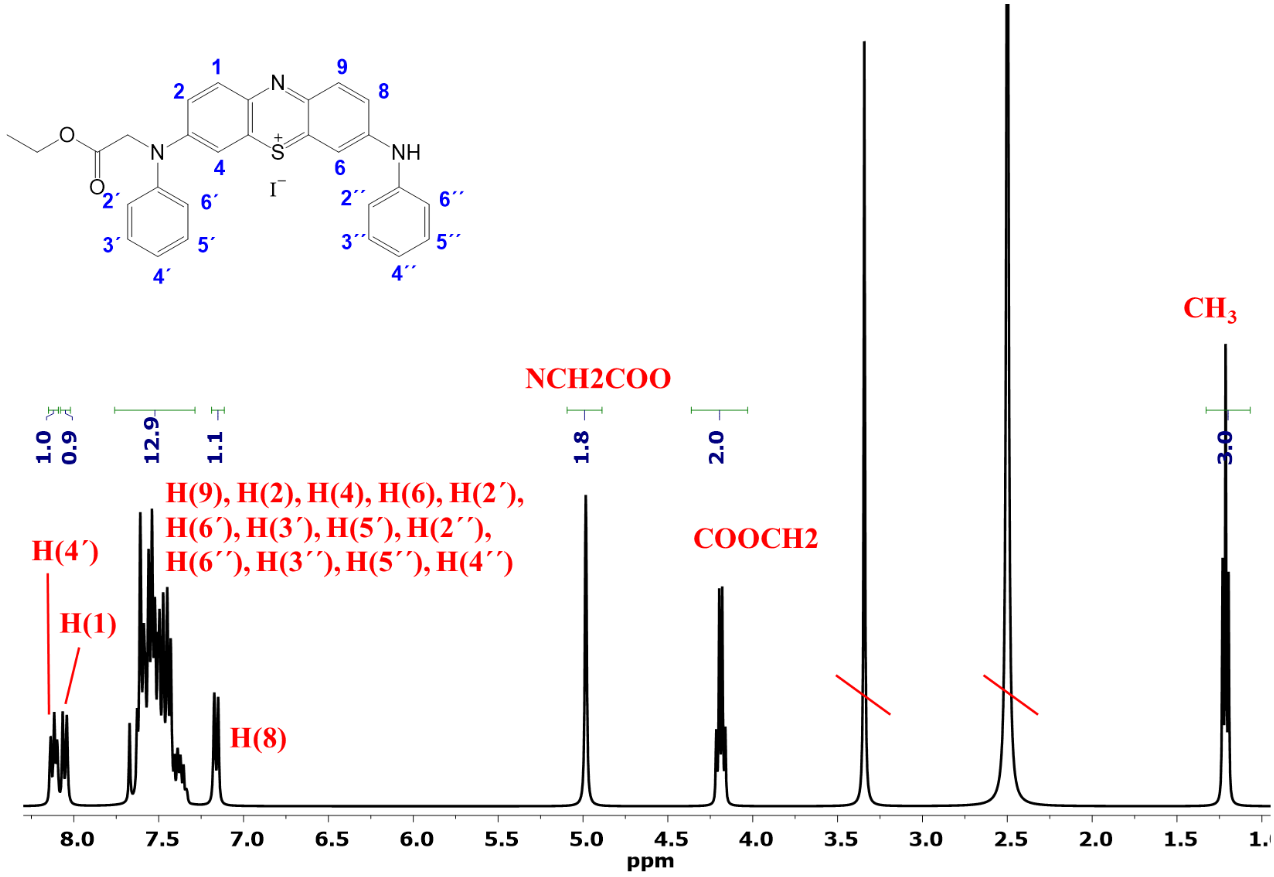


Figure S16. $^{1}$H NMR spectrum of the compound **22**, DMSO-$d_6$, 300 K, 400 MHz.

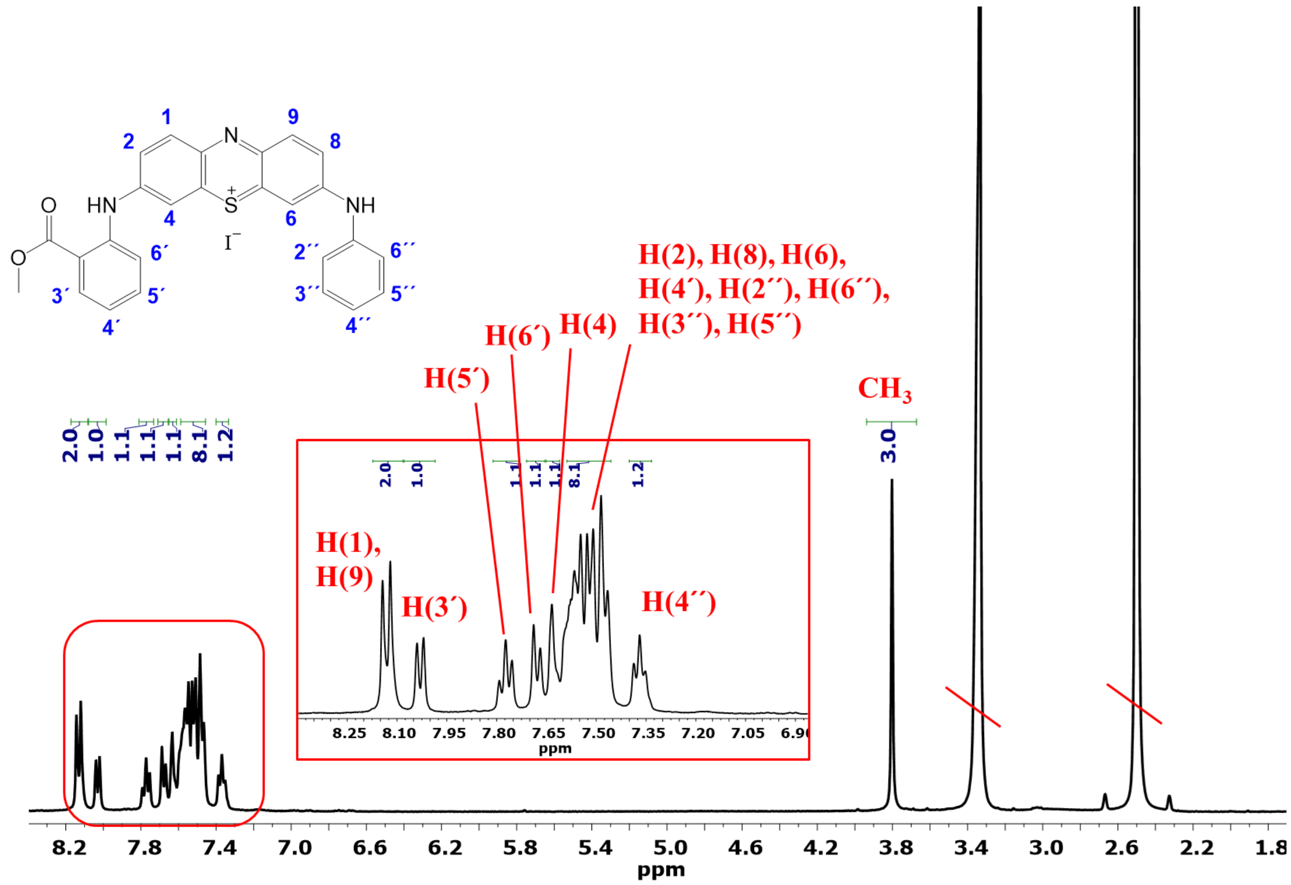


Figure S17. $^{1}$H NMR spectrum of the compound **23**, DMSO-*d*6, 300 K, 400 MHz.

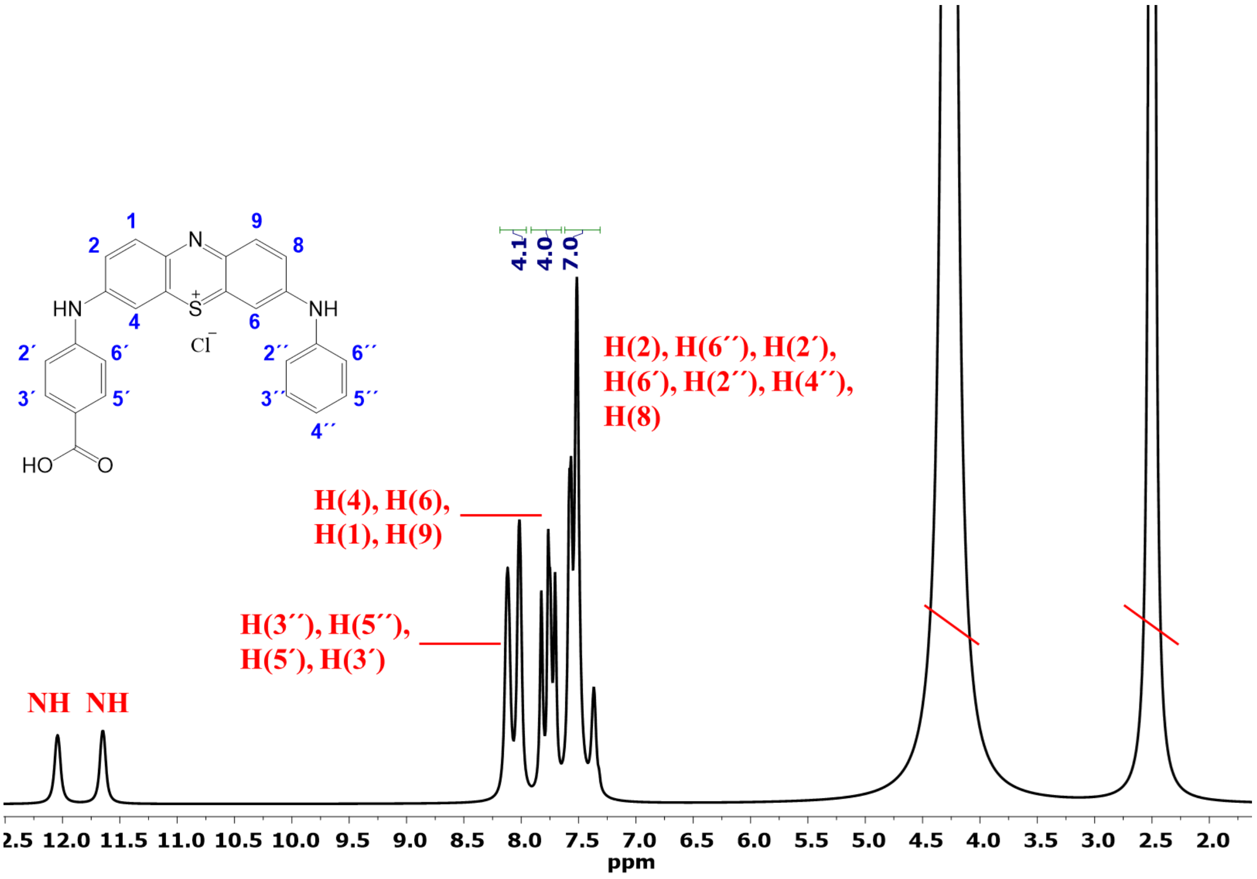


Figure S18. $^{1}$H NMR spectrum of the compound **24**, DMSO-*d*6, 300 K, 400 MHz.

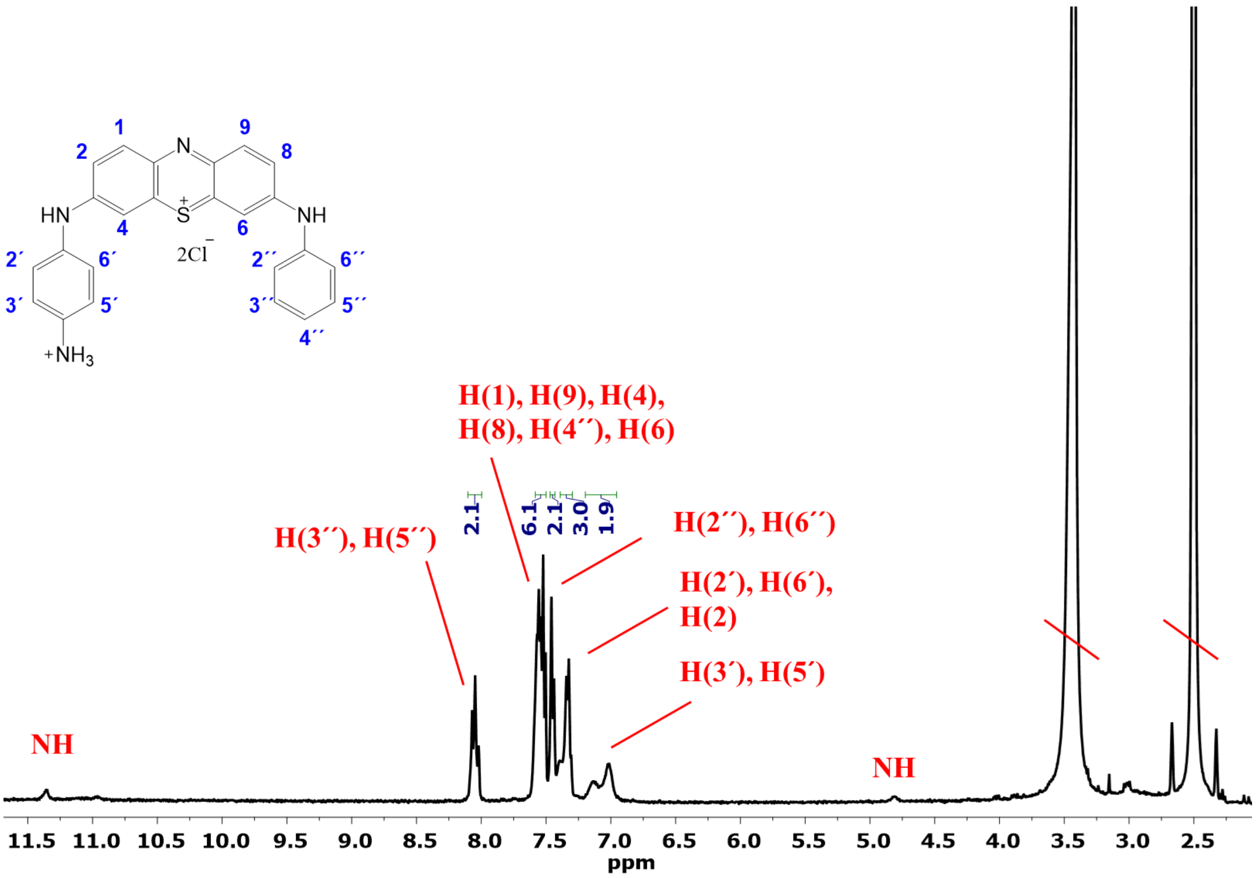


Figure S19. $^{1}$H NMR spectrum of the compound **25**, DMSO-$d_6$, 300 K, 400 MHz.

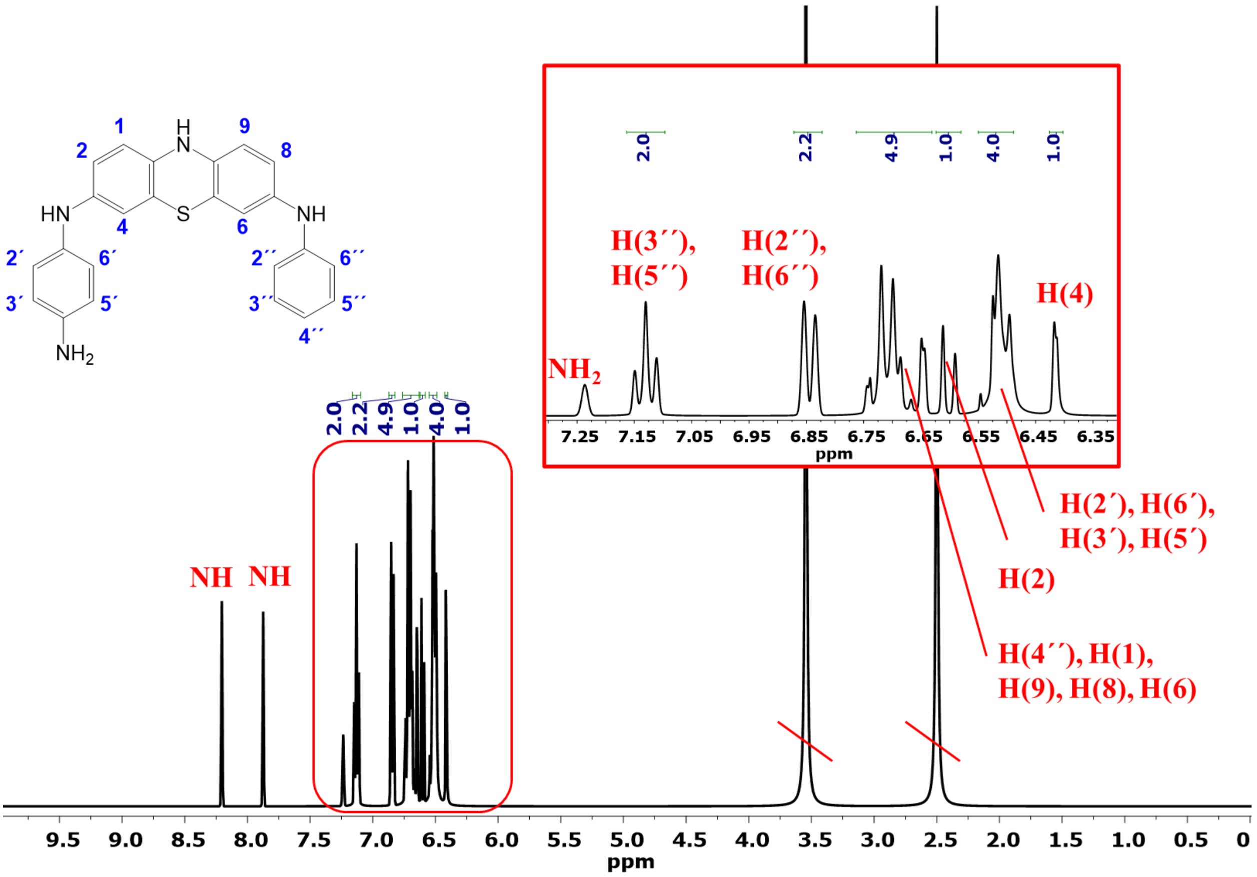


Figure S20. $^{1}$H NMR spectrum of leuco form of the compound **25**, DMSO-$d_6$ + 2 % $N_2H_4 \cdot H_2O$, 300 K, 400 MHz.

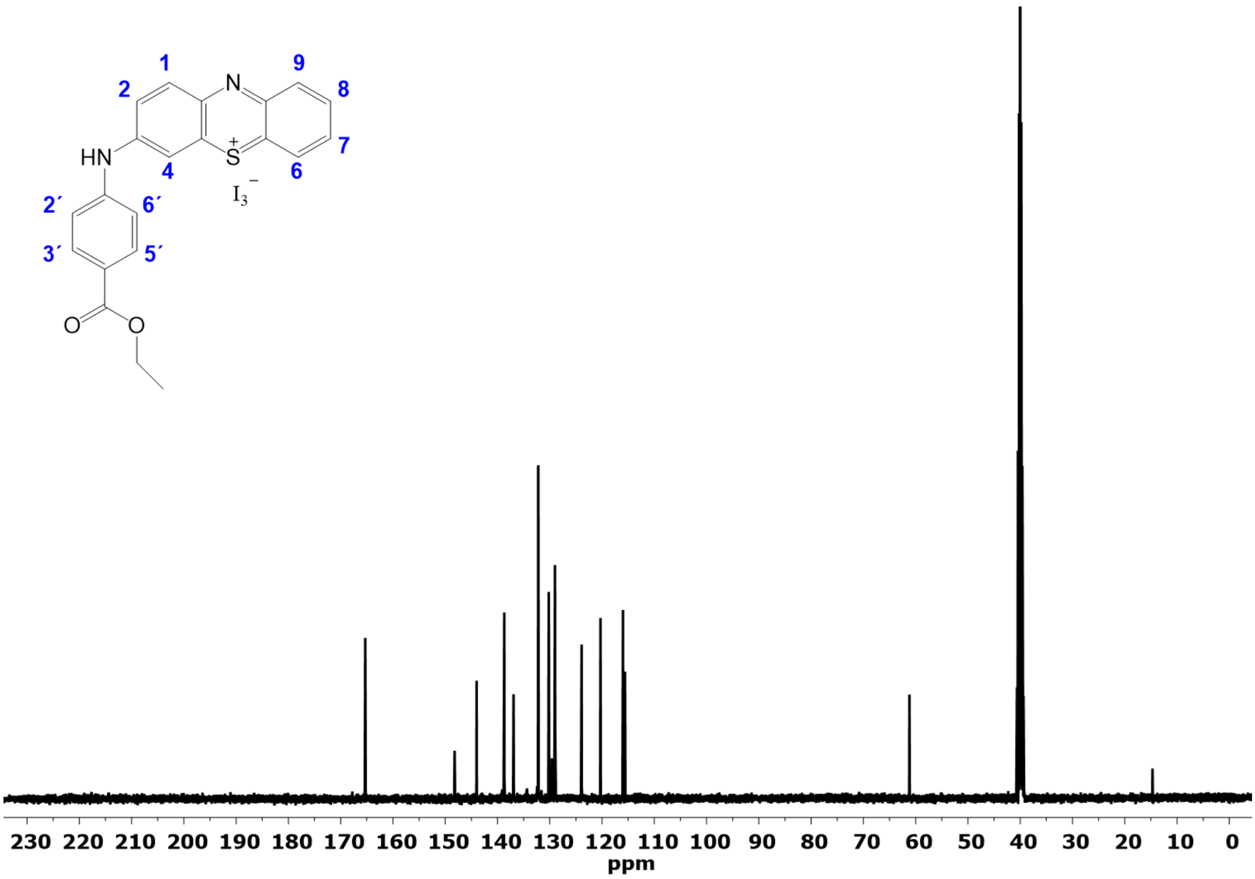


Figure S21. $^{13}C$ NMR spectrum of the compound **10**, DMSO-*d*6, 300 K, 100 MHz.

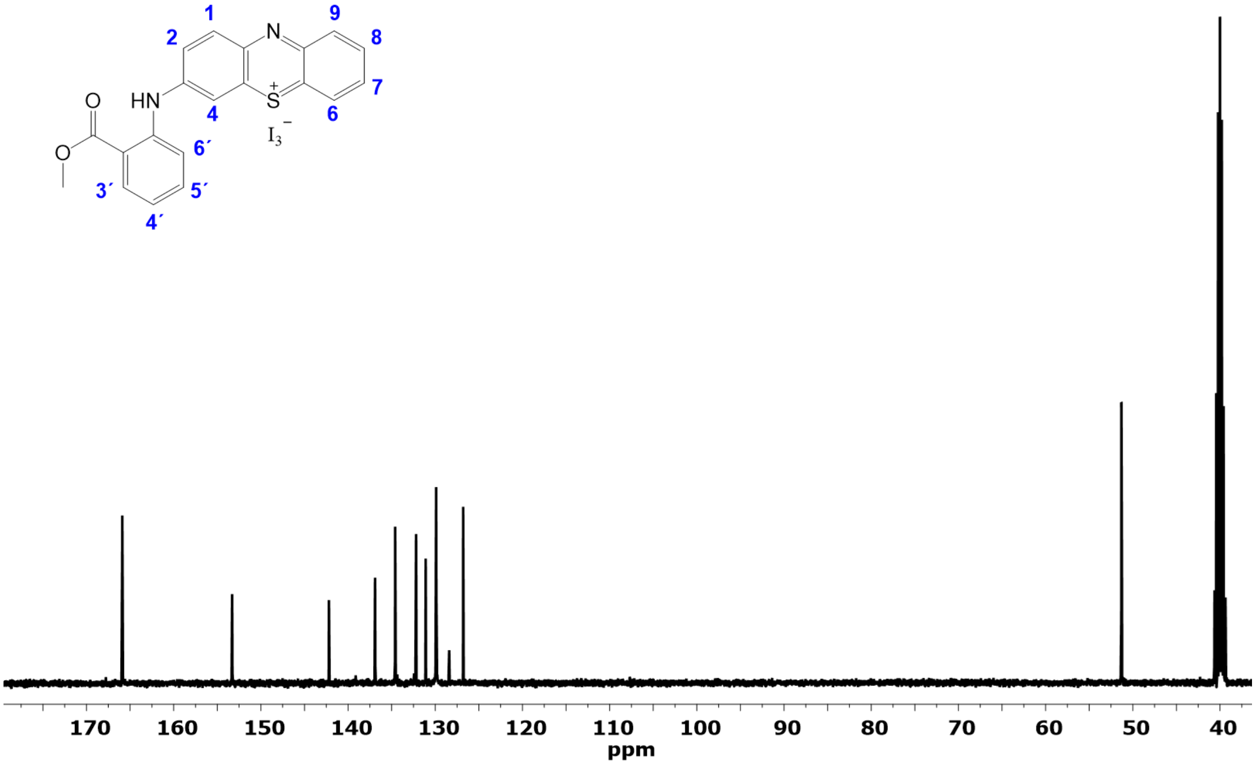


Figure S22. $^{13}C$ NMR spectrum of the compound **11**, DMSO-*d*6, 300 K, 100 MHz.

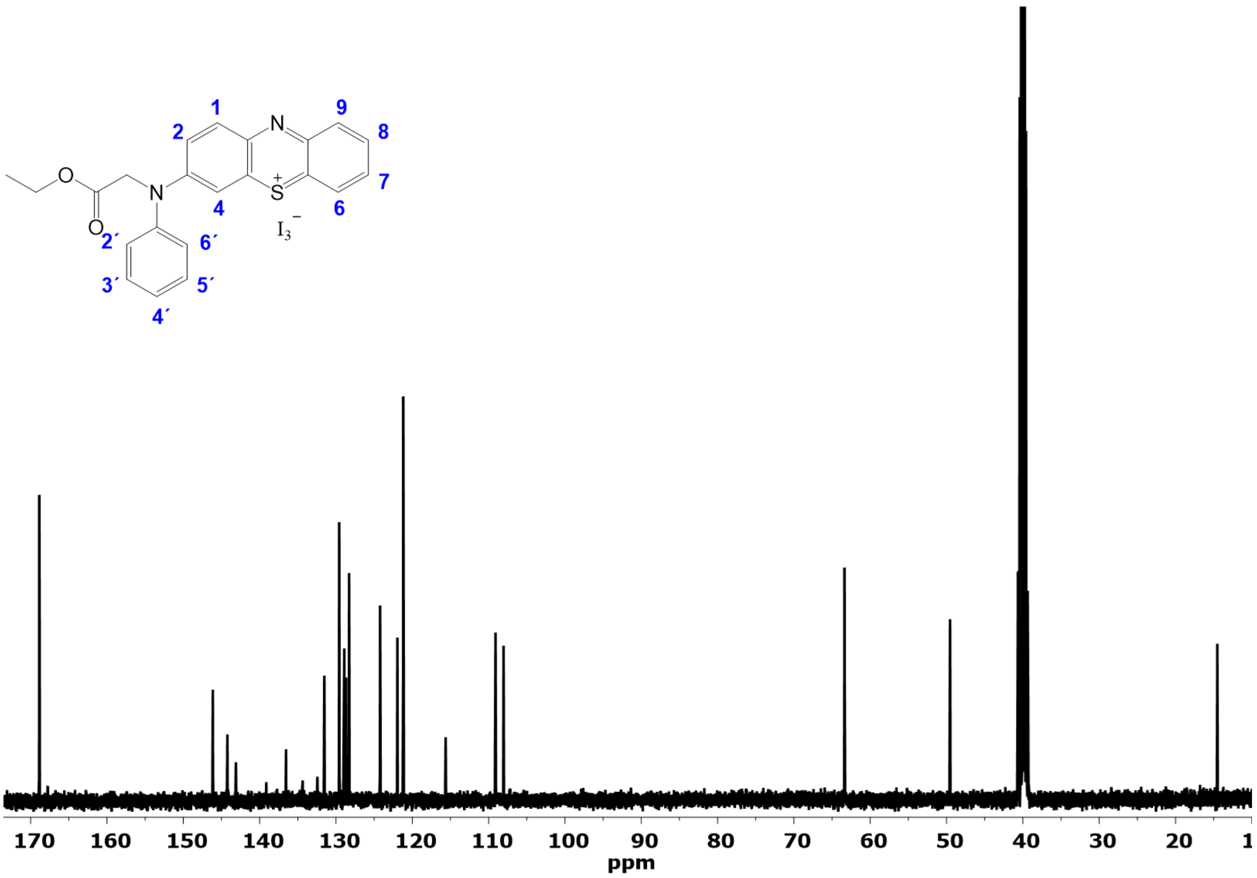


Figure S23. $^{13}$C NMR spectrum of the compound **12**, DMSO-$d_6$, 300 K, 100 MHz.

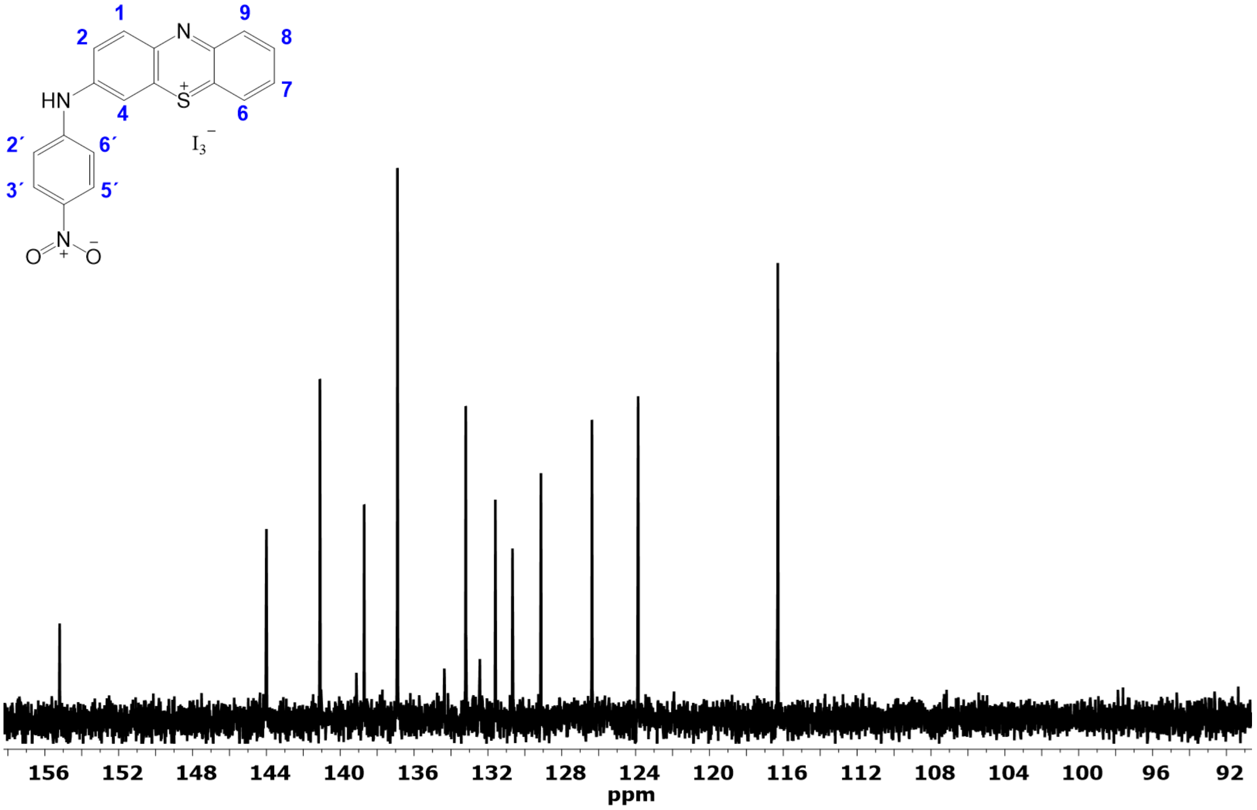


Figure S24. $^{13}$C NMR spectrum of the compound **13**, DMSO-$d_6$, 300 K, 100 MHz.

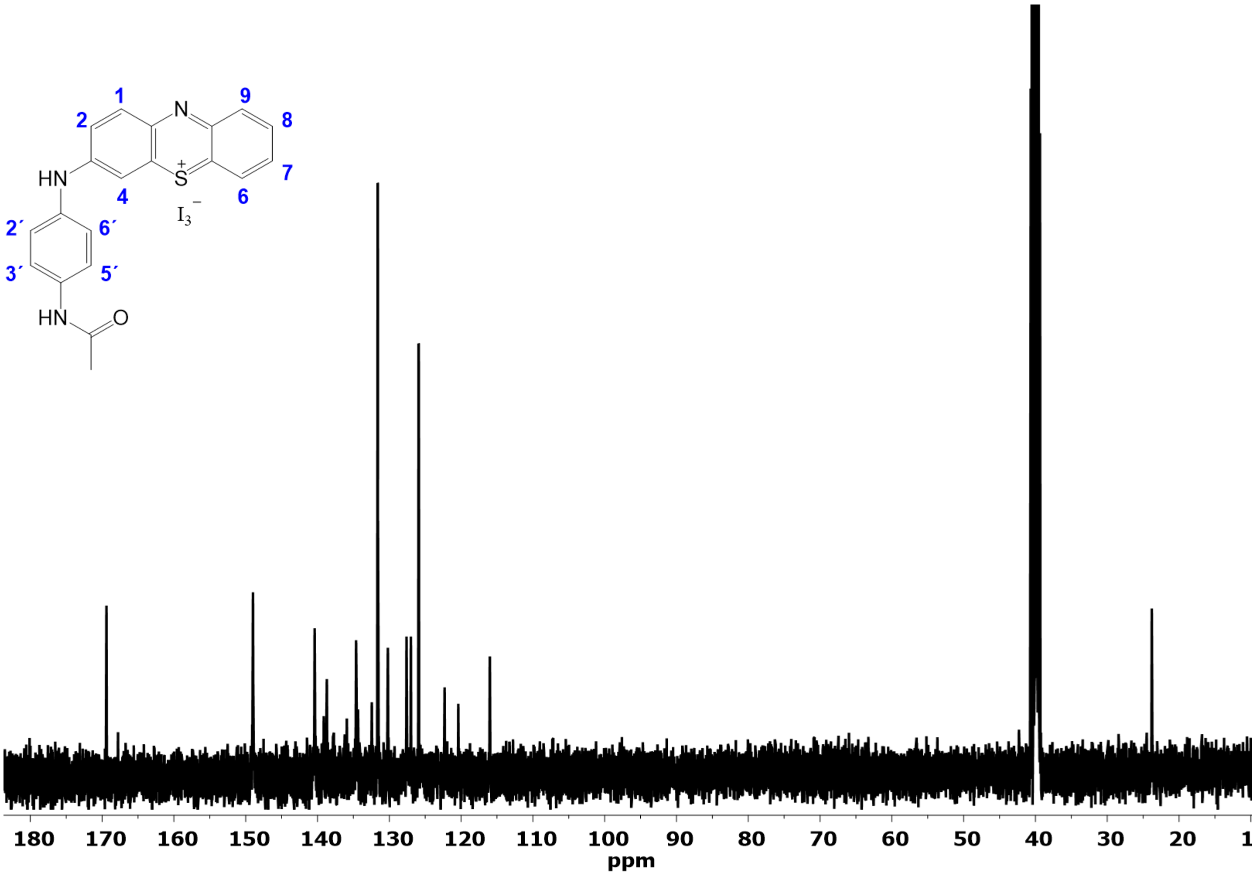


Figure S25. $^{13}$C NMR spectrum of the compound **14**, DMSO-*d*6, 300 K, 100 MHz.

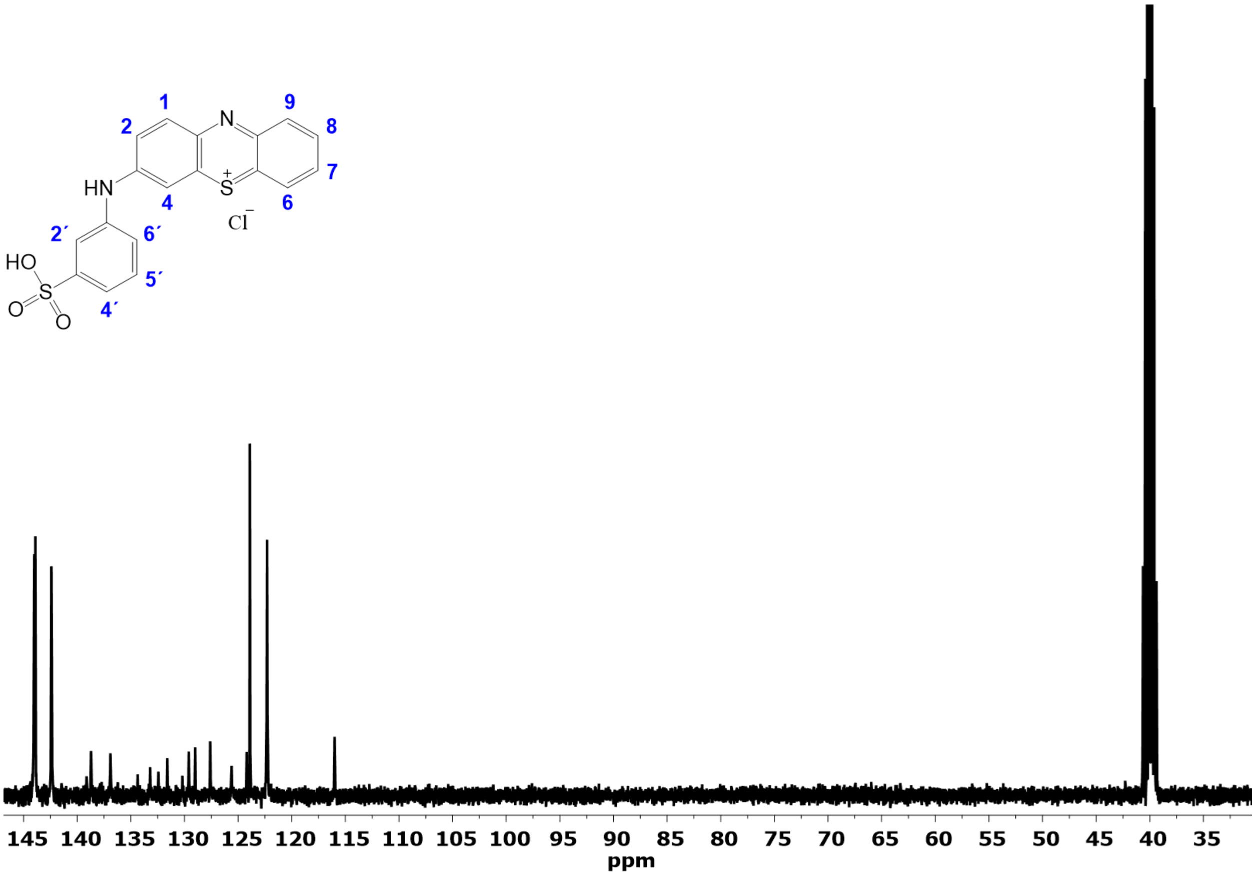


Figure S26. $^{13}$C NMR spectrum of the compound **15**, DMSO-*d*6, 300 K, 100 MHz.

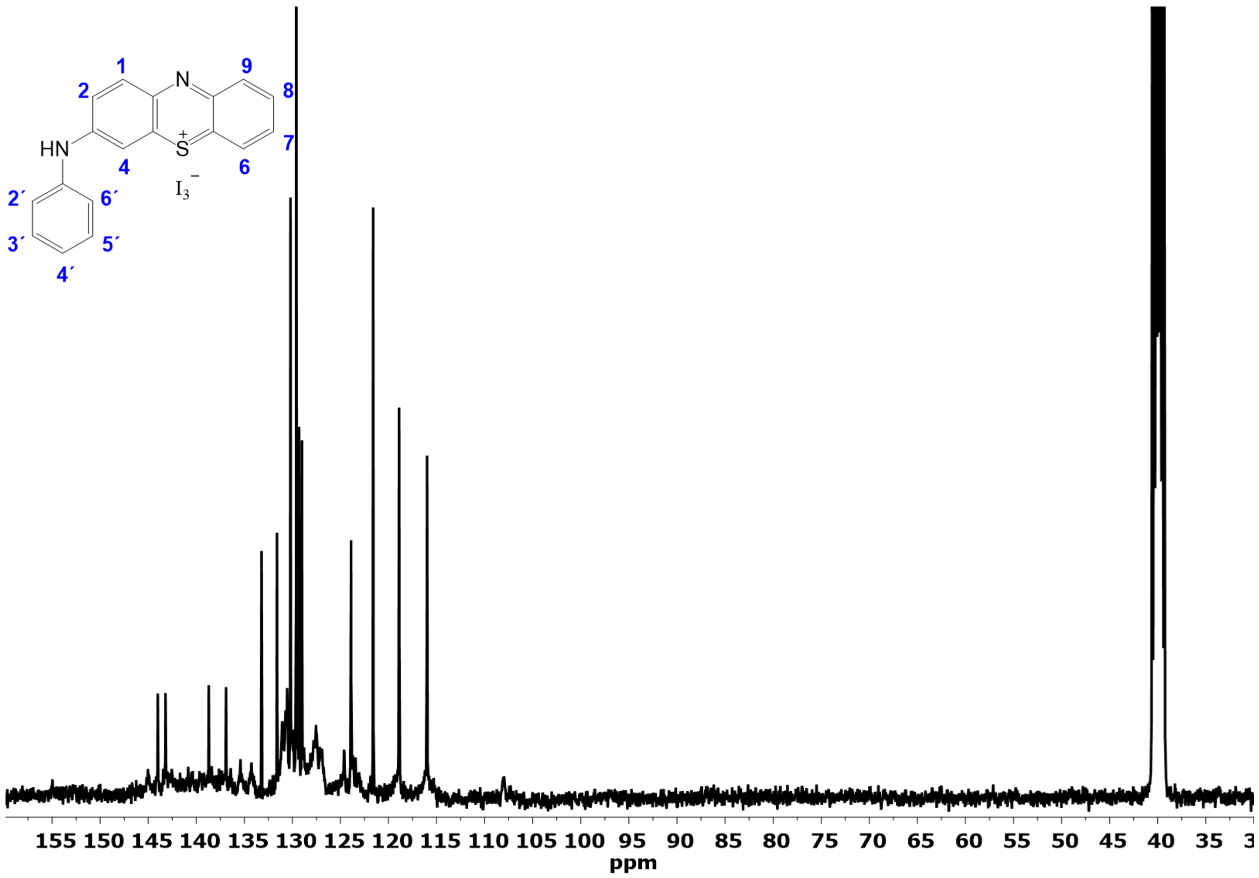


Figure S27. $^{13}$C NMR spectrum of the compound **16**, DMSO-*d*6, 300 K, 100 MHz.

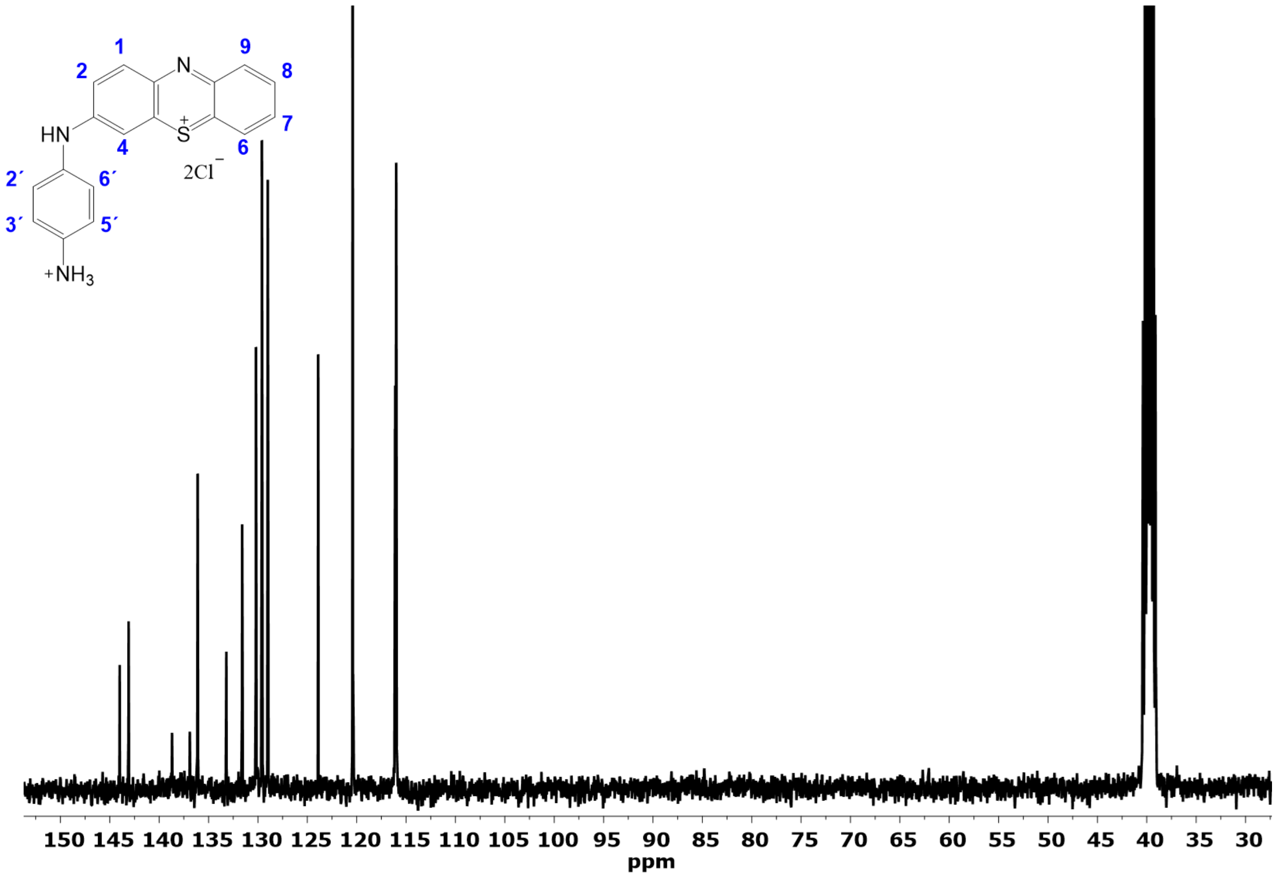


Figure S28. $^{13}$C NMR spectrum of the compound **17**, DMSO-*d*6, 300 K, 100 MHz.

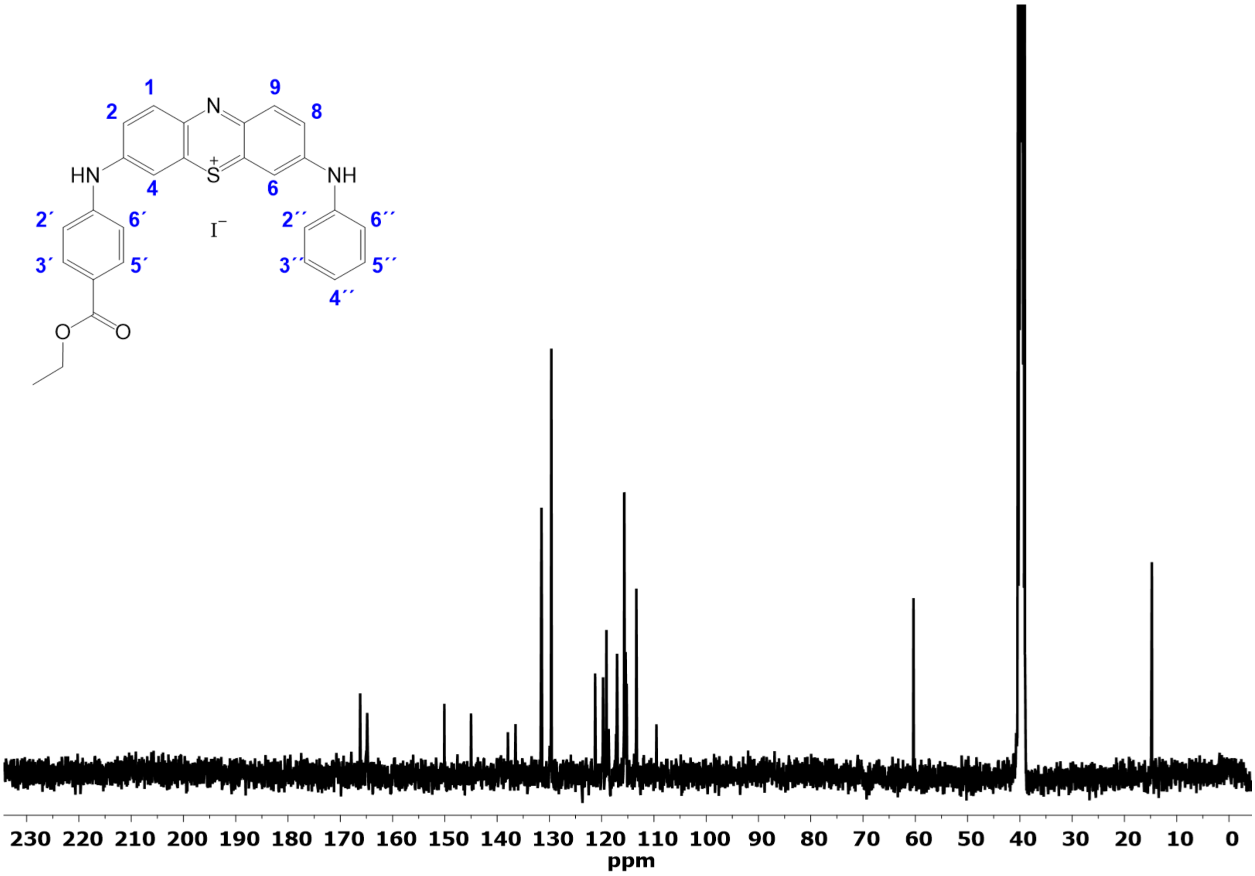


Figure S29. $^{13}$C NMR spectrum of the compound **18**, DMSO-*d*$_6$, 300 K, 100 MHz.

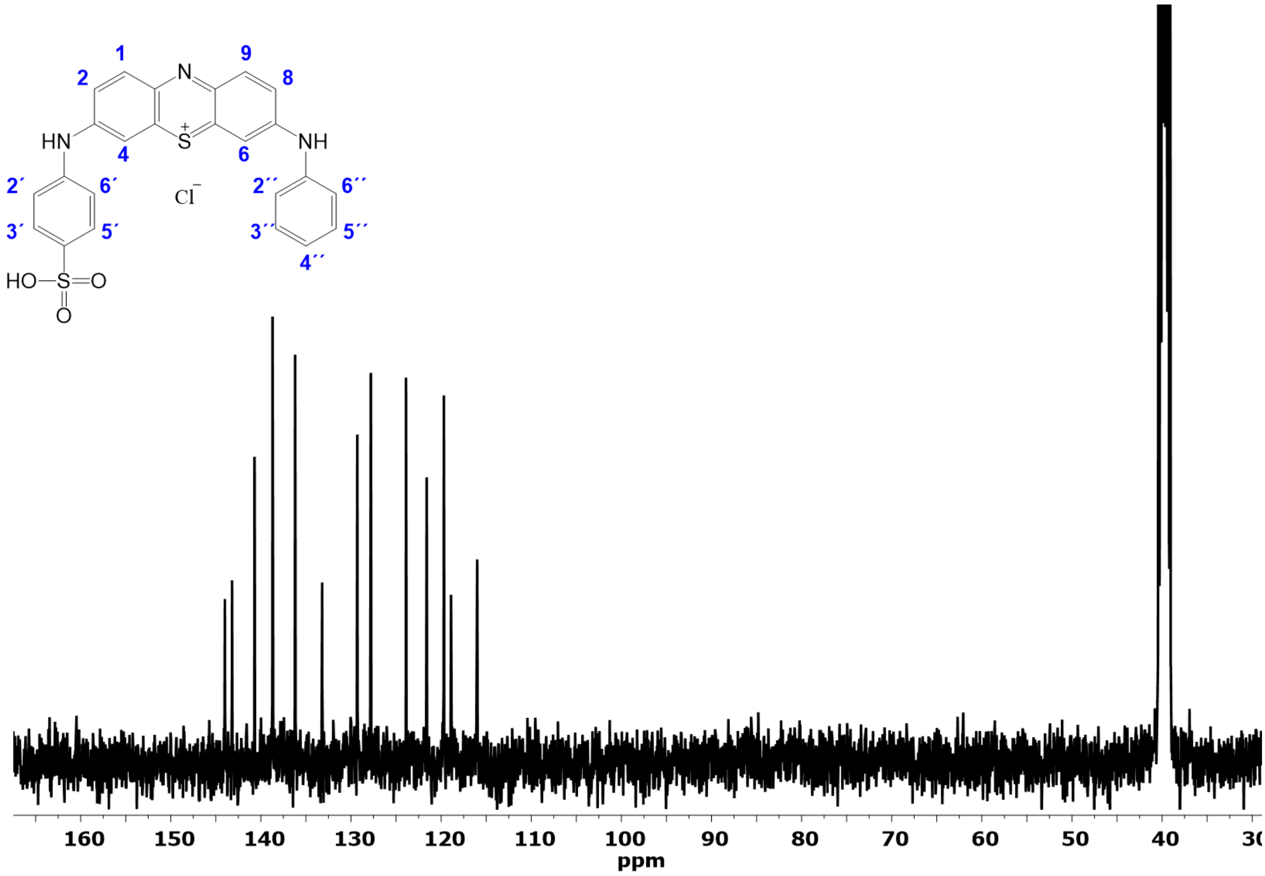


Figure S30. $^{13}$C NMR spectrum of the compound **19**, DMSO-*d*$_6$, 300 K, 100 MHz.

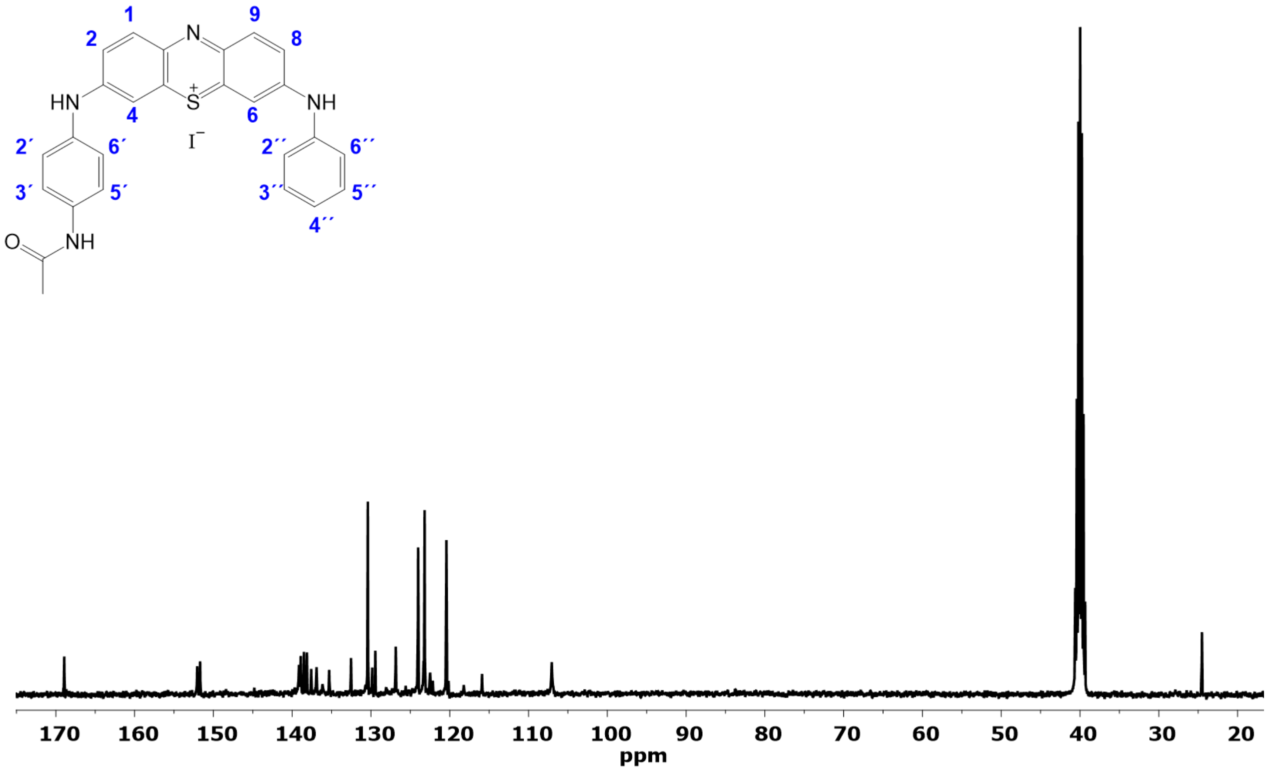


Figure S31. $^{13}C$ NMR spectrum of the compound **20**, DMSO-$d_6$, 300 K, 100 MHz.

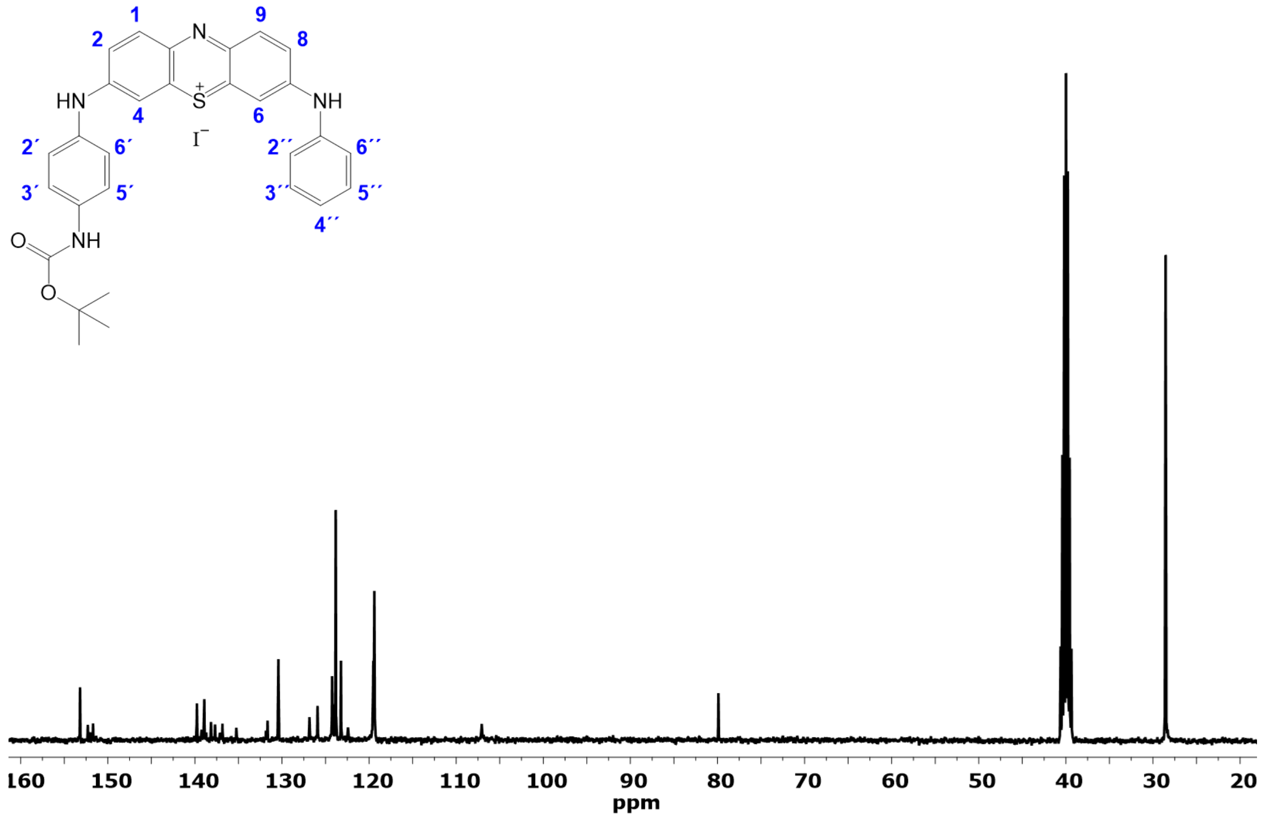


Figure S32. $^{13}C$ NMR spectrum of the compound **21**, DMSO-$d_6$, 300 K, 100 MHz.

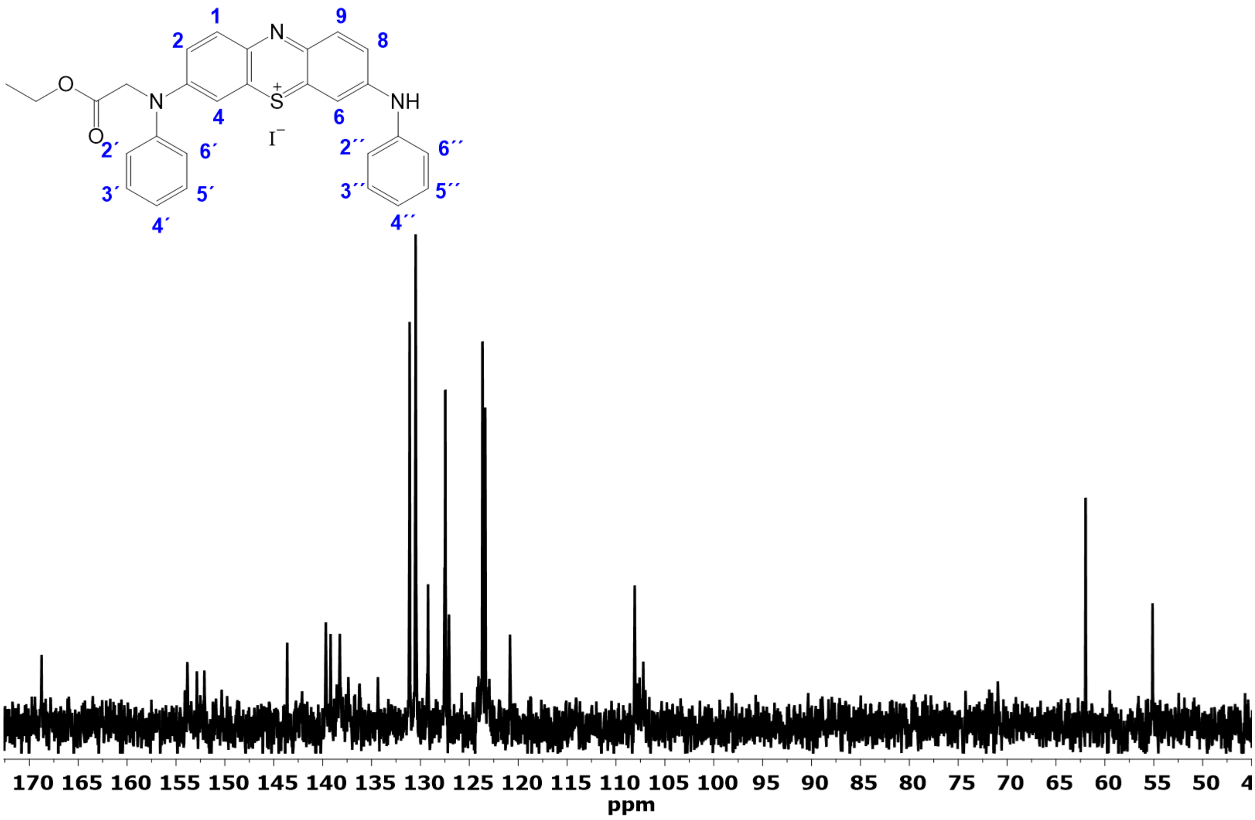


Figure S33. $^{13}$C NMR spectrum of the compound **22**, DMSO-$d_6$, 300 K, 100 MHz.

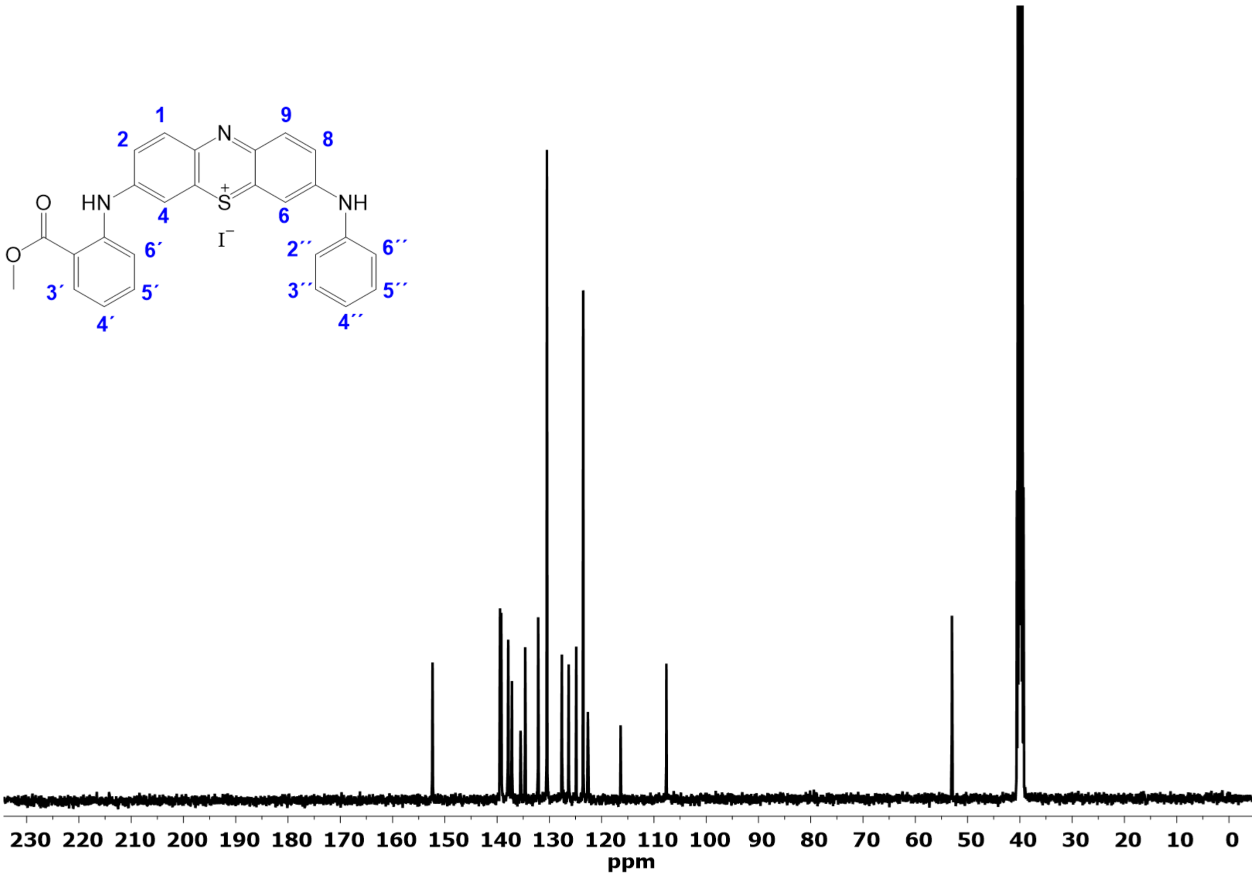


Figure S34. $^{13}$C NMR spectrum of the compound **23**, DMSO-$d_6$, 300 K, 100 MHz.

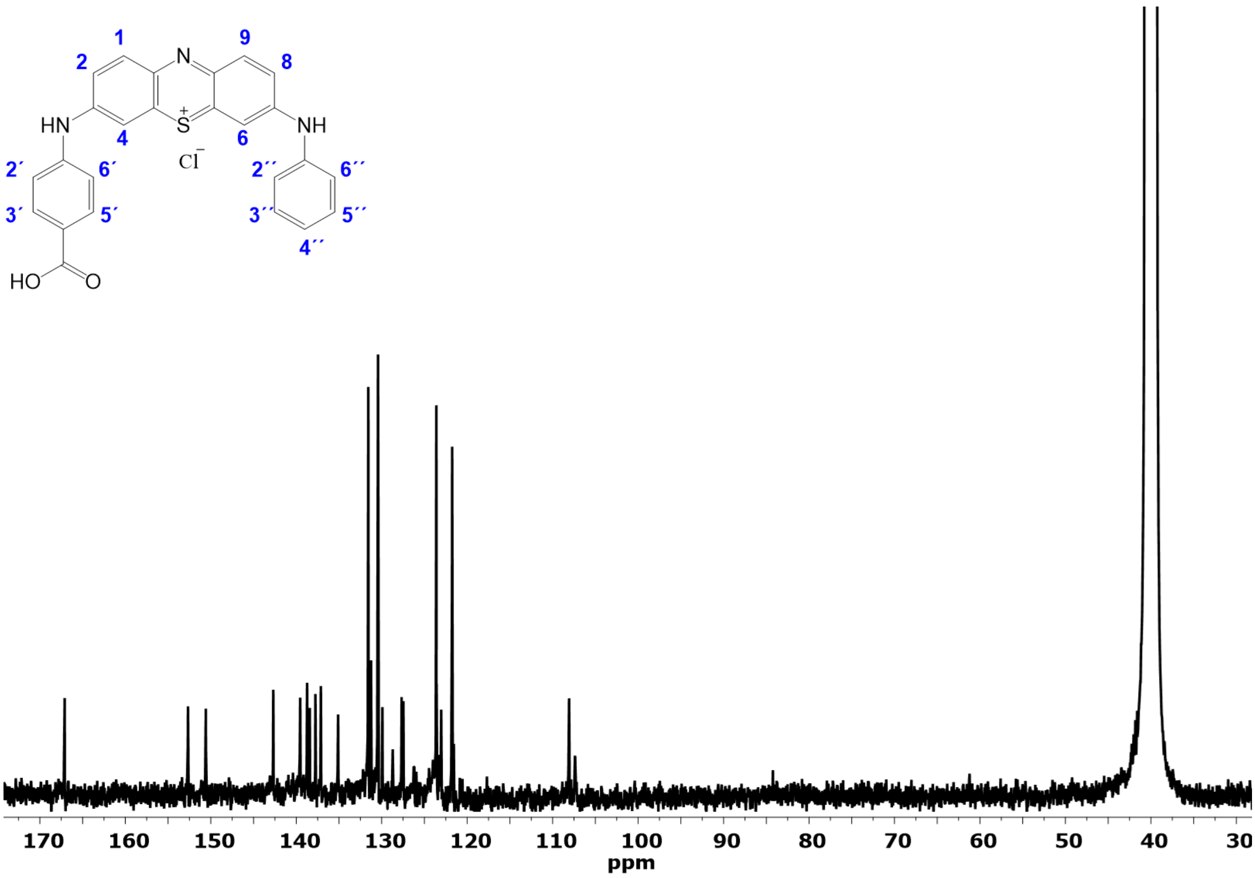


Figure S35. $^{13}C$ NMR spectrum of the compound **24**, DMSO-*d*$_6$, 300 K, 100 MHz.

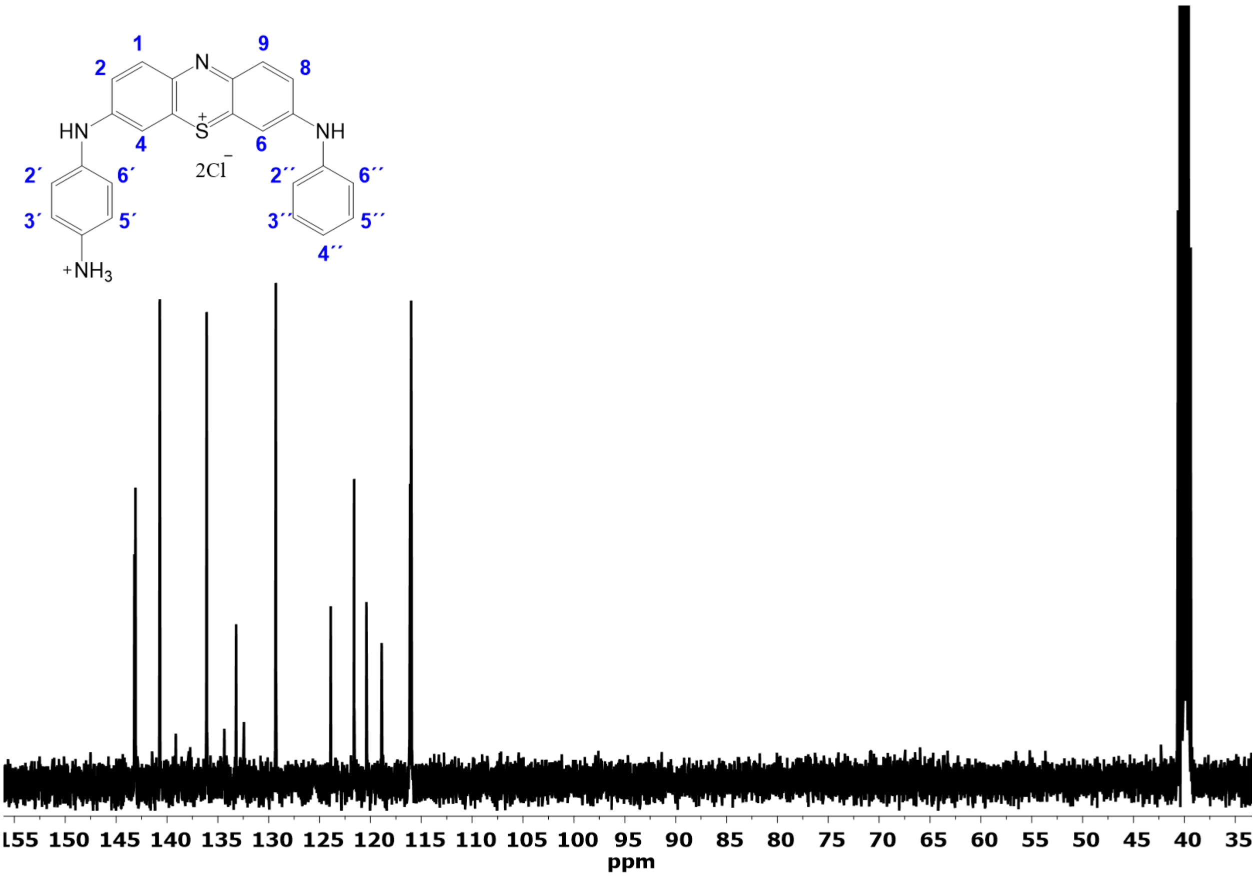


Figure S36. $^{13}C$ NMR spectrum of the compound **25**, DMSO-*d*$_6$, 300 K, 100 MHz.

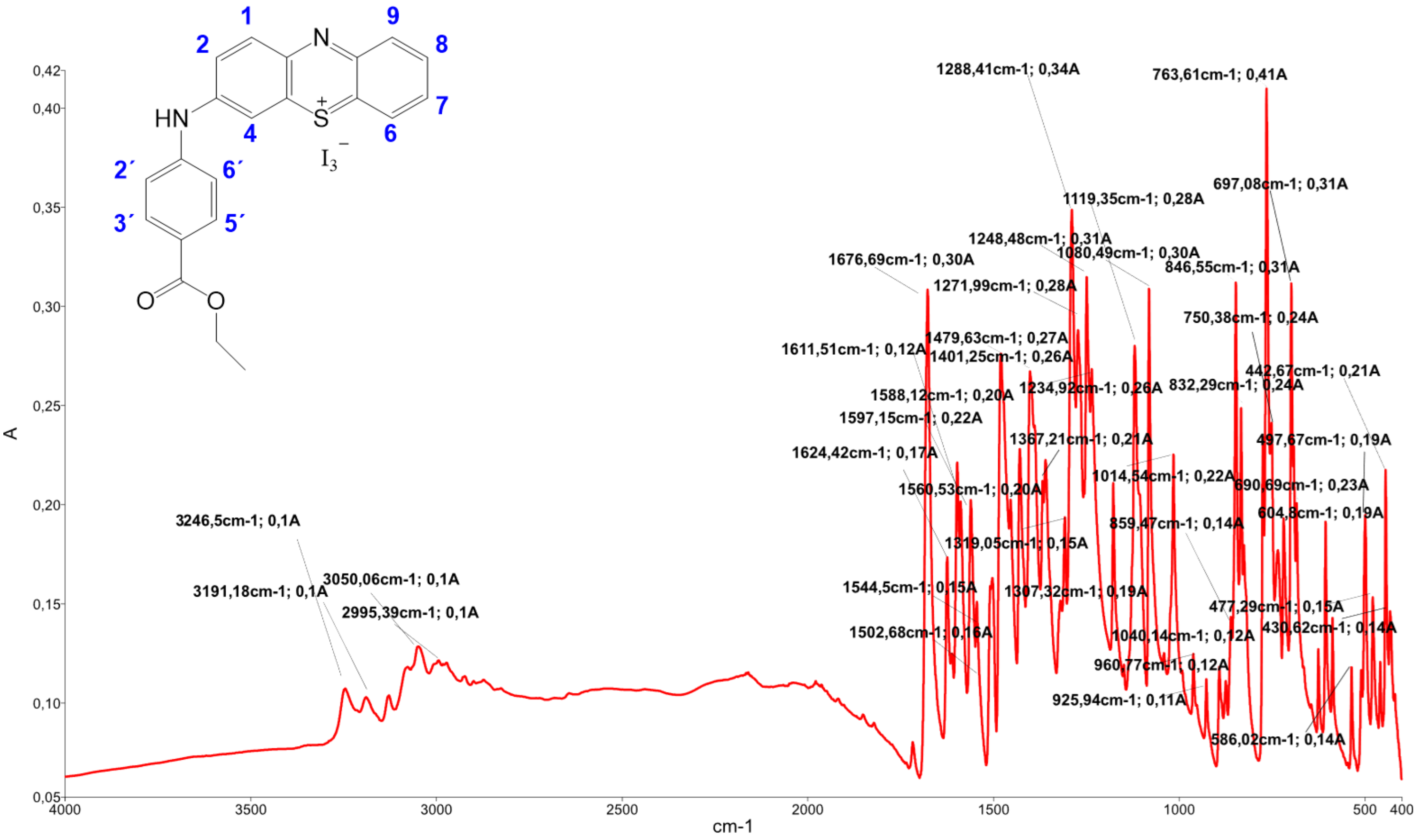

Figure S37. FT-IR spectrum of the compound **10**.

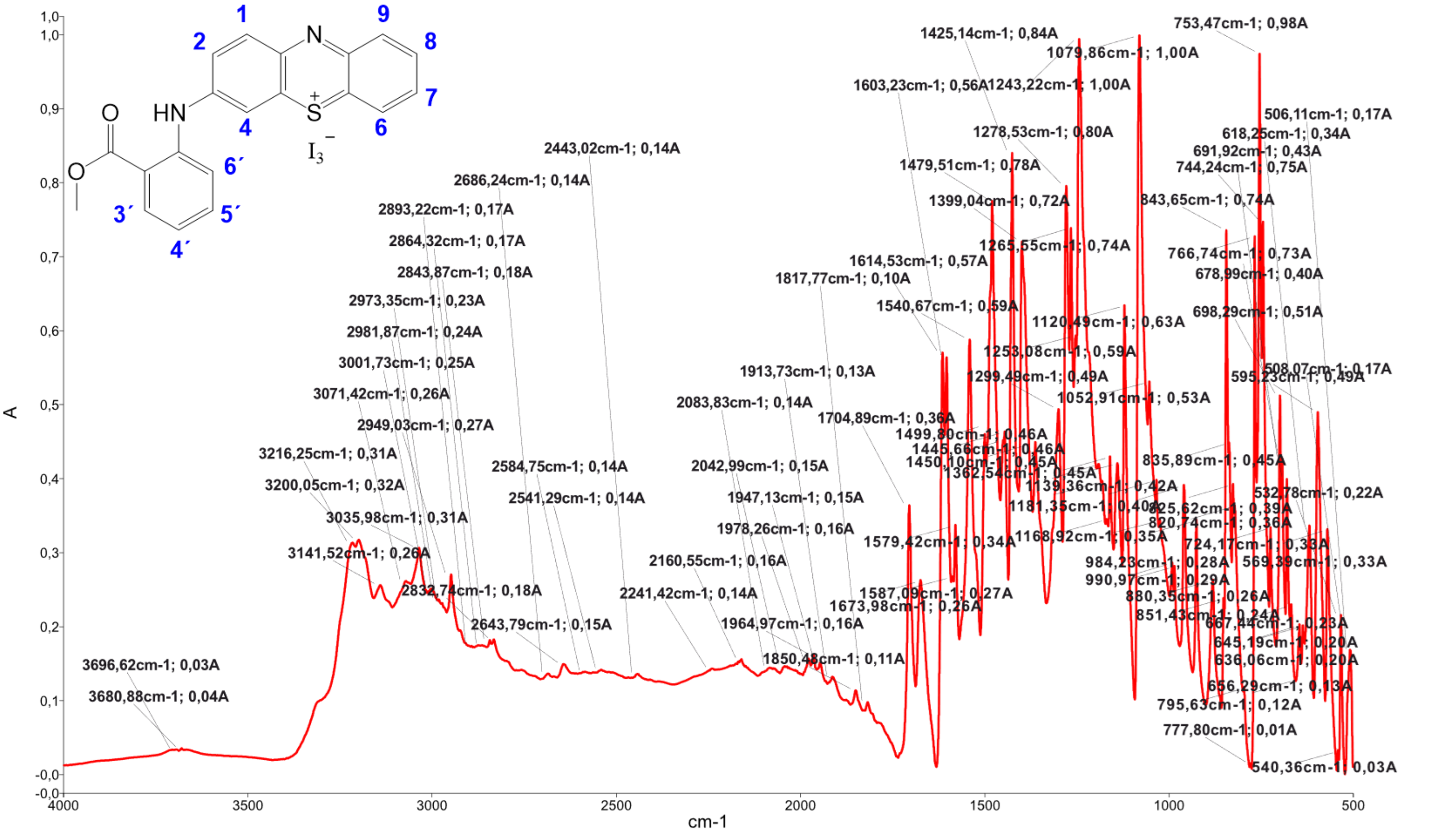

Figure S38. FT-IR spectrum of the compound **11**.

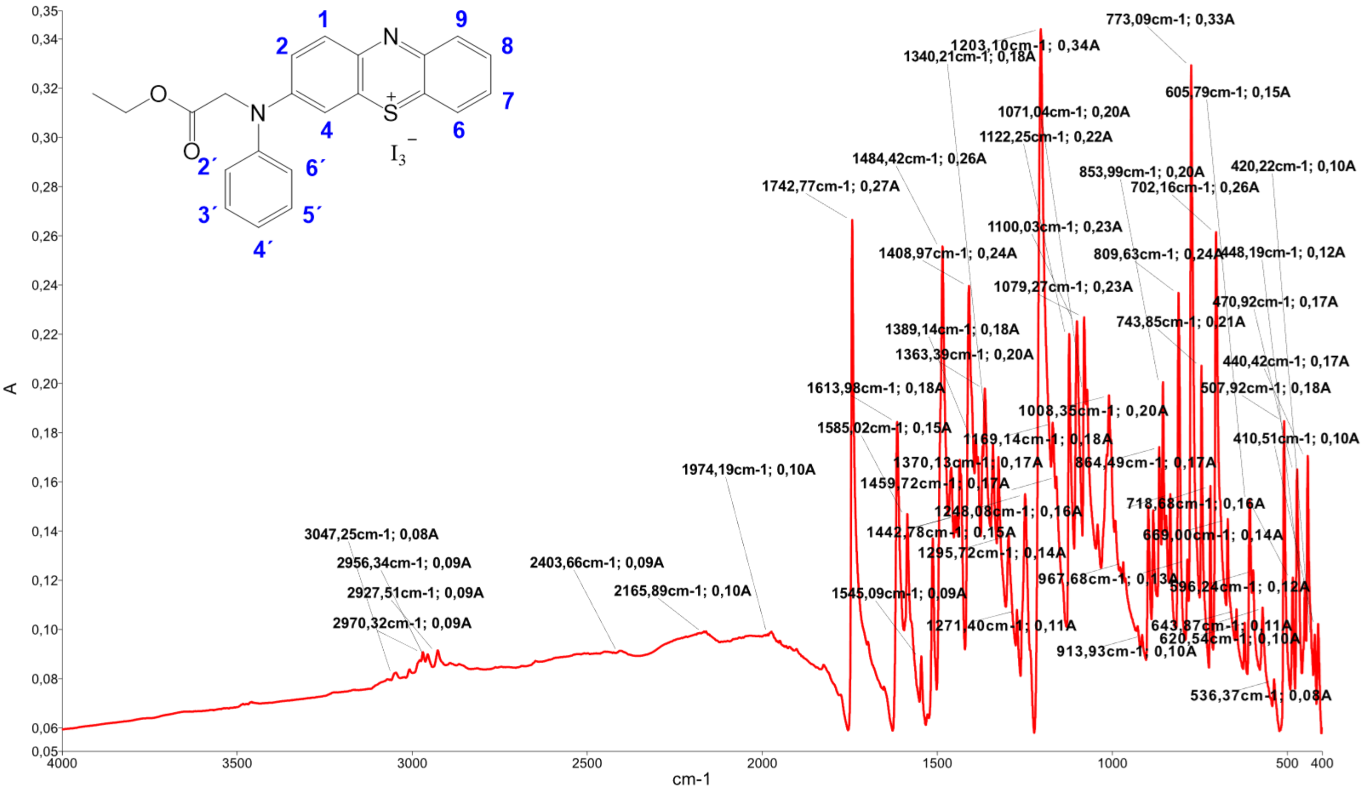


Figure S39. FT-IR spectrum of the compound **12**.

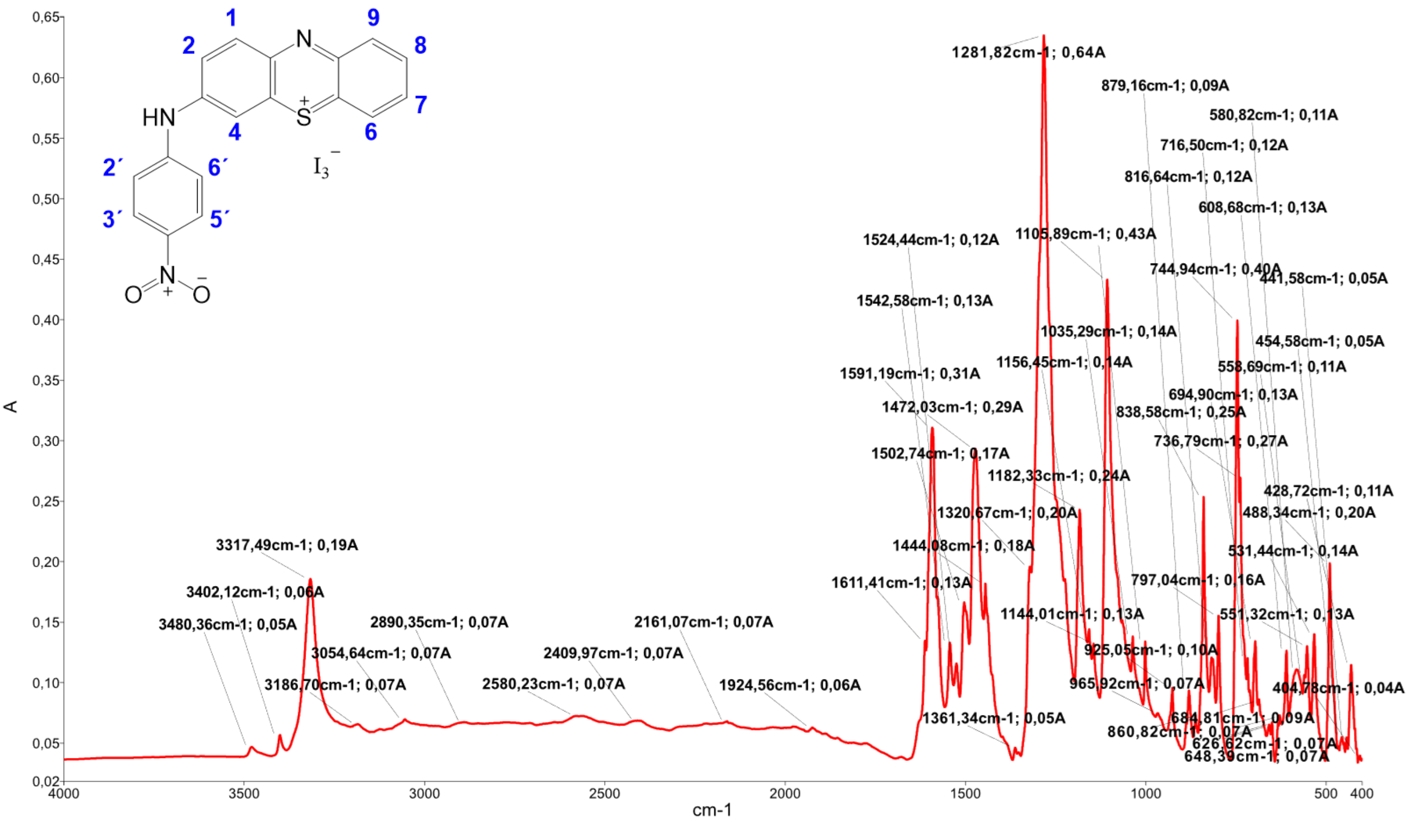


Figure S40. FT-IR spectrum of the compound **13**.

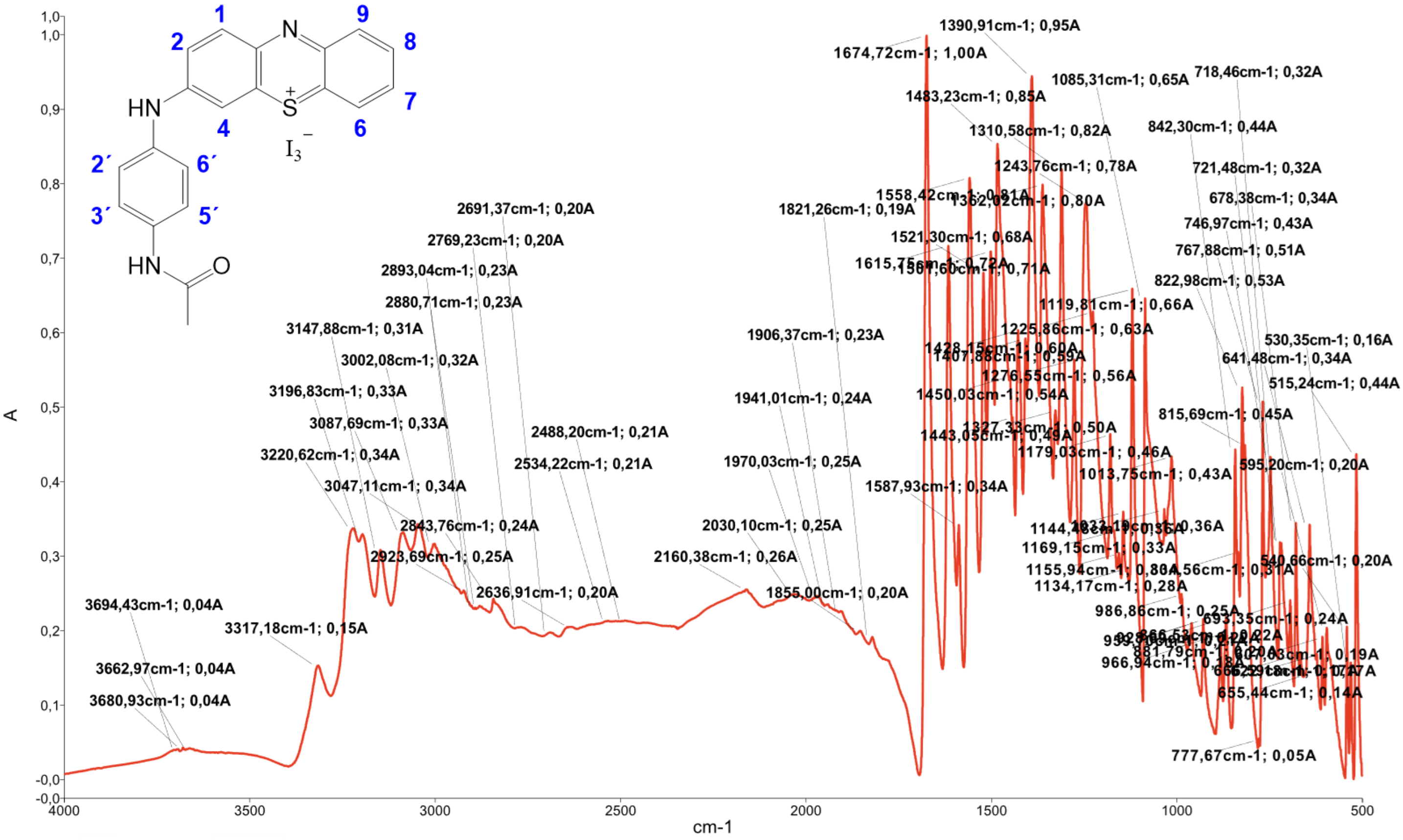


Figure S41. FT-IR spectrum of the compound **14**.

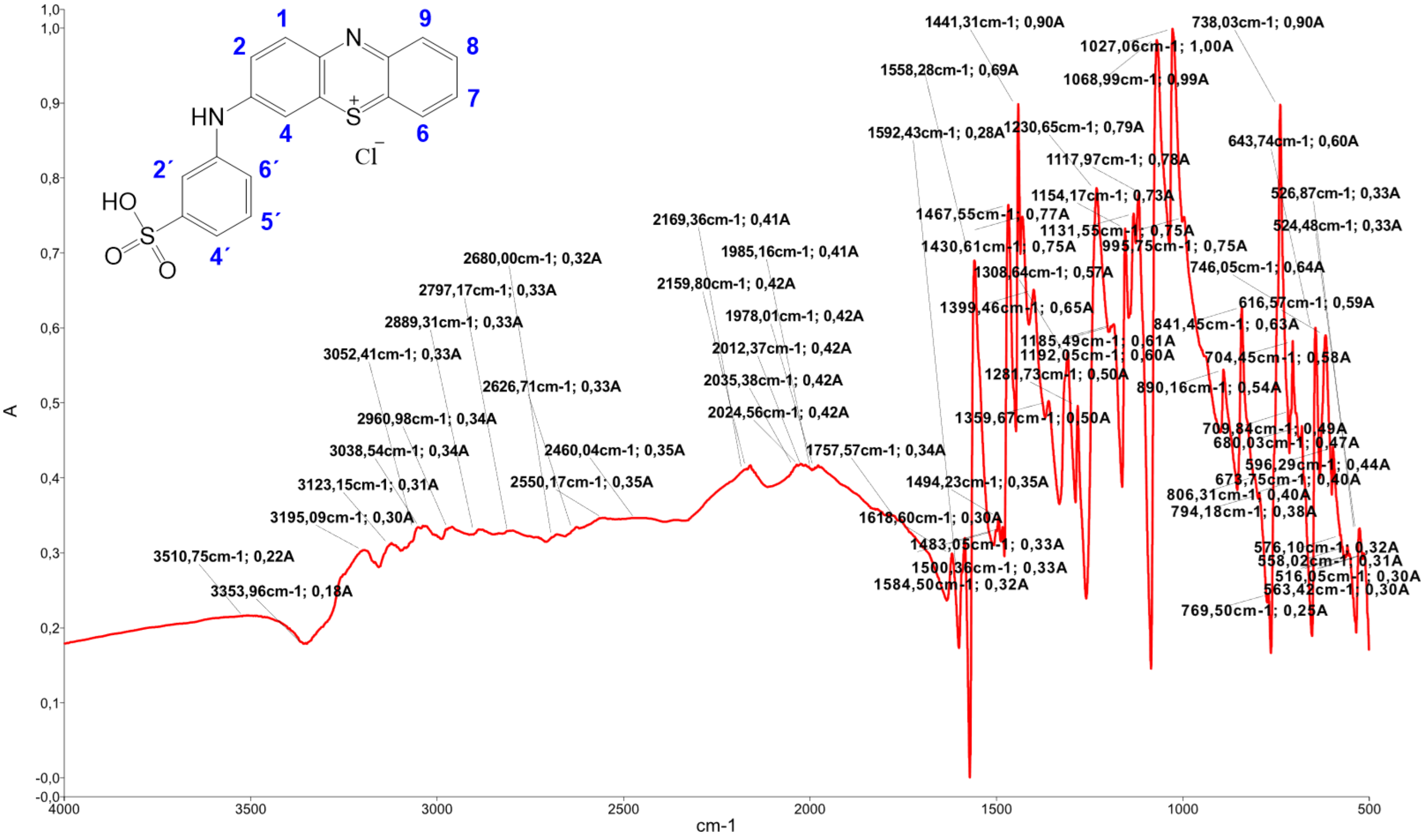


Figure S42. FT-IR spectrum of the compound **15**.

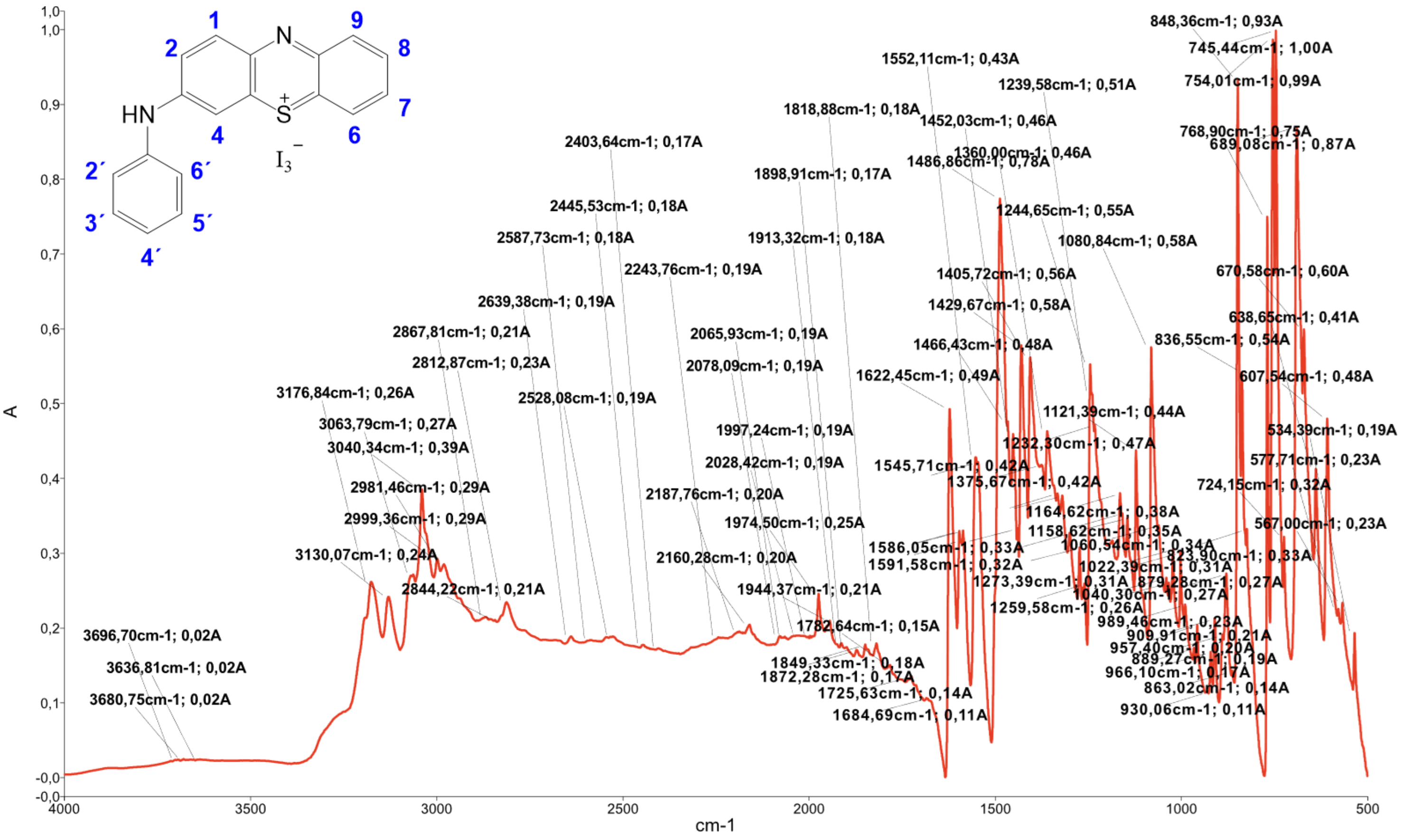

Figure S43. FT-IR spectrum of the compound **16**.

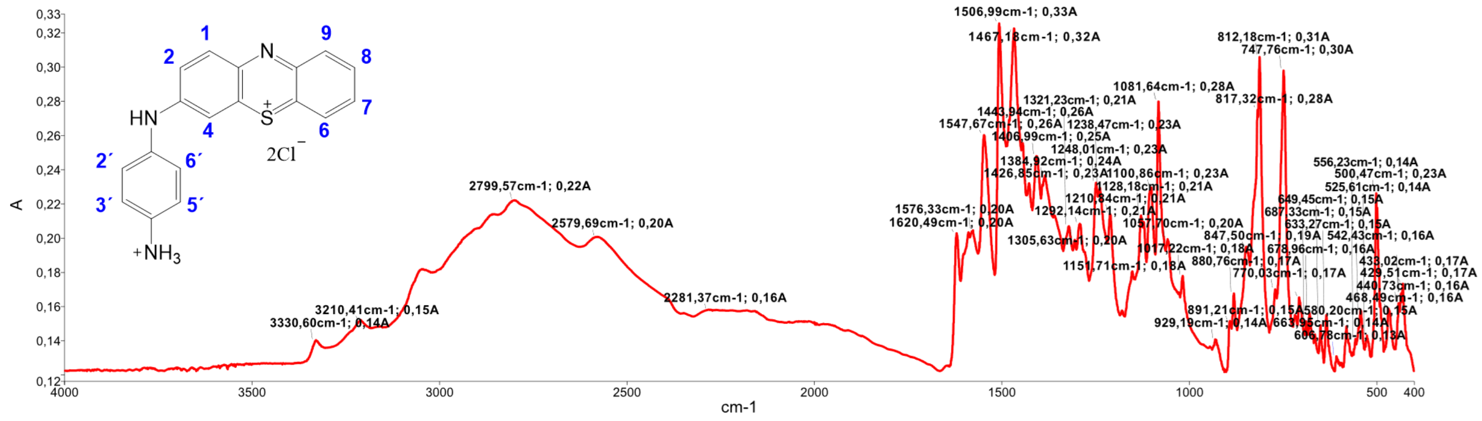

Figure S44. FT-IR spectrum of the compound **17**.

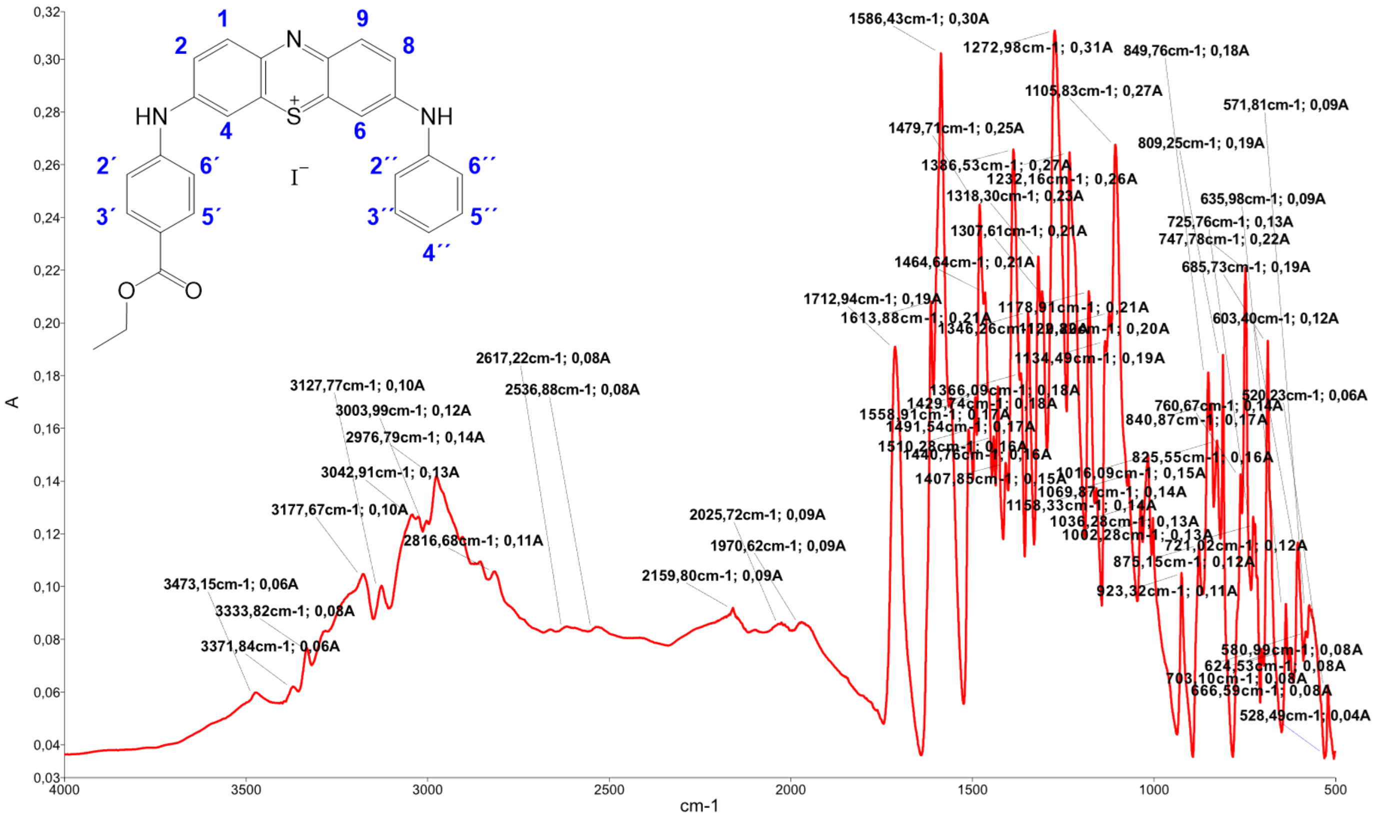


Figure S45. FT-IR spectrum of the compound **18**.

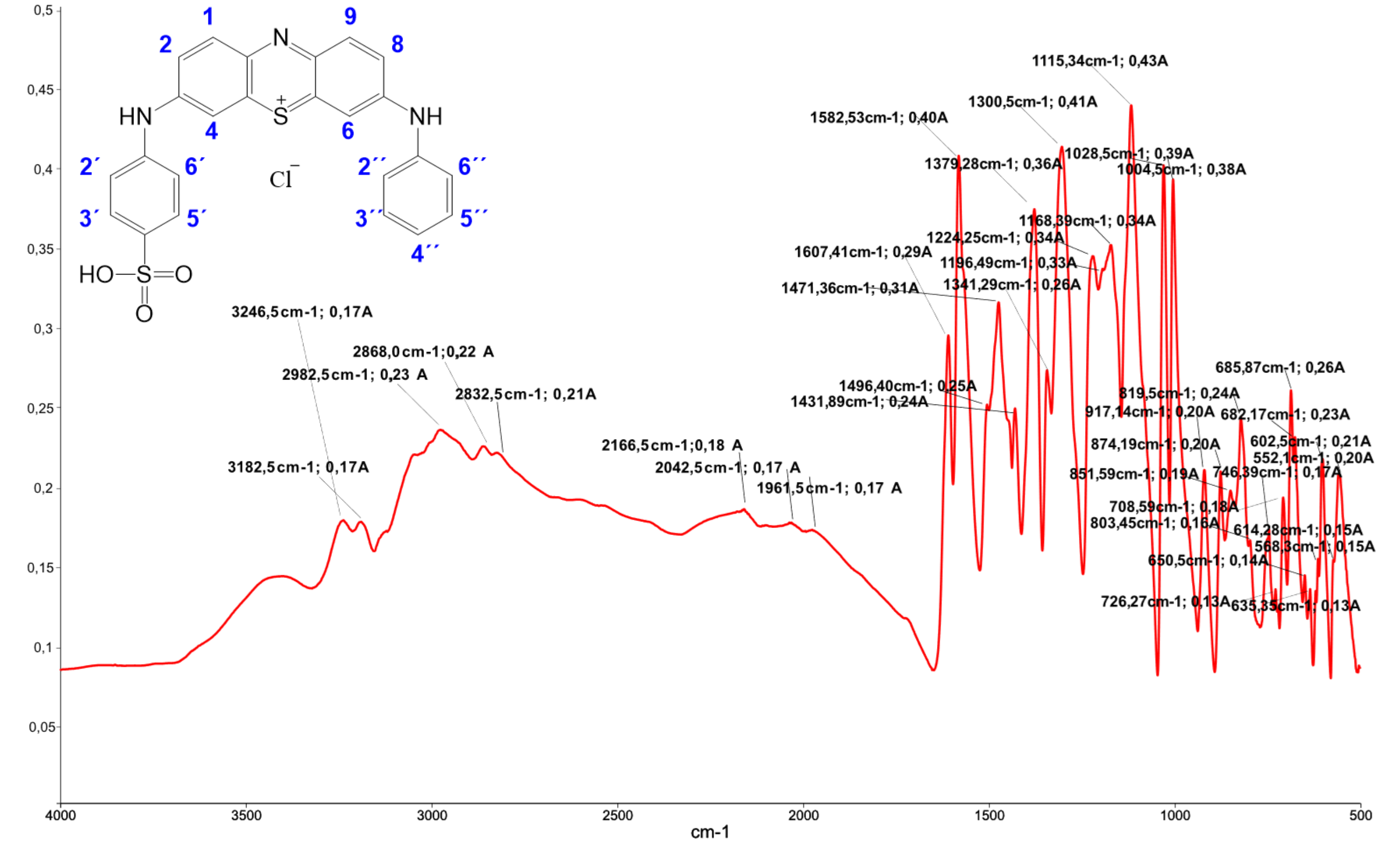


Figure S46. FT-IR spectrum of the compound **19**.

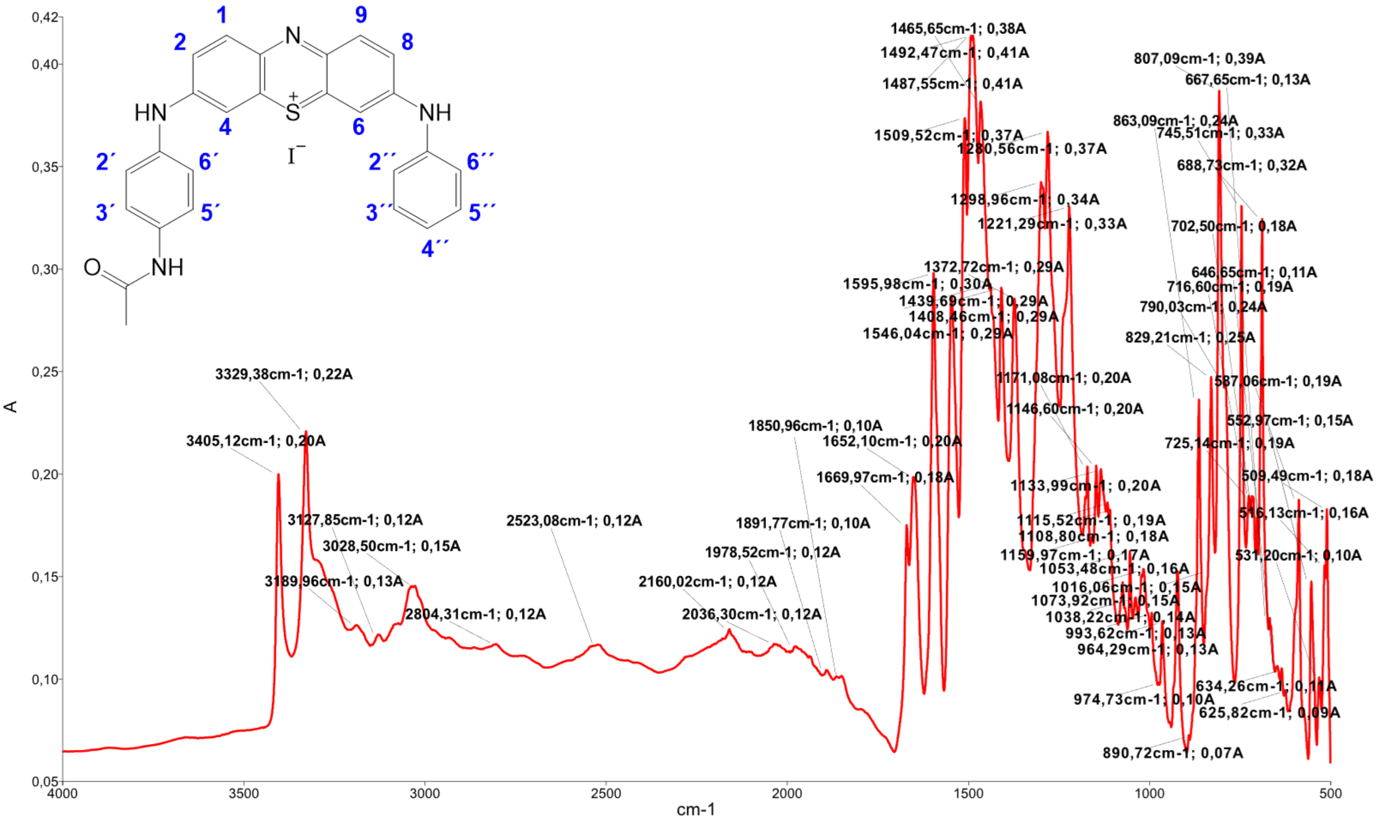

Figure S47. FT-IR spectrum of the compound **20**.

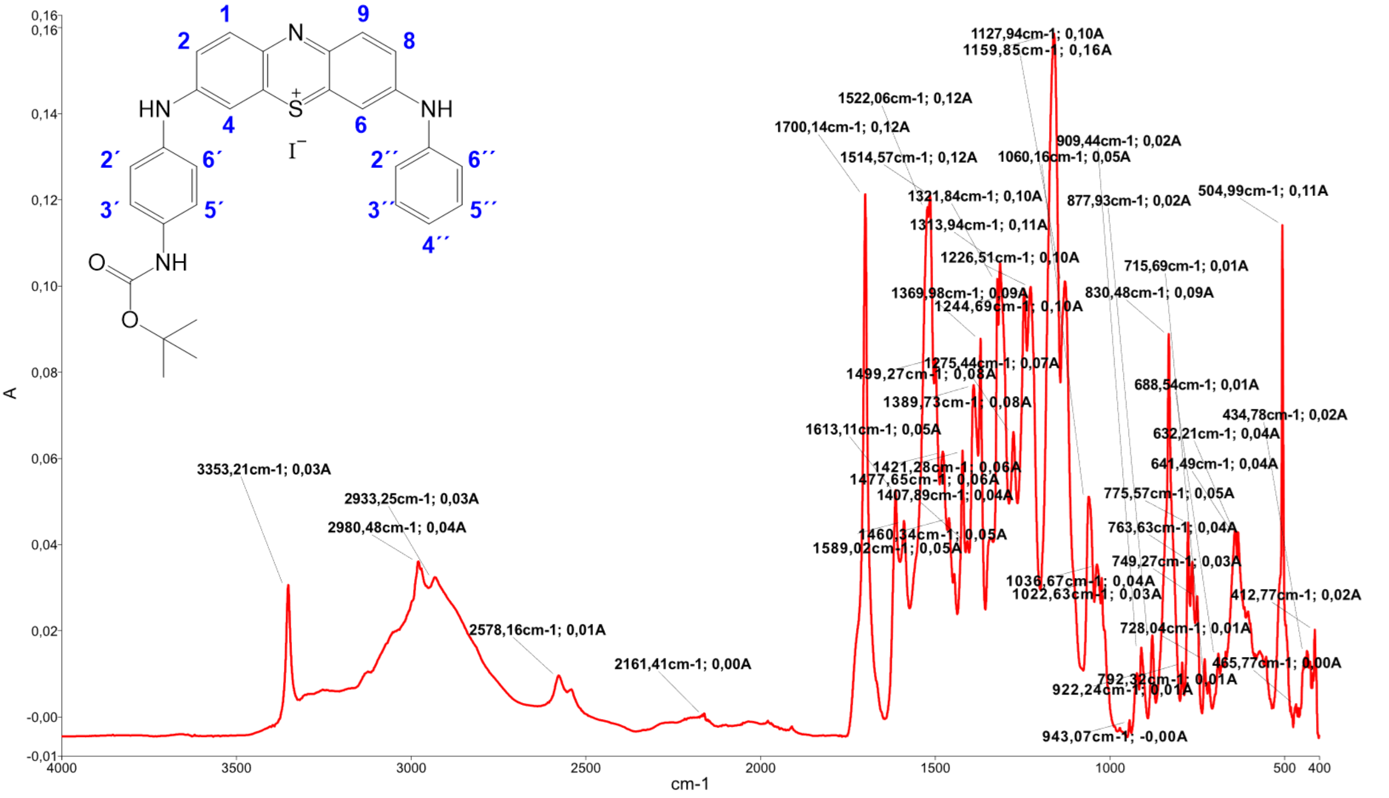

Figure S48. FT-IR spectrum of the compound **21**.

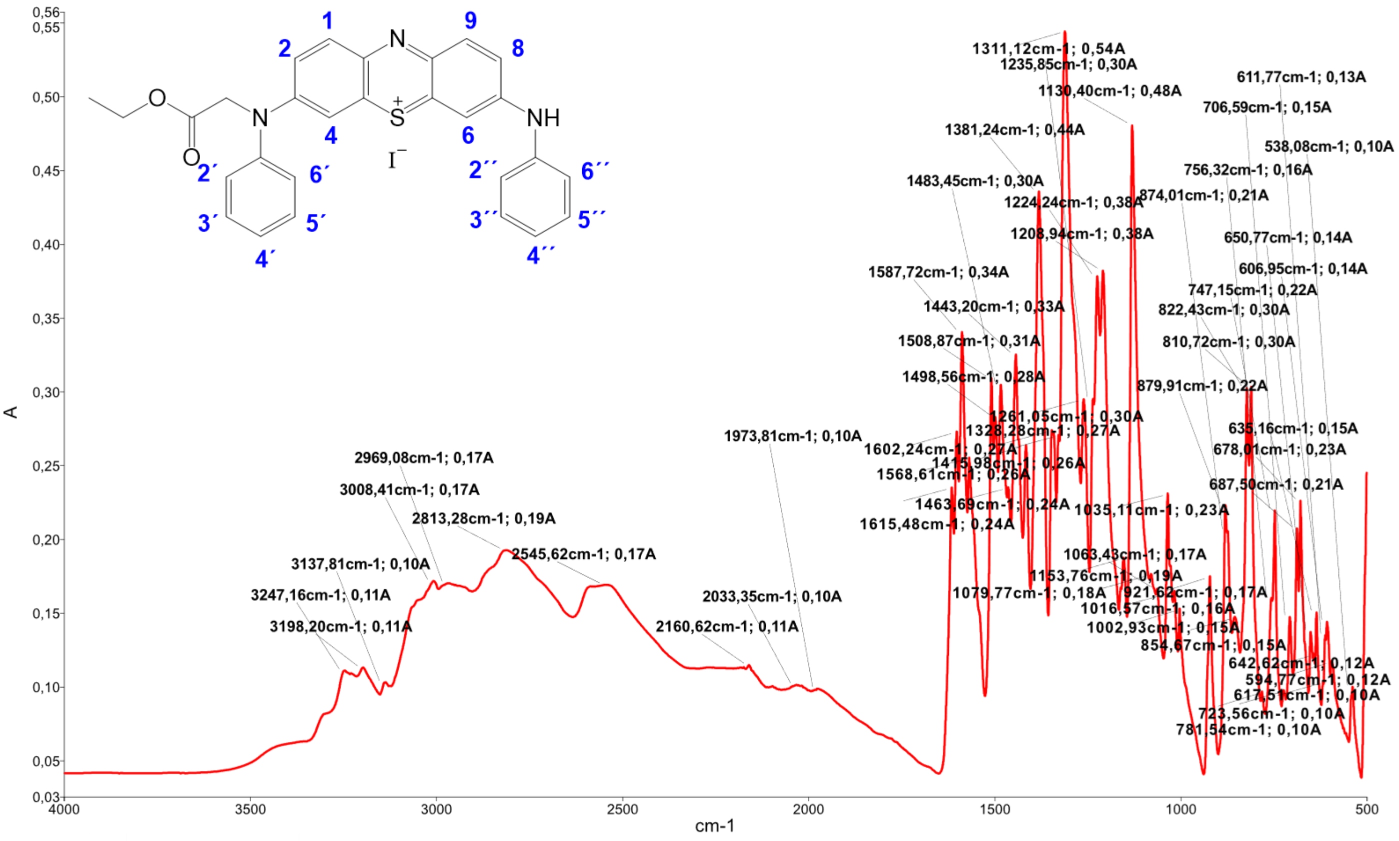


Figure S49. FT-IR spectrum of the compound **22**.

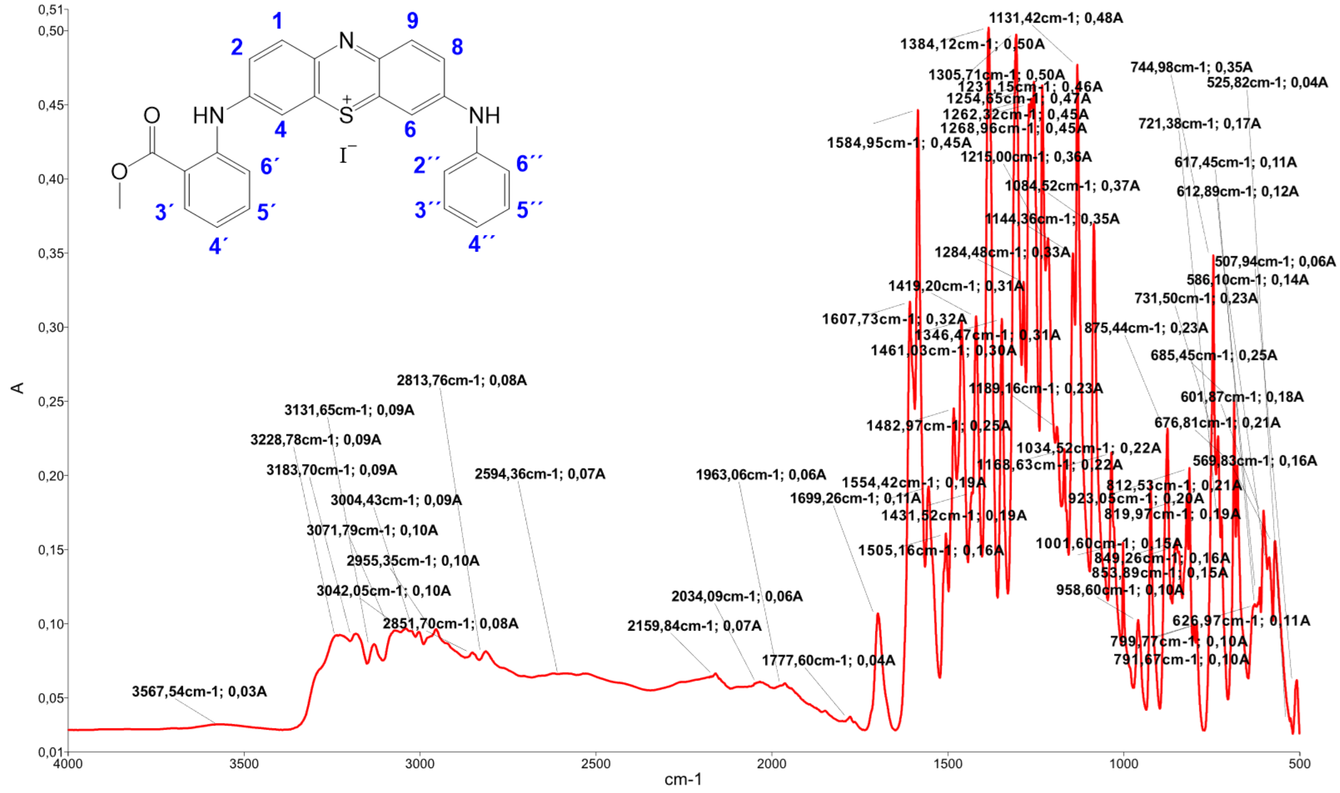


Figure S50. FT-IR spectrum of the compound **23**.

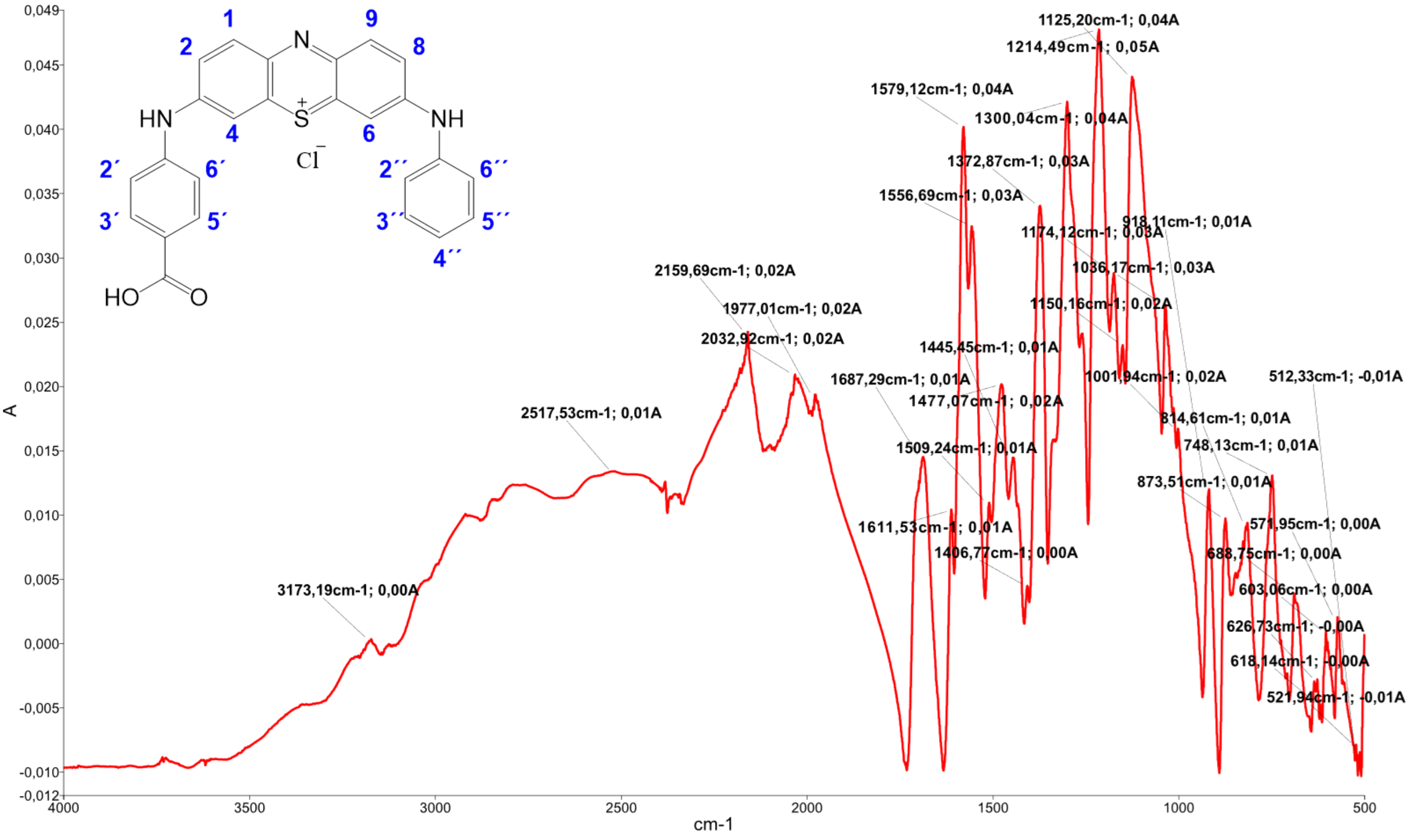


Figure S51. FT-IR spectrum of the compound **24**.

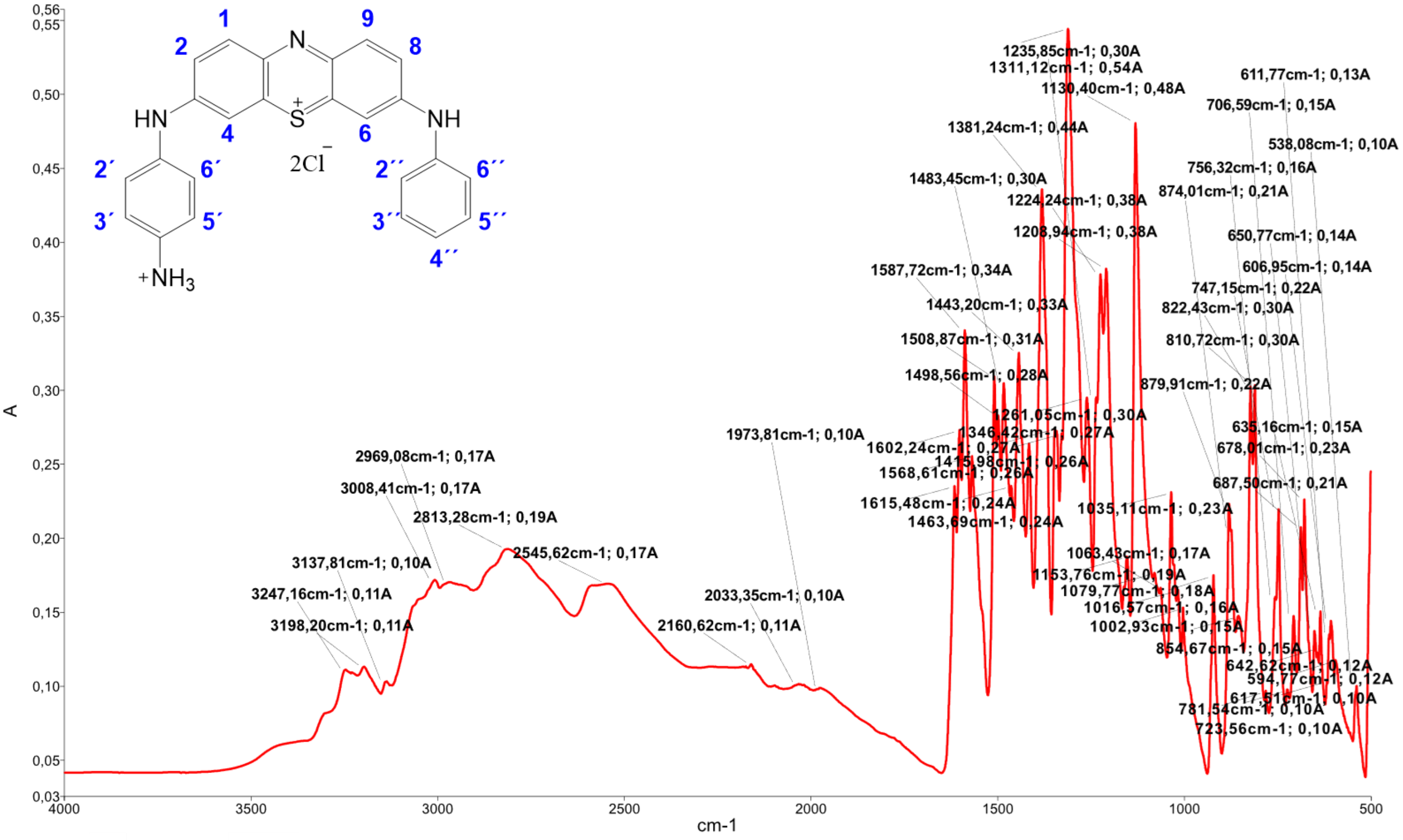


Figure S52. FT-IR spectrum of the compound **25**.

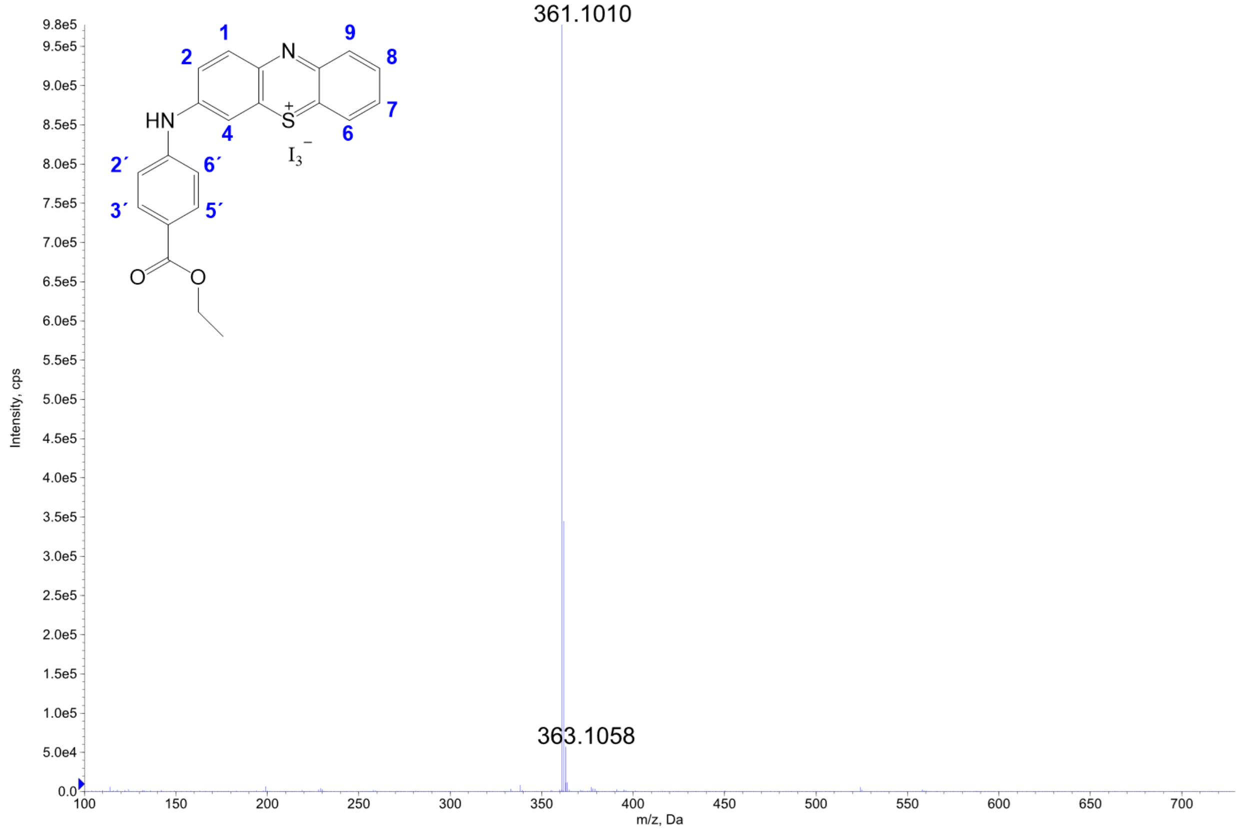


Figure S53. HRMS spectrum of the compound **10**.

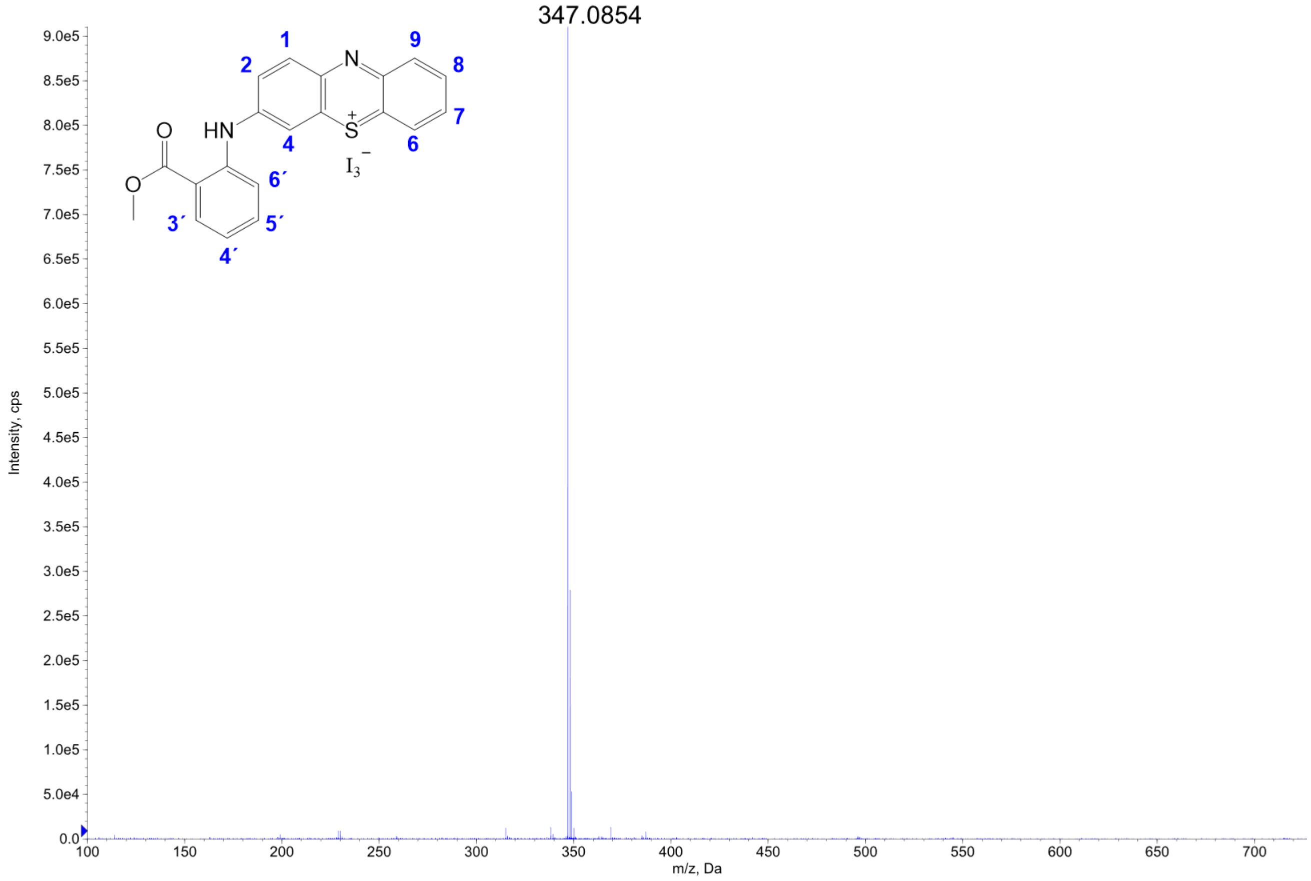


Figure S54. HRMS spectrum of the compound **11**.

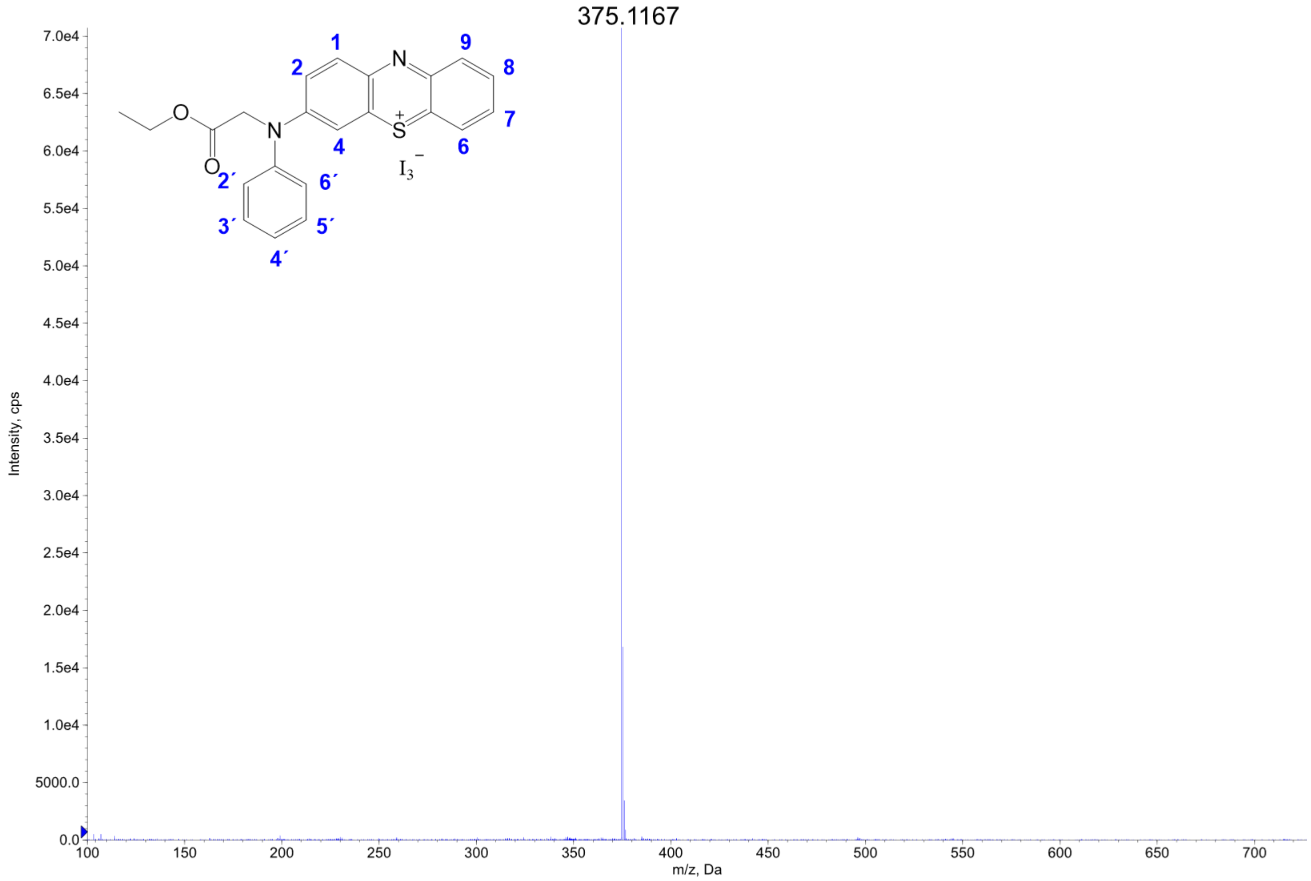


Figure S55. HRMS spectrum of the compound **12**.

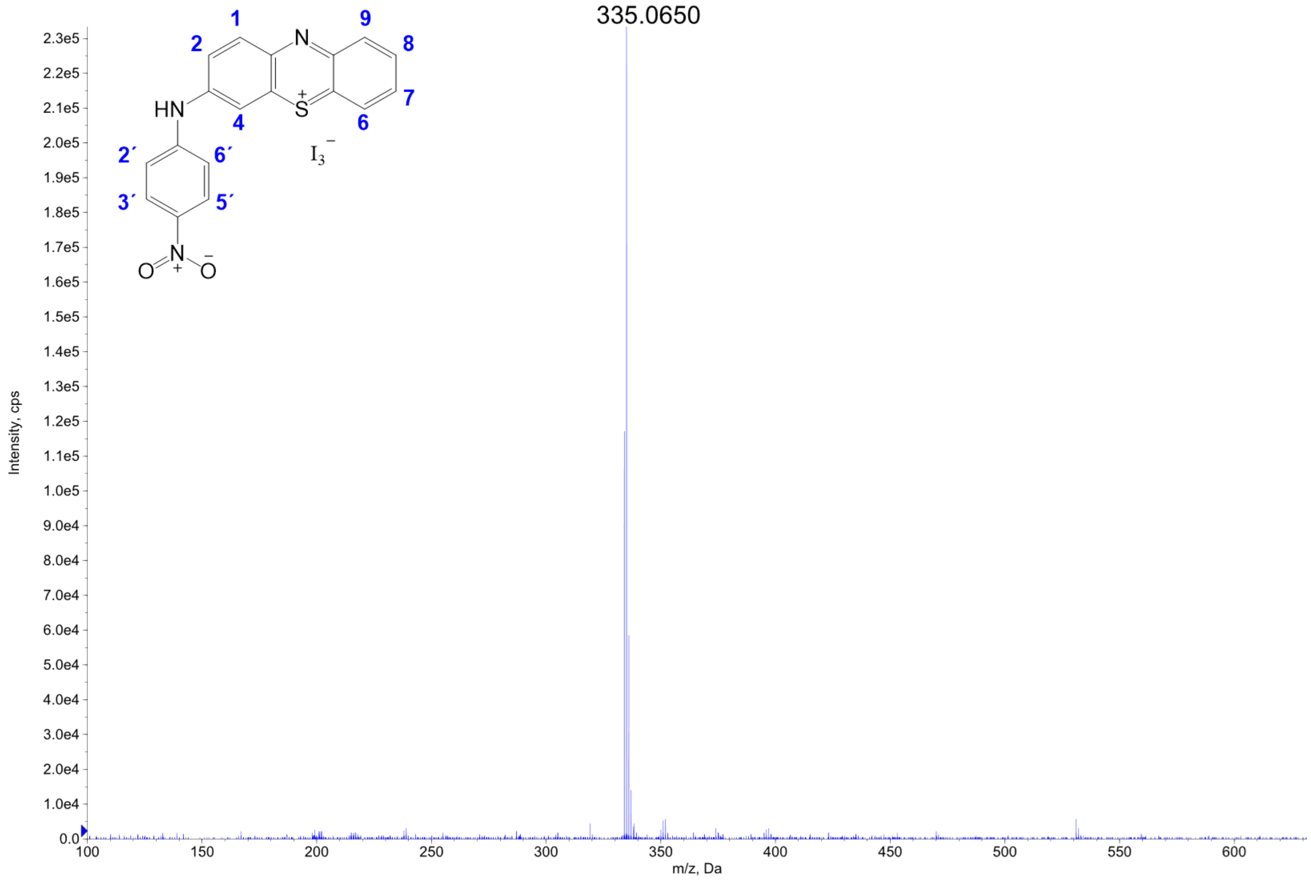


Figure S56. HRMS spectrum of the compound **13**.

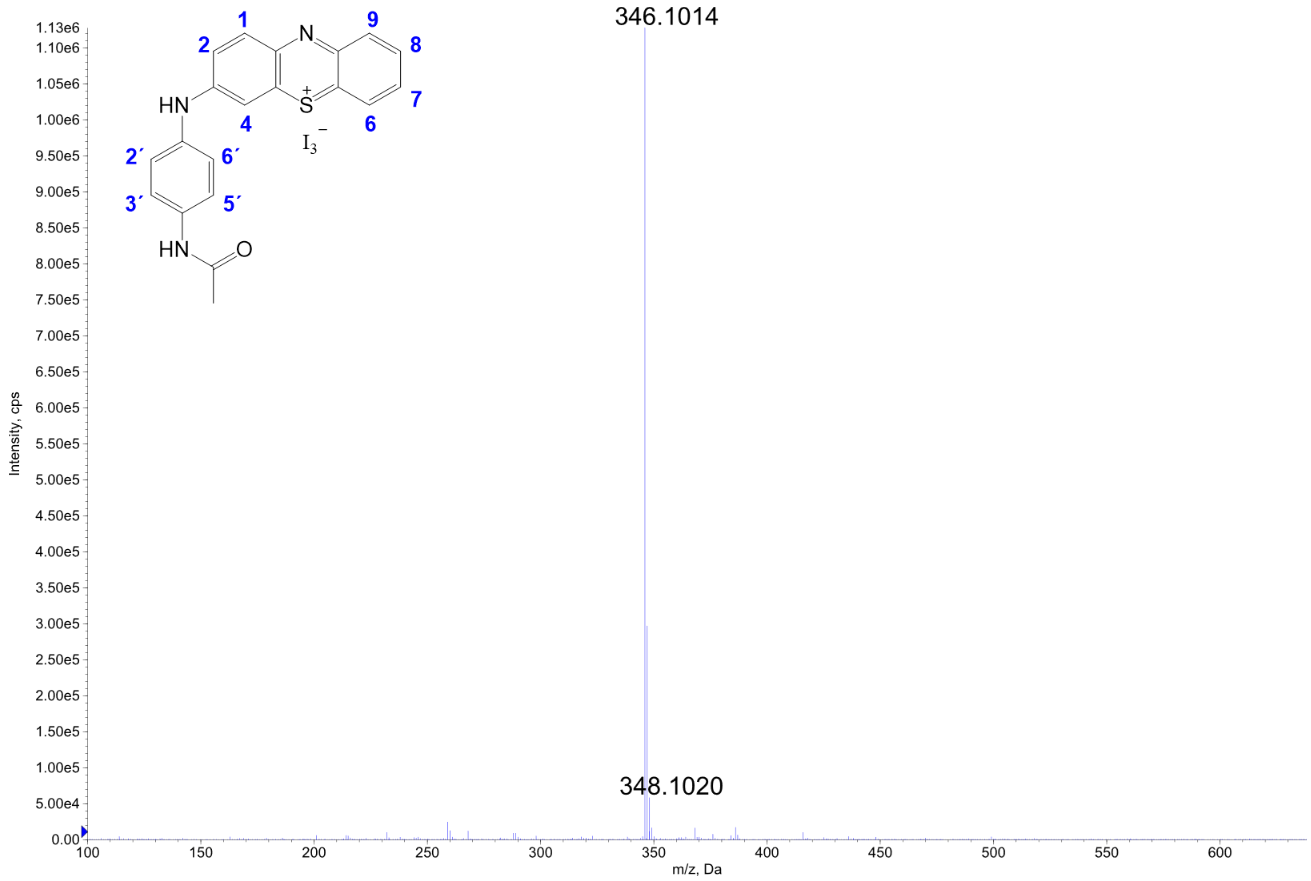


Figure S57. HRMS spectrum of the compound **14**.

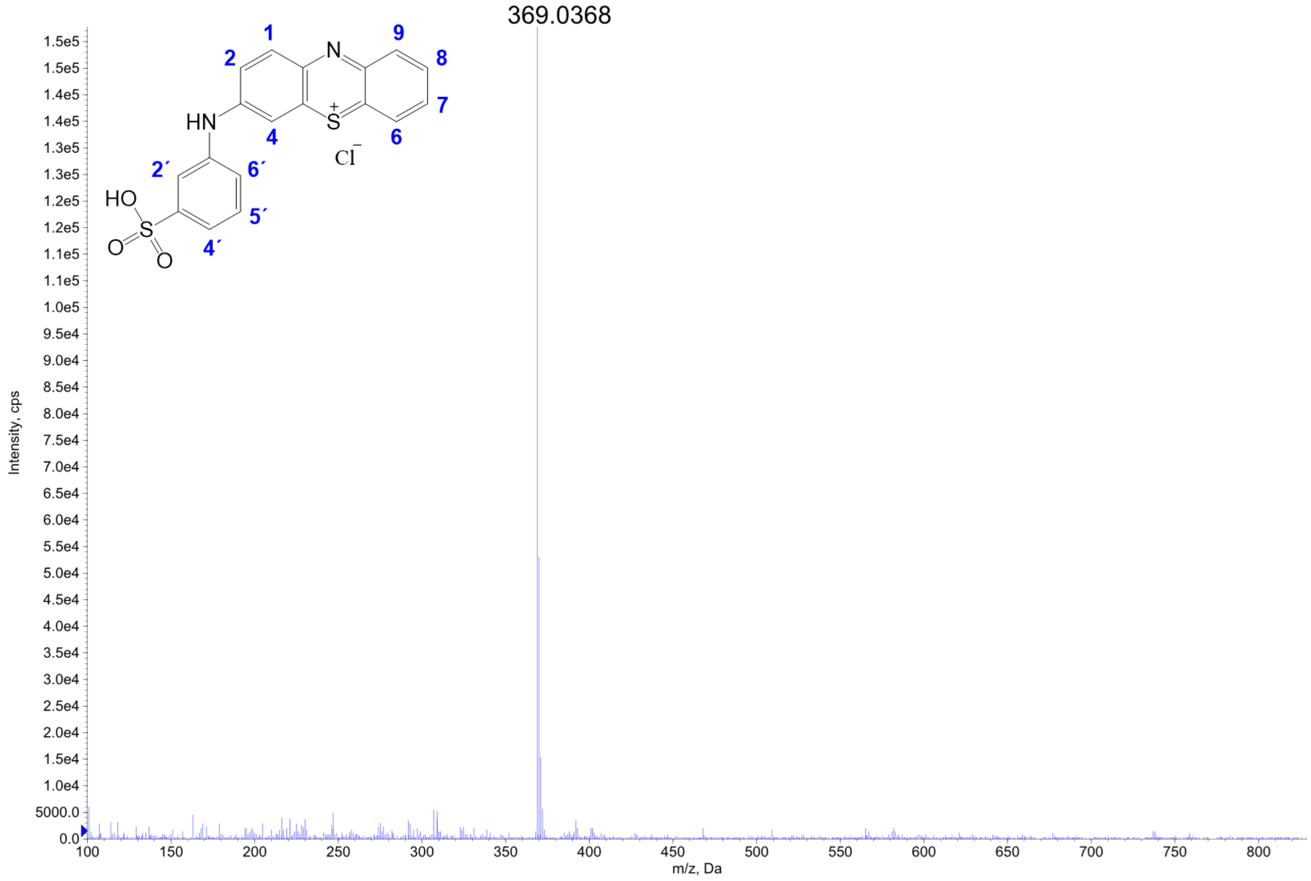


Figure S58. HRMS spectrum of the compound **15**.

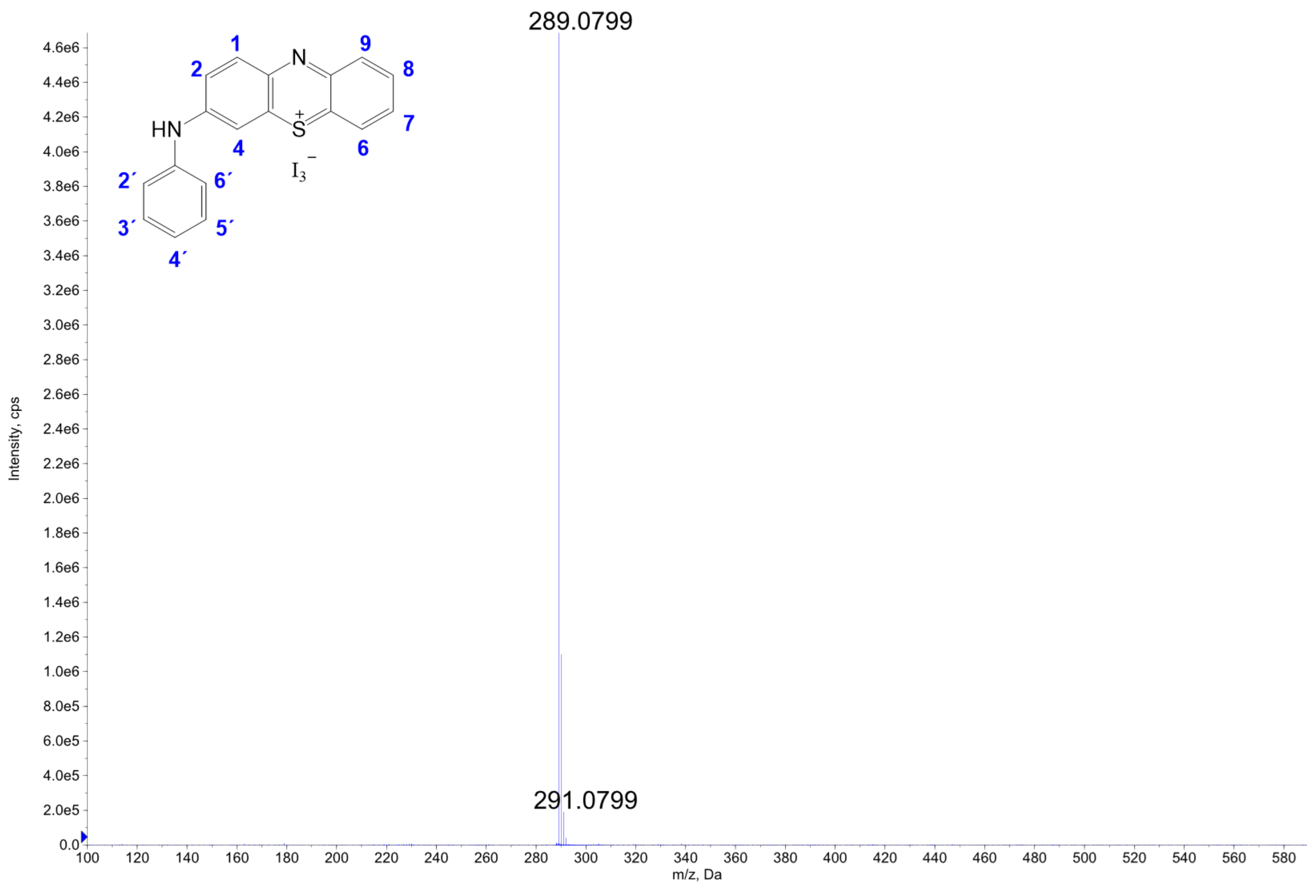


Figure S59. HRMS spectrum of the compound **16**.

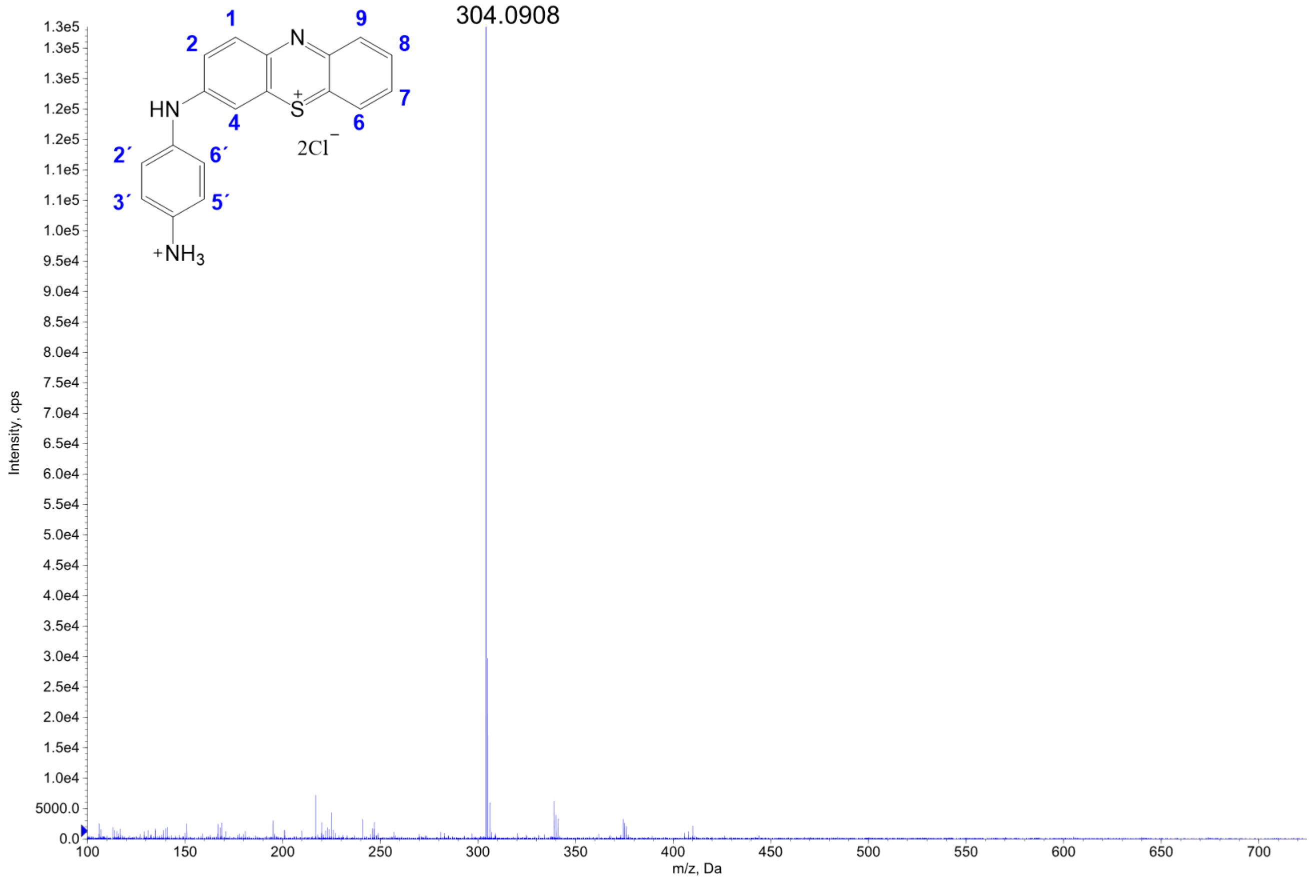


Figure S60. HRMS spectrum of the compound **17**.

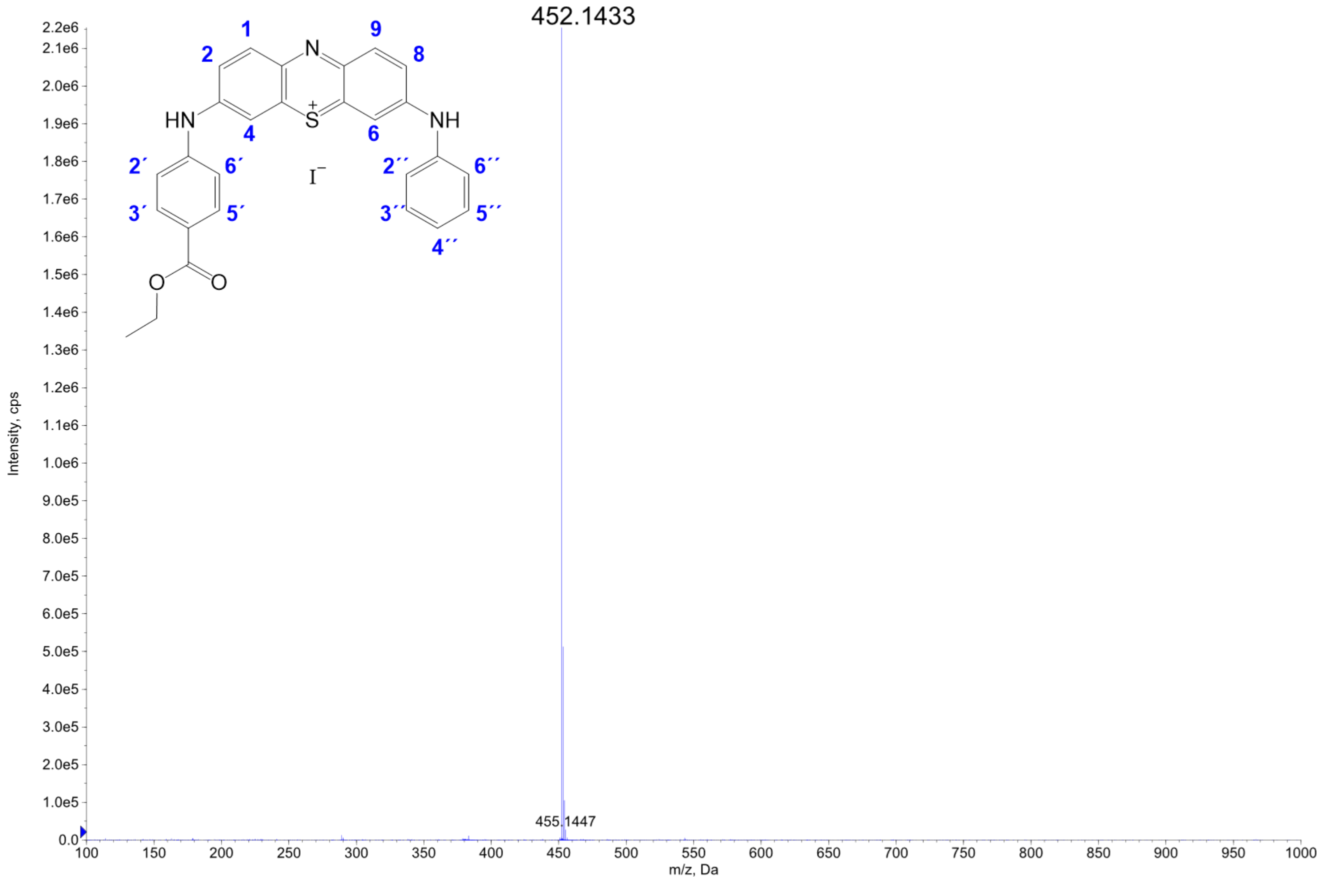


Figure S61. HRMS spectrum of the compound **18**.

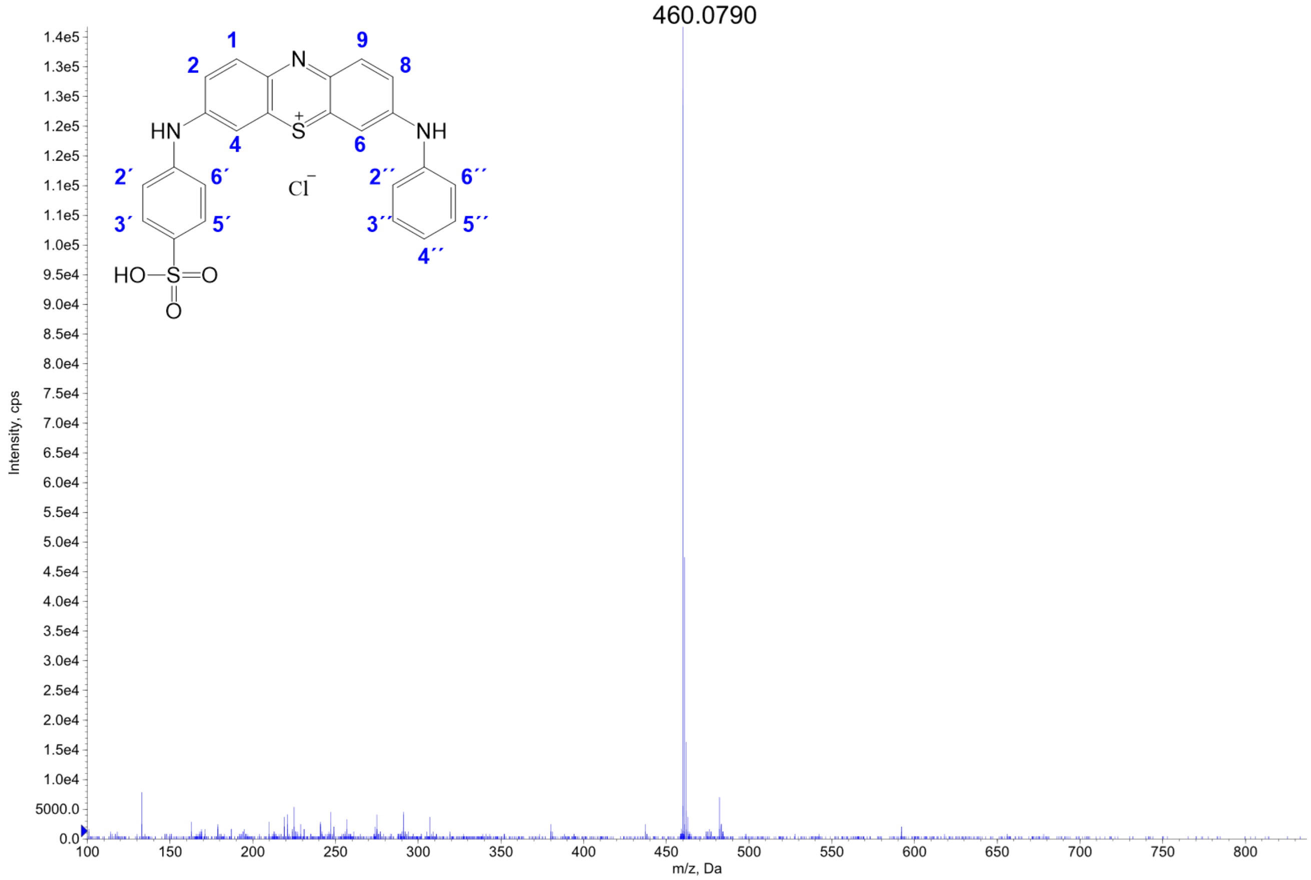


Figure S62. HRMS spectrum of the compound **19**.

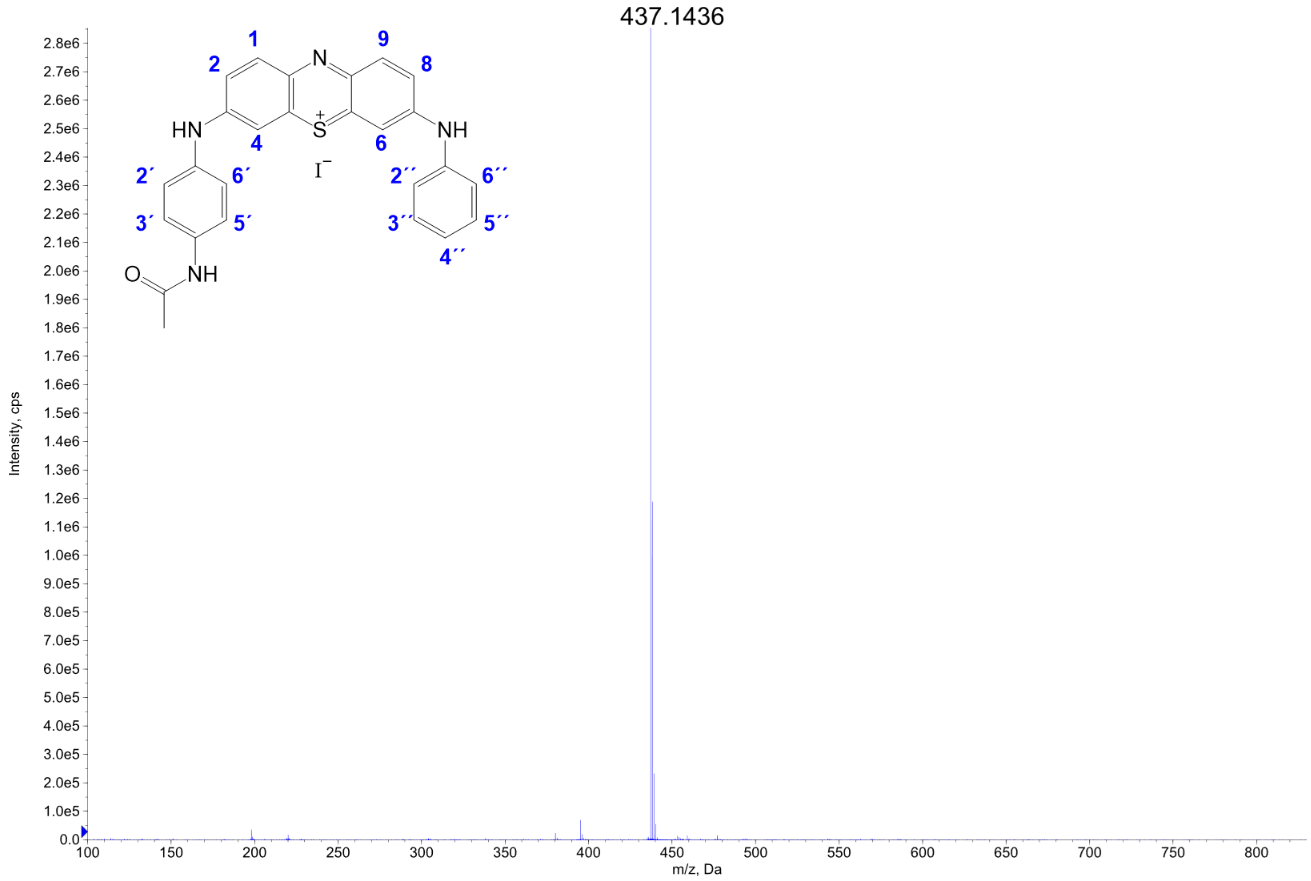


Figure S63. HRMS spectrum of the compound **20**.

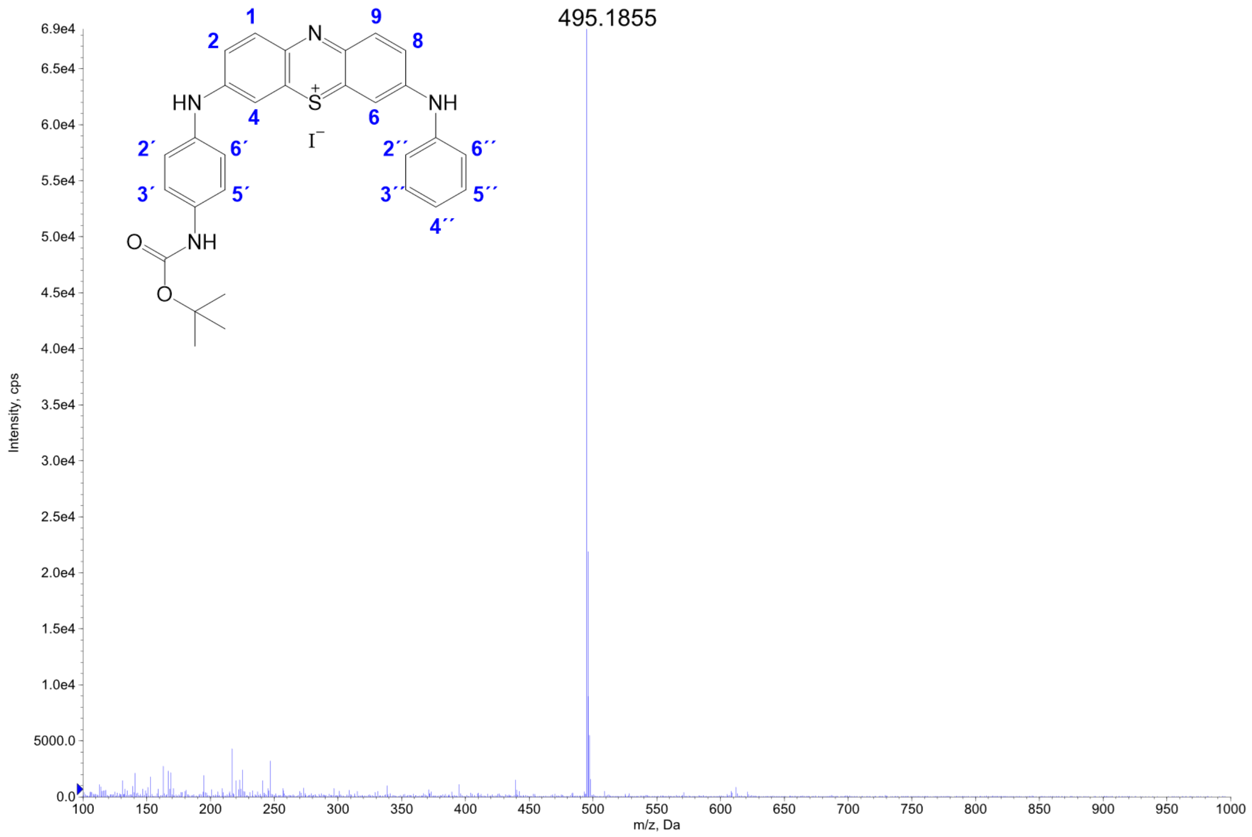


Figure S64. HRMS spectrum of the compound **21**.

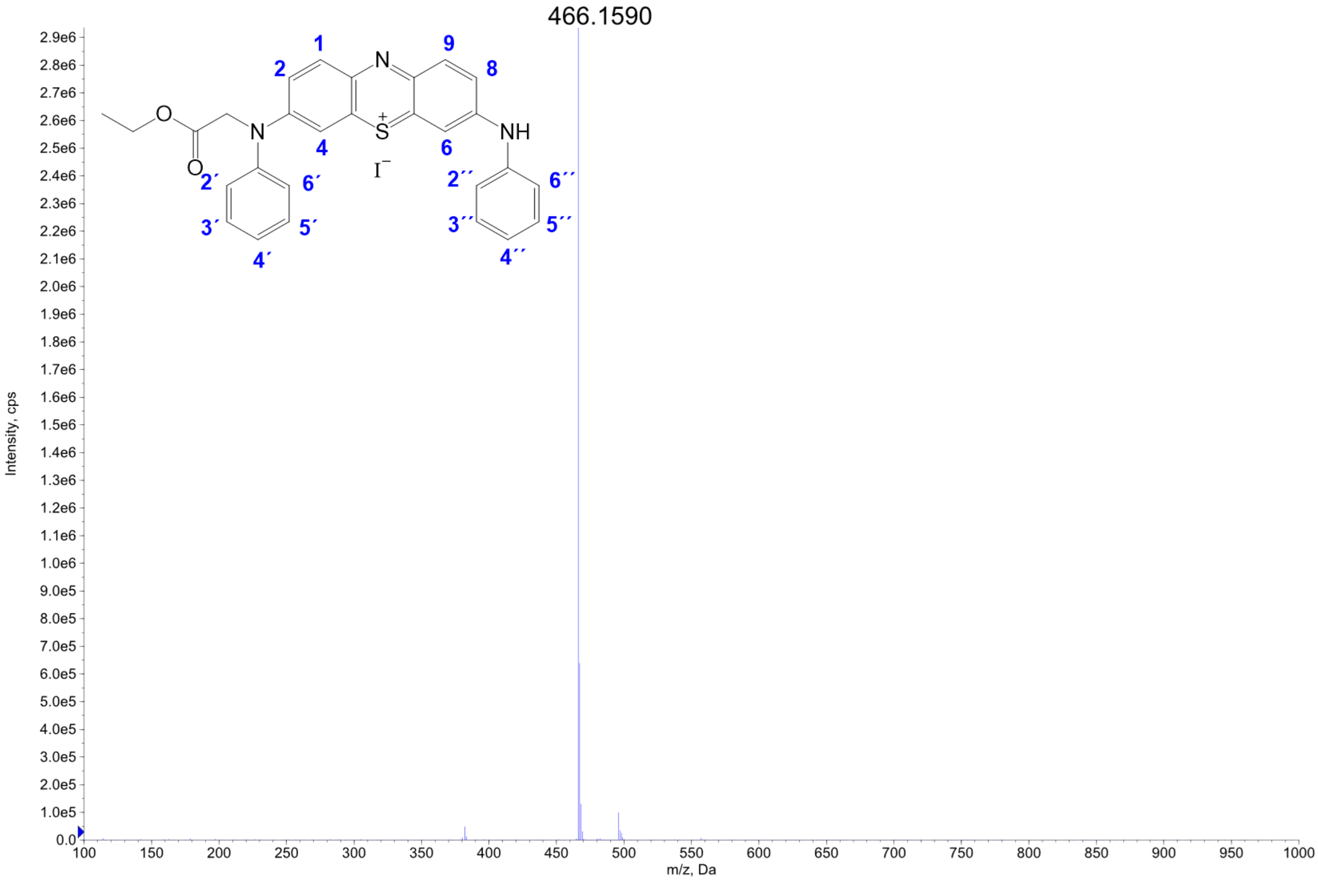


Figure S65. HRMS spectrum of the compound **22**.

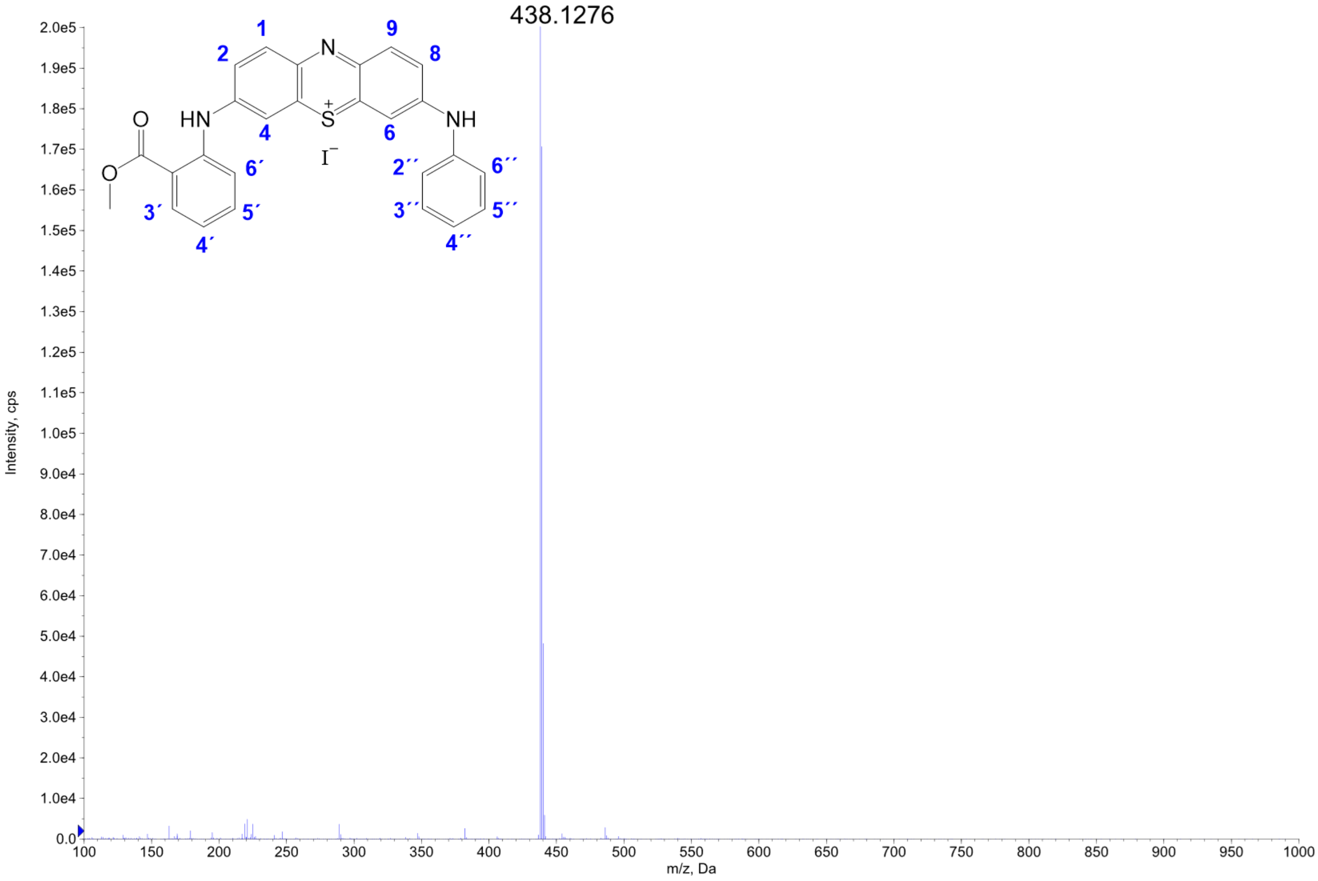


Figure S66. HRMS spectrum of the compound **23**.

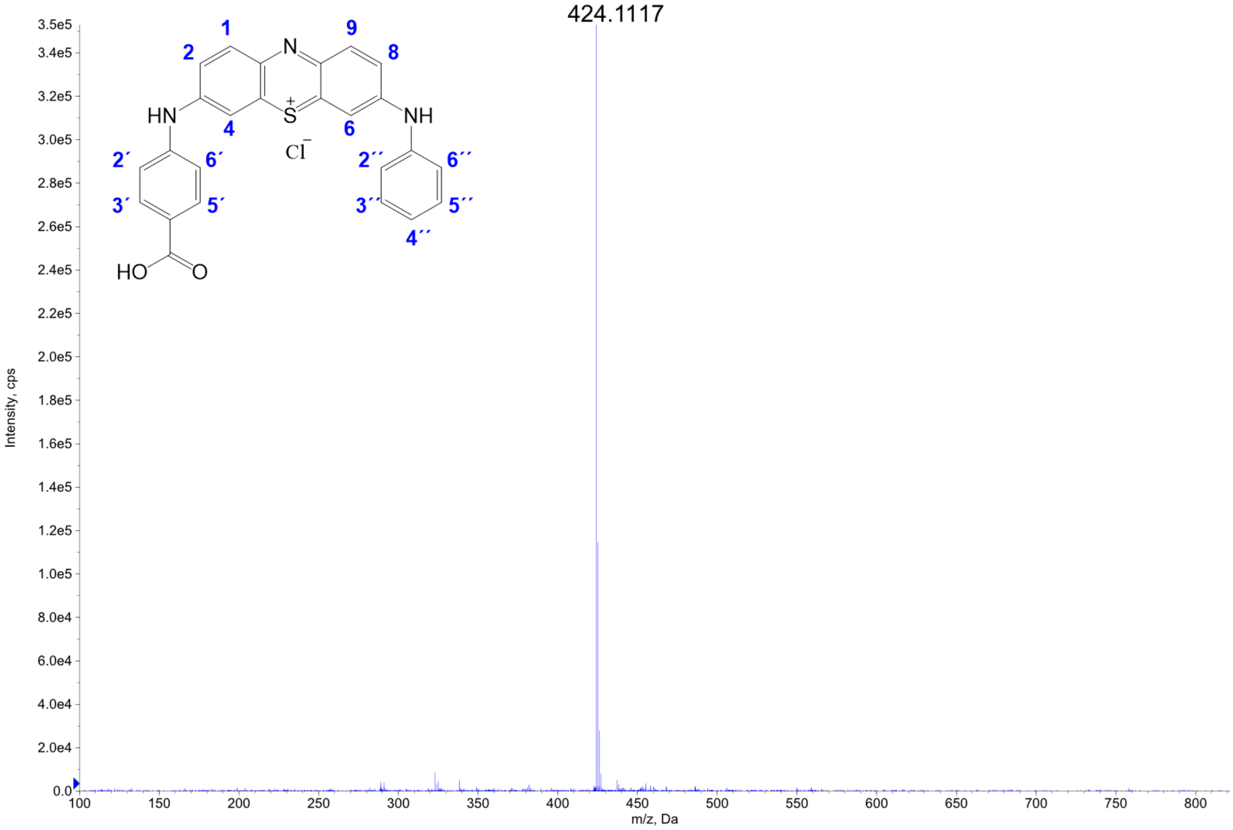


Figure S67. HRMS spectrum of the compound **24**.

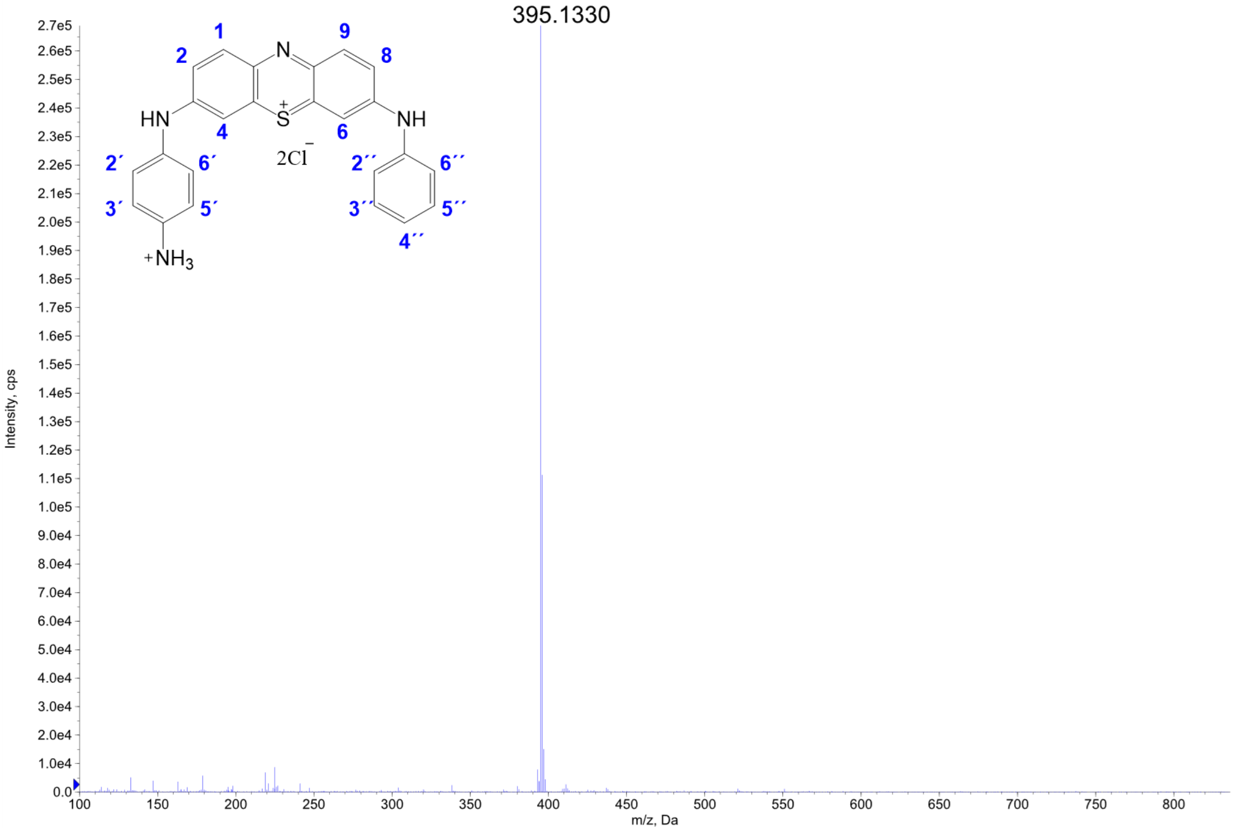


Figure S68. HRMS spectrum of the compound **25**.

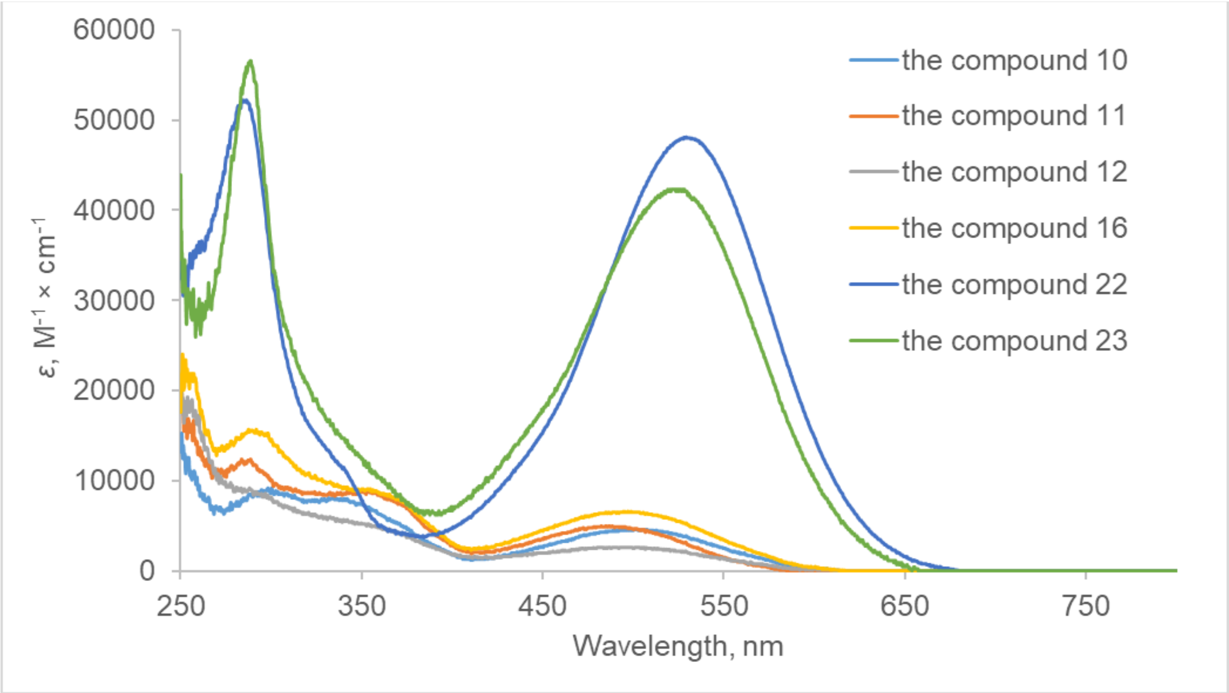


Figure S69. UV-Vis spectra of the compounds **10**, **11**, **12**, **16**, **22**, and **23** (THF, $1 \times 10^{-5}$ M).

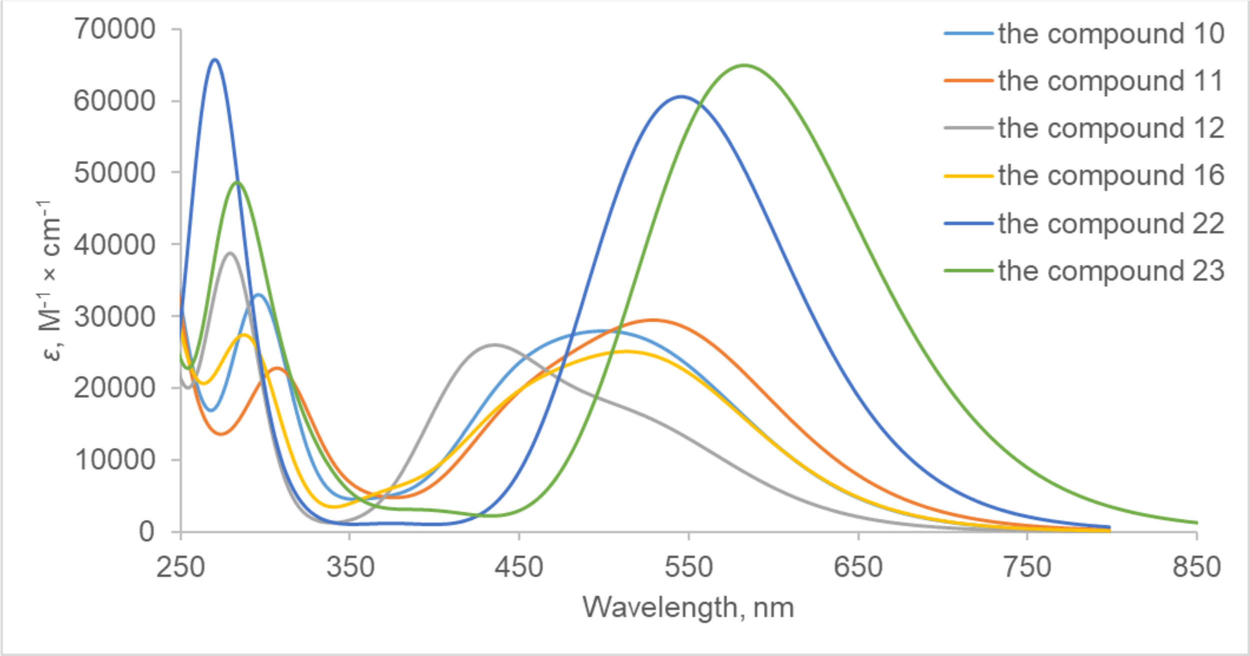


Figure S70. Calculated (TD-DFT M06-HF/6-311++G(d,p)/IEFPCM) UV-Vis absorption spectra of the compounds **10**, **11**, **12**, **16**, **22**, and **23** in THF.

Table S1. Absolute energies, minimum frequencies and calculated atomic coordinates for cations **1, 10-16** (DFT B3LYP/6-311++G(d,p)).

**1**: E = -914.9304313 a.u., ω1=78.6 $cm^{-1}$

| Center Number | Atomic Number | Atomic Type | Coordinates (Angstroms) X | Y | Z |
|---|---|---|---|---|---|
| 1 | 6 | 0 | -3.740514 | -0.362518 | 0.000318 |
| 2 | 6 | 0 | -3.596718 | 1.046692 | 0.000097 |
| 3 | 6 | 0 | -2.349490 | 1.609784 | -0.000143 |
| 4 | 6 | 0 | -1.179339 | 0.788075 | -0.000150 |
| 5 | 6 | 0 | -1.354194 | -0.643894 | 0.000027 |
| 6 | 6 | 0 | -2.642132 | -1.200122 | 0.000288 |
| 7 | 16 | 0 | 0.000050 | -1.707209 | -0.000074 |
| 8 | 6 | 0 | 1.353892 | -0.644081 | -0.000410 |
| 9 | 6 | 0 | 1.179398 | 0.788017 | -0.000666 |
| 10 | 7 | 0 | 0.000089 | 1.413530 | -0.000351 |
| 11 | 6 | 0 | 2.642269 | -1.200256 | -0.000117 |
| 12 | 6 | 0 | 3.740371 | -0.362479 | -0.000073 |
| 13 | 6 | 0 | 3.596781 | 1.046778 | 0.000532 |
| 14 | 6 | 0 | 2.349538 | 1.609853 | 0.000337 |
| 15 | 1 | 0 | -4.733817 | -0.796000 | 0.000525 |
| 16 | 1 | 0 | -4.480069 | 1.672955 | 0.000141 |
| 17 | 1 | 0 | -2.197905 | 2.681793 | -0.000278 |
| 18 | 1 | 0 | -2.772821 | -2.275977 | 0.000480 |
| 19 | 1 | 0 | 2.772738 | -2.276095 | 0.000147 |
| 20 | 1 | 0 | 4.480114 | 1.673012 | 0.000848 |
| 21 | 1 | 0 | 2.197401 | 2.681766 | 0.000551 |
| 22 | 1 | 0 | 4.733766 | -0.795919 | 0.000984 |

**10A**: E = -1468.710065 a.u., ω1=21.5 $cm^{-1}$

| Center Number | Atomic Number | Atomic Type | Coordinates (Angstroms) X | Y | Z |
|---|---|---|---|---|---|
| 1 | 6 | 0 | -7.891726 | 0.760002 | -0.223656 |
| 2 | 6 | 0 | -7.478576 | 1.927103 | 0.446906 |
| 3 | 6 | 0 | -6.147471 | 2.117606 | 0.735951 |
| 4 | 6 | 0 | -5.178646 | 1.147428 | 0.367492 |
| 5 | 6 | 0 | -5.620455 | -0.027781 | -0.308948 |
| 6 | 6 | 0 | -6.976795 | -0.209882 | -0.600624 |
| 7 | 16 | 0 | -4.503813 | -1.273277 | -0.796726 |
| 8 | 6 | 0 | -2.968104 | -0.669988 | -0.258728 |

| | | | | | |
|---|---|---|---|---|---|
| 9 | 6 | 0 | -2.882318 | 0.618349 | 0.410923 |
| 10 | 7 | 0 | -3.888607 | 1.418566 | 0.683778 |
| 11 | 6 | 0 | -1.840369 | -1.432916 | -0.492129 |
| 12 | 6 | 0 | -0.572189 | -0.967164 | -0.086101 |
| 13 | 6 | 0 | -0.465198 | 0.316721 | 0.559187 |
| 14 | 6 | 0 | -1.566804 | 1.064832 | 0.798608 |
| 15 | 7 | 0 | 0.565353 | -1.662566 | -0.290269 |
| 16 | 6 | 0 | 0.723549 | -2.983375 | -0.805611 |
| 17 | 6 | 0 | 1.687173 | -3.199718 | -1.794830 |
| 18 | 6 | 0 | 1.894485 | -4.483208 | -2.281030 |
| 19 | 6 | 0 | 1.145912 | -5.555869 | -1.787362 |
| 20 | 6 | 0 | 0.193407 | -5.333161 | -0.788335 |
| 21 | 6 | 0 | -0.015874 | -4.052099 | -0.290388 |
| 22 | 6 | 0 | 1.417685 | -6.918598 | -2.353397 |
| 23 | 8 | 0 | 0.641467 | -7.861171 | -1.801995 |
| 24 | 8 | 0 | 2.245090 | -7.125619 | -3.208759 |
| 25 | 6 | 0 | 0.835271 | -9.226378 | -2.279930 |
| 26 | 6 | 0 | -0.139866 | -10.114909 | -1.539270 |
| 27 | 1 | 0 | -8.941027 | 0.613511 | -0.450880 |
| 28 | 1 | 0 | -8.211038 | 2.672028 | 0.731504 |
| 29 | 1 | 0 | -5.791773 | 3.002973 | 1.247879 |
| 30 | 1 | 0 | -7.309263 | -1.103296 | -1.116537 |
| 31 | 1 | 0 | -1.920709 | -2.379228 | -1.010042 |
| 32 | 1 | 0 | 0.514515 | 0.670094 | 0.861865 |
| 33 | 1 | 0 | -1.506008 | 2.027580 | 1.290315 |
| 34 | 1 | 0 | 1.427431 | -1.171484 | -0.088726 |
| 35 | 1 | 0 | 2.259956 | -2.367075 | -2.187758 |
| 36 | 1 | 0 | 2.632457 | -4.672610 | -3.050495 |
| 37 | 1 | 0 | -0.366608 | -6.168193 | -0.389087 |
| 38 | 1 | 0 | -0.720497 | -3.891681 | 0.516511 |
| 39 | 1 | 0 | 0.669225 | -9.234745 | -3.358698 |
| 40 | 1 | 0 | 1.874230 | -9.505355 | -2.095091 |
| 41 | 1 | 0 | -0.017168 | -11.146880 | -1.877156 |
| 42 | 1 | 0 | 0.039197 | -10.086695 | -0.462281 |
| 43 | 1 | 0 | -1.172262 | -9.814832 | -1.731707 |

**10B**: E = -1468.7093167 a.u., ω1=20.1 $cm^{-1}$

| Center Number | Atomic Number | Atomic Type | Coordinates (Angstroms) X | Y | Z |
|---|---|---|---|---|---|
| 1 | 6 | 0 | -7.612672 | -2.507051 | -0.332884 |
| 2 | 6 | 0 | -7.766388 | -1.249816 | 0.281732 |
| 3 | 6 | 0 | -6.658844 | -0.485001 | 0.565067 |
| 4 | 6 | 0 | -5.355428 | -0.947990 | 0.245042 |

| | | | | | |
|---|---|---|---|---|---|
| 5 | 6 | 0 | -5.223956 | -2.224735 | -0.376823 |
| 6 | 6 | 0 | -6.357459 | -2.993943 | -0.661215 |
| 7 | 16 | 0 | -3.664999 | -2.876102 | -0.805023 |
| 8 | 6 | 0 | -2.560625 | -1.638031 | -0.292311 |
| 9 | 6 | 0 | -3.060126 | -0.418927 | 0.324824 |
| 10 | 7 | 0 | -4.320759 | -0.129762 | 0.558374 |
| 11 | 6 | 0 | -1.208444 | -1.826644 | -0.483815 |
| 12 | 6 | 0 | -0.276533 | -0.838211 | -0.090539 |
| 13 | 6 | 0 | -0.750842 | 0.366144 | 0.537876 |
| 14 | 6 | 0 | -2.078575 | 0.554886 | 0.732780 |
| 15 | 7 | 0 | 1.039035 | -1.062689 | -0.285225 |
| 16 | 6 | 0 | 2.132373 | -0.164296 | -0.094597 |
| 17 | 6 | 0 | 3.252747 | -0.619064 | 0.606416 |
| 18 | 6 | 0 | 4.346240 | 0.220361 | 0.768447 |
| 19 | 6 | 0 | 4.330327 | 1.513386 | 0.236457 |
| 20 | 6 | 0 | 3.210874 | 1.955839 | -0.474517 |
| 21 | 6 | 0 | 2.114752 | 1.118312 | -0.649620 |
| 22 | 6 | 0 | 5.542030 | 2.372377 | 0.449911 |
| 23 | 8 | 0 | 5.408891 | 3.584506 | -0.104377 |
| 24 | 8 | 0 | 6.517921 | 1.998568 | 1.055920 |
| 25 | 6 | 0 | 6.538236 | 4.496625 | 0.045698 |
| 26 | 6 | 0 | 6.178173 | 5.792054 | -0.647809 |
| 27 | 1 | 0 | -8.486889 | -3.107621 | -0.554692 |
| 28 | 1 | 0 | -8.756856 | -0.889122 | 0.529242 |
| 29 | 1 | 0 | -6.737106 | 0.486602 | 1.036595 |
| 30 | 1 | 0 | -6.254660 | -3.963788 | -1.134232 |
| 31 | 1 | 0 | -0.846272 | -2.736446 | -0.951247 |
| 32 | 1 | 0 | -0.036249 | 1.102730 | 0.877165 |
| 33 | 1 | 0 | -2.456753 | 1.445022 | 1.220030 |
| 34 | 1 | 0 | 1.294443 | -1.993216 | -0.592352 |
| 35 | 1 | 0 | 3.260368 | -1.616015 | 1.032663 |
| 36 | 1 | 0 | 5.222340 | -0.108470 | 1.313286 |
| 37 | 1 | 0 | 3.209449 | 2.948238 | -0.905164 |
| 38 | 1 | 0 | 1.267960 | 1.447576 | -1.239685 |
| 39 | 1 | 0 | 6.723353 | 4.633530 | 1.112697 |
| 40 | 1 | 0 | 7.417801 | 4.021216 | -0.392231 |
| 41 | 1 | 0 | 7.008588 | 6.496028 | -0.552429 |
| 42 | 1 | 0 | 5.989410 | 5.631744 | -1.711501 |
| 43 | 1 | 0 | 5.293121 | 6.248197 | -0.198934 |

**11A**: E = - 1429.38786070 a.u., ω1=20.6 $cm^{-1}$

```
Center     Atomic      Atomic             Coordinates (Angstroms)
 Number     Number       Type             X           Y           Z
 ---------------------------------------------------------------------
```

| | | | | | |
|---|---|---|---|---|---|
| 1 | 6 | 0 | -6.980957 | 0.664434 | -0.126641 |
| 2 | 6 | 0 | -6.953582 | -0.545219 | 0.592467 |
| 3 | 6 | 0 | -5.751410 | -1.160268 | 0.856098 |
| 4 | 6 | 0 | -4.530455 | -0.587835 | 0.414062 |
| 5 | 6 | 0 | -4.582297 | 0.638435 | -0.310148 |
| 6 | 6 | 0 | -5.810445 | 1.253672 | -0.576821 |
| 7 | 16 | 0 | -3.133596 | 1.416161 | -0.889702 |
| 8 | 6 | 0 | -1.865842 | 0.357375 | -0.354251 |
| 9 | 6 | 0 | -2.189048 | -0.860962 | 0.372490 |
| 10 | 7 | 0 | -3.392605 | -1.266111 | 0.708850 |
| 11 | 6 | 0 | -0.558388 | 0.693118 | -0.647545 |
| 12 | 6 | 0 | 0.501246 | -0.151326 | -0.249306 |
| 13 | 6 | 0 | 0.193931 | -1.381831 | 0.438727 |
| 14 | 6 | 0 | -1.082869 | -1.710692 | 0.739741 |
| 15 | 7 | 0 | 1.806484 | 0.087290 | -0.489559 |
| 16 | 6 | 0 | 2.452829 | 1.210881 | -1.041887 |
| 17 | 6 | 0 | 3.637975 | 1.012981 | -1.796883 |
| 18 | 6 | 0 | 4.288948 | 2.127806 | -2.336271 |
| 19 | 6 | 0 | 3.808016 | 3.414465 | -2.125326 |
| 20 | 6 | 0 | 2.671531 | 3.603263 | -1.342078 |
| 21 | 6 | 0 | 2.000582 | 2.512854 | -0.800428 |
| 22 | 1 | 0 | -7.929254 | 1.145936 | -0.333895 |
| 23 | 1 | 0 | -7.880064 | -0.989147 | 0.934564 |
| 24 | 1 | 0 | -5.691466 | -2.092732 | 1.403269 |
| 25 | 1 | 0 | -5.845505 | 2.185262 | -1.129854 |
| 26 | 1 | 0 | -0.350665 | 1.584859 | -1.221629 |
| 27 | 1 | 0 | 1.013005 | -2.032179 | 0.724045 |
| 28 | 1 | 0 | -1.326575 | -2.623500 | 1.268610 |
| 29 | 1 | 0 | 2.437292 | -0.721166 | -0.432094 |
| 30 | 6 | 0 | 4.219578 | -0.349488 | -1.970670 |
| 31 | 1 | 0 | 5.187036 | 1.970384 | -2.917607 |
| 32 | 1 | 0 | 2.312933 | 4.604430 | -1.134211 |
| 33 | 1 | 0 | 1.155562 | 2.678518 | -0.146332 |
| 34 | 8 | 0 | 3.841054 | -1.338108 | -1.357239 |
| 35 | 8 | 0 | 5.215148 | -0.383967 | -2.848894 |
| 36 | 6 | 0 | 5.871023 | -1.661925 | -3.046914 |
| 37 | 1 | 0 | 4.329120 | 4.263863 | -2.548858 |
| 38 | 1 | 0 | 5.152987 | -2.396598 | -3.409712 |
| 39 | 1 | 0 | 6.309226 | -2.004196 | -2.109972 |
| 40 | 1 | 0 | 6.640636 | -1.471159 | -3.789461 |

**11B**: E = -1429.3872594 a.u., ω1=22.6 $cm^{-1}$

| Center Number | Atomic Number | Atomic Type | Coordinates (Angstroms) X | Y | Z |
|---|---|---|---|---|---|

----------------------------------------------------------------------

| | | | | | |
|---|---|---|---|---|---|
| 1 | 6 | 0 | -7.583332 | -2.730344 | 0.221758 |
| 2 | 6 | 0 | -7.815382 | -1.424646 | -0.249897 |
| 3 | 6 | 0 | -6.755827 | -0.571223 | -0.453990 |
| 4 | 6 | 0 | -5.424723 | -0.990324 | -0.195263 |
| 5 | 6 | 0 | -5.213163 | -2.317453 | 0.280654 |
| 6 | 6 | 0 | -6.298287 | -3.176672 | 0.485879 |
| 7 | 16 | 0 | -3.614326 | -2.924218 | 0.620573 |
| 8 | 6 | 0 | -2.588699 | -1.571319 | 0.247595 |
| 9 | 6 | 0 | -3.165047 | -0.322650 | -0.227308 |
| 10 | 7 | 0 | -4.441948 | -0.082457 | -0.422716 |
| 11 | 6 | 0 | -1.227958 | -1.698873 | 0.416360 |
| 12 | 6 | 0 | -0.354718 | -0.617282 | 0.145842 |
| 13 | 6 | 0 | -0.905556 | 0.613111 | -0.358731 |
| 14 | 6 | 0 | -2.243599 | 0.742544 | -0.533996 |
| 15 | 7 | 0 | 0.966058 | -0.817616 | 0.333623 |
| 16 | 6 | 0 | 2.046713 | 0.085182 | 0.284735 |
| 17 | 6 | 0 | 3.311780 | -0.387354 | -0.150377 |
| 18 | 6 | 0 | 4.388512 | 0.505252 | -0.189744 |
| 19 | 6 | 0 | 4.241249 | 1.828886 | 0.207218 |
| 20 | 6 | 0 | 3.007389 | 2.271869 | 0.678954 |
| 21 | 6 | 0 | 1.919941 | 1.407167 | 0.725481 |
| 22 | 1 | 0 | -8.419151 | -3.401060 | 0.381964 |
| 23 | 1 | 0 | -8.827510 | -1.096153 | -0.450123 |
| 24 | 1 | 0 | -6.894761 | 0.440273 | -0.814632 |
| 25 | 1 | 0 | -6.134439 | -4.185082 | 0.848119 |
| 26 | 1 | 0 | -0.806562 | -2.629791 | 0.780035 |
| 27 | 1 | 0 | -0.243223 | 1.418301 | -0.640909 |
| 28 | 1 | 0 | -2.674971 | 1.651500 | -0.934654 |
| 29 | 1 | 0 | 1.287455 | -1.792155 | 0.364937 |
| 30 | 6 | 0 | 3.514071 | -1.820830 | -0.507462 |
| 31 | 1 | 0 | 5.348573 | 0.141922 | -0.529629 |
| 32 | 1 | 0 | 2.891915 | 3.289735 | 1.032074 |
| 33 | 1 | 0 | 0.984662 | 1.747603 | 1.148946 |
| 34 | 8 | 0 | 2.694820 | -2.702980 | -0.287819 |
| 35 | 8 | 0 | 4.692540 | -2.056701 | -1.073067 |
| 36 | 6 | 0 | 4.993785 | -3.433111 | -1.413379 |
| 37 | 1 | 0 | 5.088079 | 2.502677 | 0.171442 |
| 38 | 1 | 0 | 4.259480 | -3.810303 | -2.124385 |
| 39 | 1 | 0 | 4.988561 | -4.048606 | -0.514326 |
| 40 | 1 | 0 | 5.984600 | -3.402815 | -1.857781 |

**12A**: E = -1508.0227938 a.u., ω1=15.8 $cm^{-1}$

Center Atomic Atomic Coordinates (Angstroms)

| Number | Number | Type | X | Y | Z |
|---|---|---|---|---|---|
| 1 | 6 | 0 | -6.484988 | 0.504154 | -0.117987 |
| 2 | 6 | 0 | -6.319132 | -0.040377 | 1.169348 |
| 3 | 6 | 0 | -5.068799 | -0.425285 | 1.594859 |
| 4 | 6 | 0 | -3.937430 | -0.279883 | 0.750454 |
| 5 | 6 | 0 | -4.128288 | 0.274817 | -0.548402 |
| 6 | 6 | 0 | -5.404971 | 0.662148 | -0.971368 |
| 7 | 16 | 0 | -2.798419 | 0.494793 | -1.653657 |
| 8 | 6 | 0 | -1.432017 | -0.099524 | -0.758629 |
| 9 | 6 | 0 | -1.611100 | -0.614777 | 0.589974 |
| 10 | 7 | 0 | -2.743199 | -0.687168 | 1.250904 |
| 11 | 6 | 0 | -0.189919 | -0.071099 | -1.351944 |
| 12 | 6 | 0 | 0.957497 | -0.546105 | -0.666474 |
| 13 | 6 | 0 | 0.793028 | -1.055622 | 0.675119 |
| 14 | 6 | 0 | -0.426877 | -1.087257 | 1.260981 |
| 15 | 7 | 0 | 2.167356 | -0.537163 | -1.263675 |
| 16 | 6 | 0 | 2.359699 | 0.081575 | -2.562229 |
| 17 | 6 | 0 | 2.376535 | -0.718060 | -3.704297 |
| 18 | 6 | 0 | 2.599829 | -0.126519 | -4.946398 |
| 19 | 6 | 0 | 2.805819 | 1.249448 | -5.038933 |
| 20 | 6 | 0 | 2.789518 | 2.038719 | -3.889756 |
| 21 | 6 | 0 | 2.566674 | 1.458575 | -2.641799 |
| 22 | 1 | 0 | -7.470726 | 0.806438 | -0.451265 |
| 23 | 1 | 0 | -7.176724 | -0.154556 | 1.820447 |
| 24 | 1 | 0 | -4.901925 | -0.848640 | 2.577416 |
| 25 | 1 | 0 | -5.547169 | 1.083595 | -1.959841 |
| 26 | 1 | 0 | -0.078308 | 0.315953 | -2.355486 |
| 27 | 1 | 0 | 1.650622 | -1.402914 | 1.231644 |
| 28 | 1 | 0 | -0.559382 | -1.462067 | 2.268234 |
| 29 | 1 | 0 | 2.613870 | -0.741109 | -5.838575 |
| 30 | 1 | 0 | 2.955279 | 3.107153 | -3.960901 |
| 31 | 1 | 0 | 2.562607 | 2.054921 | -1.737310 |
| 32 | 1 | 0 | 2.982711 | 1.705381 | -6.005840 |
| 33 | 1 | 0 | 2.215553 | -1.786981 | -3.621330 |
| 34 | 6 | 0 | 3.383080 | -0.997641 | -0.594580 |
| 35 | 1 | 0 | 3.216696 | -1.941411 | -0.072002 |
| 36 | 1 | 0 | 4.135277 | -1.193736 | -1.358030 |
| 37 | 6 | 0 | 3.944737 | 0.039808 | 0.389926 |
| 38 | 8 | 0 | 3.402017 | 1.082211 | 0.656887 |
| 39 | 8 | 0 | 5.095279 | -0.398592 | 0.888729 |
| 40 | 6 | 0 | 5.786735 | 0.470787 | 1.847804 |
| 41 | 1 | 0 | 5.115794 | 0.635868 | 2.692512 |
| 42 | 1 | 0 | 5.966283 | 1.429226 | 1.358361 |
| 43 | 6 | 0 | 7.067742 | -0.223017 | 2.251458 |
| 44 | 1 | 0 | 7.604747 | 0.403310 | 2.968036 |

| | | | | | |
|---|---|---|---|---|---|
| 45 | 1 | 0 | 6.864553 | -1.185296 | 2.725572 |
| 46 | 1 | 0 | 7.716615 | -0.386766 | 1.388934 |

**12B**: E = -1508.0226831 a.u., ω1=16.2 cm$^{-1}$

| Center Number | Atomic Number | Atomic Type | Coordinates (Angstroms) X | Y | Z |
|---|---|---|---|---|---|
| 1 | 6 | 0 | -6.235759 | 1.285540 | -0.673412 |
| 2 | 6 | 0 | -6.434473 | -0.084350 | -0.418645 |
| 3 | 6 | 0 | -5.356826 | -0.894192 | -0.143211 |
| 4 | 6 | 0 | -4.040488 | -0.365196 | -0.110351 |
| 5 | 6 | 0 | -3.863346 | 1.024659 | -0.368676 |
| 6 | 6 | 0 | -4.965899 | 1.839269 | -0.650317 |
| 7 | 16 | 0 | -2.284342 | 1.762933 | -0.345202 |
| 8 | 6 | 0 | -1.226877 | 0.439742 | 0.039146 |
| 9 | 6 | 0 | -1.769676 | -0.896297 | 0.231333 |
| 10 | 7 | 0 | -3.035951 | -1.236366 | 0.163094 |
| 11 | 6 | 0 | 0.126541 | 0.682042 | 0.139644 |
| 12 | 6 | 0 | 1.034504 | -0.367930 | 0.428086 |
| 13 | 6 | 0 | 0.506913 | -1.700060 | 0.617221 |
| 14 | 6 | 0 | -0.821356 | -1.940623 | 0.526755 |
| 15 | 7 | 0 | 2.360342 | -0.150764 | 0.552677 |
| 16 | 6 | 0 | 3.302642 | -1.248949 | 0.668702 |
| 17 | 6 | 0 | 3.847018 | -1.554635 | 1.915539 |
| 18 | 6 | 0 | 4.778008 | -2.586444 | 2.017512 |
| 19 | 6 | 0 | 5.158739 | -3.300039 | 0.881521 |
| 20 | 6 | 0 | 4.611580 | -2.982351 | -0.360627 |
| 21 | 6 | 0 | 3.680485 | -1.951236 | -0.475342 |
| 22 | 1 | 0 | -7.085870 | 1.921112 | -0.891295 |
| 23 | 1 | 0 | -7.435366 | -0.496799 | -0.441773 |
| 24 | 1 | 0 | -5.469962 | -1.952632 | 0.055330 |
| 25 | 1 | 0 | -4.827224 | 2.896001 | -0.847810 |
| 26 | 1 | 0 | 0.491393 | 1.683237 | -0.038947 |
| 27 | 1 | 0 | 1.191292 | -2.504762 | 0.844317 |
| 28 | 1 | 0 | -1.226674 | -2.933483 | 0.677058 |
| 29 | 1 | 0 | 5.201820 | -2.832878 | 2.983672 |
| 30 | 1 | 0 | 4.911681 | -3.532511 | -1.244507 |
| 31 | 1 | 0 | 3.258626 | -1.680418 | -1.435634 |
| 32 | 1 | 0 | 5.883453 | -4.101204 | 0.964556 |
| 33 | 1 | 0 | 3.540592 | -0.998116 | 2.794127 |
| 34 | 6 | 0 | 2.950291 | 1.178715 | 0.419538 |
| 35 | 1 | 0 | 2.354151 | 1.927854 | 0.945086 |
| 36 | 1 | 0 | 3.929390 | 1.166435 | 0.897499 |
| 37 | 6 | 0 | 3.129072 | 1.603568 | -1.046514 |

| | | | | | |
|---|---|---|---|---|---|
| 38 | 8 | 0 | 2.731551 | 0.968032 | -1.989872 |
| 39 | 8 | 0 | 3.774268 | 2.764584 | -1.096043 |
| 40 | 6 | 0 | 4.053267 | 3.322349 | -2.424131 |
| 41 | 1 | 0 | 3.099790 | 3.457781 | -2.937516 |
| 42 | 1 | 0 | 4.639937 | 2.587021 | -2.976890 |
| 43 | 6 | 0 | 4.795188 | 4.624521 | -2.225440 |
| 44 | 1 | 0 | 5.016678 | 5.064191 | -3.201020 |
| 45 | 1 | 0 | 4.195728 | 5.339003 | -1.657750 |
| 46 | 1 | 0 | 5.739524 | 4.464805 | -1.701570 |

**13A**: E = -1405.9881632 a.u., ω1=20.0 $cm^{-1}$

| Center Number | Atomic Number | Atomic Type | Coordinates (Angstroms) X | Y | Z |
|---|---|---|---|---|---|
| 1 | 6 | 0 | -7.167848 | 0.171851 | 0.174868 |
| 2 | 6 | 0 | -6.871248 | 1.487810 | -0.231679 |
| 3 | 6 | 0 | -5.564219 | 1.869963 | -0.419282 |
| 4 | 6 | 0 | -4.502008 | 0.949847 | -0.209949 |
| 5 | 6 | 0 | -4.827118 | -0.379012 | 0.199648 |
| 6 | 6 | 0 | -6.161304 | -0.755303 | 0.390566 |
| 7 | 16 | 0 | -3.591816 | -1.573724 | 0.475446 |
| 8 | 6 | 0 | -2.122156 | -0.719917 | 0.134252 |
| 9 | 6 | 0 | -2.161773 | 0.677503 | -0.260380 |
| 10 | 7 | 0 | -3.245305 | 1.409421 | -0.410504 |
| 11 | 6 | 0 | -0.921281 | -1.396297 | 0.257514 |
| 12 | 6 | 0 | 0.294230 | -0.732190 | 0.003146 |
| 13 | 6 | 0 | 0.275348 | 0.658111 | -0.369800 |
| 14 | 6 | 0 | -0.896221 | 1.324304 | -0.498487 |
| 15 | 7 | 0 | 1.500771 | -1.334952 | 0.113069 |
| 16 | 6 | 0 | 1.787727 | -2.707047 | 0.364886 |
| 17 | 6 | 0 | 2.746509 | -3.019822 | 1.333515 |
| 18 | 6 | 0 | 3.080069 | -4.346736 | 1.574159 |
| 19 | 6 | 0 | 2.440976 | -5.339978 | 0.841971 |
| 20 | 6 | 0 | 1.496991 | -5.047563 | -0.135228 |
| 21 | 6 | 0 | 1.174617 | -3.719007 | -0.381761 |
| 22 | 1 | 0 | -8.199509 | -0.124788 | 0.322648 |
| 23 | 1 | 0 | -7.675466 | 2.194501 | -0.393538 |
| 24 | 1 | 0 | -5.297000 | 2.872460 | -0.729179 |
| 25 | 1 | 0 | -6.405508 | -1.763947 | 0.703344 |
| 26 | 1 | 0 | -0.912491 | -2.430525 | 0.574465 |
| 27 | 1 | 0 | 1.216331 | 1.163427 | -0.557812 |
| 28 | 1 | 0 | -0.929253 | 2.367543 | -0.786492 |
| 29 | 1 | 0 | 2.307937 | -0.727030 | 0.054604 |
| 30 | 1 | 0 | 1.044844 | -5.851832 | -0.699512 |

| | | | | | |
|---|---|---|---|---|---|
| 31 | 1 | 0 | 0.477263 | -3.471625 | -1.172170 |
| 32 | 7 | 0 | 2.783943 | -6.761700 | 1.104790 |
| 33 | 1 | 0 | 3.220408 | -2.230632 | 1.905694 |
| 34 | 8 | 0 | 2.165573 | -7.608488 | 0.478091 |
| 35 | 8 | 0 | 3.655926 | -6.981371 | 1.930346 |
| 36 | 1 | 0 | 3.814908 | -4.618114 | 2.319855 |

**13B**: E = -1405.987215 a.u., ω1=19.8 $cm^{-1}$

| Center Number | Atomic Number | Atomic Type | Coordinates (Angstroms) X | Y | Z |
|---|---|---|---|---|---|
| 1 | 6 | 0 | -7.724859 | -2.683241 | 0.200706 |
| 2 | 6 | 0 | -7.953766 | -1.338042 | -0.149349 |
| 3 | 6 | 0 | -6.890739 | -0.482728 | -0.317225 |
| 4 | 6 | 0 | -5.556780 | -0.939712 | -0.142251 |
| 5 | 6 | 0 | -5.349401 | -2.307743 | 0.211395 |
| 6 | 6 | 0 | -6.439481 | -3.168310 | 0.380443 |
| 7 | 16 | 0 | -3.750740 | -2.957311 | 0.442978 |
| 8 | 6 | 0 | -2.718619 | -1.594809 | 0.146963 |
| 9 | 6 | 0 | -3.288588 | -0.303504 | -0.198059 |
| 10 | 7 | 0 | -4.570174 | -0.031849 | -0.324902 |
| 11 | 6 | 0 | -1.351340 | -1.755914 | 0.252099 |
| 12 | 6 | 0 | -0.477621 | -0.669177 | 0.031978 |
| 13 | 6 | 0 | -1.022536 | 0.610746 | -0.330447 |
| 14 | 6 | 0 | -2.364459 | 0.774489 | -0.438676 |
| 15 | 7 | 0 | 0.856782 | -0.871219 | 0.134968 |
| 16 | 6 | 0 | 1.907869 | 0.089382 | 0.104912 |
| 17 | 6 | 0 | 3.040163 | -0.190604 | -0.666946 |
| 18 | 6 | 0 | 4.103826 | 0.702197 | -0.684360 |
| 19 | 6 | 0 | 4.011899 | 1.866775 | 0.068570 |
| 20 | 6 | 0 | 2.900772 | 2.155774 | 0.851498 |
| 21 | 6 | 0 | 1.844339 | 1.253822 | 0.877760 |
| 22 | 1 | 0 | -8.565463 | -3.354255 | 0.332263 |
| 23 | 1 | 0 | -8.967538 | -0.982587 | -0.284553 |
| 24 | 1 | 0 | -7.026360 | 0.557593 | -0.585159 |
| 25 | 1 | 0 | -6.280164 | -4.206190 | 0.649205 |
| 26 | 1 | 0 | -0.936862 | -2.722177 | 0.520003 |
| 27 | 1 | 0 | -0.353873 | 1.432406 | -0.544905 |
| 28 | 1 | 0 | -2.795387 | 1.725057 | -0.727610 |
| 29 | 1 | 0 | 1.157074 | -1.833339 | 0.230129 |
| 30 | 1 | 0 | 2.882736 | 3.062272 | 1.441160 |
| 31 | 1 | 0 | 0.993134 | 1.440901 | 1.520169 |
| 32 | 7 | 0 | 5.143451 | 2.829633 | 0.044916 |
| 33 | 1 | 0 | 3.083958 | -1.095552 | -1.261937 |

| | | | | | |
|---|---|---|---|---|---|
| 34 | 8 | 0 | 5.013332 | 3.857944 | 0.690906 |
| 35 | 8 | 0 | 6.120680 | 2.524588 | -0.620310 |
| 36 | 1 | 0 | 4.989733 | 0.513683 | -1.275334 |

**14A**: E = -1409.5088322 a.u., ω1=17.1 $cm^{-1}$

| Center Number | Atomic Number | Atomic Type | Coordinates (Angstroms) X | Y | Z |
|---|---|---|---|---|---|
| 1 | 6 | 0 | -7.649332 | 0.123599 | 0.141191 |
| 2 | 6 | 0 | -7.367306 | 1.442493 | -0.260976 |
| 3 | 6 | 0 | -6.062576 | 1.842008 | -0.437459 |
| 4 | 6 | 0 | -4.990606 | 0.937459 | -0.221640 |
| 5 | 6 | 0 | -5.300068 | -0.393759 | 0.182734 |
| 6 | 6 | 0 | -6.629964 | -0.788742 | 0.363146 |
| 7 | 16 | 0 | -4.049156 | -1.575304 | 0.467172 |
| 8 | 6 | 0 | -2.586474 | -0.698731 | 0.137585 |
| 9 | 6 | 0 | -2.645505 | 0.700705 | -0.255352 |
| 10 | 7 | 0 | -3.735645 | 1.417227 | -0.411862 |
| 11 | 6 | 0 | -1.378816 | -1.355554 | 0.268627 |
| 12 | 6 | 0 | -0.167767 | -0.672647 | 0.025874 |
| 13 | 6 | 0 | -0.205818 | 0.719649 | -0.341643 |
| 14 | 6 | 0 | -1.385767 | 1.367290 | -0.478895 |
| 15 | 7 | 0 | 1.041432 | -1.259802 | 0.137800 |
| 16 | 6 | 0 | 1.349364 | -2.631223 | 0.362888 |
| 17 | 6 | 0 | 2.320106 | -2.960058 | 1.311761 |
| 18 | 6 | 0 | 2.677094 | -4.282743 | 1.532359 |
| 19 | 6 | 0 | 2.083427 | -5.315603 | 0.787383 |
| 20 | 6 | 0 | 1.110787 | -4.969429 | -0.170687 |
| 21 | 6 | 0 | 0.749505 | -3.650427 | -0.385184 |
| 22 | 1 | 0 | -8.677615 | -0.188164 | 0.280854 |
| 23 | 1 | 0 | -8.178842 | 2.139453 | -0.428580 |
| 24 | 1 | 0 | -5.806944 | 2.848674 | -0.743693 |
| 25 | 1 | 0 | -6.861481 | -1.801335 | 0.672972 |
| 26 | 1 | 0 | -1.353843 | -2.390163 | 0.583640 |
| 27 | 1 | 0 | 0.729108 | 1.240248 | -0.518630 |
| 28 | 1 | 0 | -1.432865 | 2.411258 | -0.762200 |
| 29 | 1 | 0 | 1.839609 | -0.640420 | 0.072683 |
| 30 | 1 | 0 | 0.659738 | -5.748598 | -0.774813 |
| 31 | 1 | 0 | 0.035070 | -3.412484 | -1.163842 |
| 32 | 7 | 0 | 2.365437 | -6.669246 | 0.965843 |
| 33 | 1 | 0 | 2.779469 | -2.179791 | 1.908638 |
| 34 | 1 | 0 | 3.393441 | -4.504291 | 2.308604 |
| 35 | 6 | 0 | 3.489876 | -7.377127 | 1.418828 |
| 36 | 8 | 0 | 3.408679 | -8.584642 | 1.478582 |
| 37 | 6 | 0 | 4.752141 | -6.629932 | 1.777006 |

| | | | | | |
|---|---|---|---|---|---|
| 38 | 1 | 0 | 1.657310 | -7.314448 | 0.635842 |
| 39 | 1 | 0 | 4.694776 | -6.260331 | 2.804928 |
| 40 | 1 | 0 | 4.948731 | -5.787514 | 1.112181 |
| 41 | 1 | 0 | 5.574209 | -7.341409 | 1.724442 |

**14B**: E = -1409.5080404 a.u., ω1=16.8 cm$^{-1}$

| Center Number | Atomic Number | Atomic Type | Coordinates (Angstroms) X | Y | Z |
|---|---|---|---|---|---|
| 1 | 6 | 0 | -7.799330 | -2.619278 | 0.209148 |
| 2 | 6 | 0 | -8.002401 | -1.276577 | -0.159932 |
| 3 | 6 | 0 | -6.922081 | -0.442770 | -0.336012 |
| 4 | 6 | 0 | -5.598405 | -0.919737 | -0.150432 |
| 5 | 6 | 0 | -5.416848 | -2.283461 | 0.222344 |
| 6 | 6 | 0 | -6.521938 | -3.122480 | 0.399618 |
| 7 | 16 | 0 | -3.828229 | -2.960472 | 0.470087 |
| 8 | 6 | 0 | -2.770131 | -1.618290 | 0.155498 |
| 9 | 6 | 0 | -3.319265 | -0.321304 | -0.209759 |
| 10 | 7 | 0 | -4.593197 | -0.028648 | -0.343728 |
| 11 | 6 | 0 | -1.408448 | -1.800202 | 0.264488 |
| 12 | 6 | 0 | -0.512444 | -0.730658 | 0.030222 |
| 13 | 6 | 0 | -1.035998 | 0.552783 | -0.354321 |
| 14 | 6 | 0 | -2.373955 | 0.736627 | -0.467378 |
| 15 | 7 | 0 | 0.814081 | -0.948490 | 0.145304 |
| 16 | 6 | 0 | 1.875848 | -0.000041 | 0.127864 |
| 17 | 6 | 0 | 3.017638 | -0.275250 | -0.628042 |
| 18 | 6 | 0 | 4.089093 | 0.606269 | -0.640534 |
| 19 | 6 | 0 | 4.052936 | 1.784623 | 0.124486 |
| 20 | 6 | 0 | 2.897281 | 2.047199 | 0.885838 |
| 21 | 6 | 0 | 1.826902 | 1.169616 | 0.894404 |
| 22 | 1 | 0 | -8.651452 | -3.274163 | 0.347376 |
| 23 | 1 | 0 | -9.008761 | -0.903828 | -0.303855 |
| 24 | 1 | 0 | -7.038770 | 0.595867 | -0.619063 |
| 25 | 1 | 0 | -6.380440 | -4.158998 | 0.683393 |
| 26 | 1 | 0 | -1.010030 | -2.768950 | 0.547929 |
| 27 | 1 | 0 | -0.350726 | 1.357285 | -0.581600 |
| 28 | 1 | 0 | -2.788990 | 1.688371 | -0.775085 |
| 29 | 1 | 0 | 1.096979 | -1.912859 | 0.269702 |
| 30 | 1 | 0 | 2.858069 | 2.938105 | 1.502392 |
| 31 | 1 | 0 | 0.972628 | 1.375365 | 1.528025 |
| 32 | 7 | 0 | 5.073719 | 2.733415 | 0.148200 |
| 33 | 1 | 0 | 3.059198 | -1.169670 | -1.239870 |
| 34 | 1 | 0 | 4.934164 | 0.386149 | -1.274361 |
| 35 | 6 | 0 | 6.459877 | 2.679309 | -0.068121 |

| | | | | | |
|---|---|---|---|---|---|
| 36 | 8 | 0 | 7.082962 | 3.715329 | 0.012864 |
| 37 | 6 | 0 | 7.123642 | 1.354171 | -0.357178 |
| 38 | 1 | 0 | 4.805977 | 3.660399 | 0.457738 |
| 39 | 1 | 0 | 7.034919 | 1.110014 | -1.419715 |
| 40 | 1 | 0 | 6.703220 | 0.534051 | 0.226821 |
| 41 | 1 | 0 | 8.181707 | 1.466179 | -0.127756 |

**15A**: E = -1825.3175971 a.u., ω1=1.1 $cm^{-1}$

| Center Number | Atomic Number | Atomic Type | Coordinates (Angstroms) X | Y | Z |
|---|---|---|---|---|---|
| 1 | 6 | 0 | 7.651961 | -0.422330 | 0.198996 |
| 2 | 6 | 0 | 7.476098 | 0.817809 | 0.843475 |
| 3 | 6 | 0 | 6.213468 | 1.341474 | 0.990877 |
| 4 | 6 | 0 | 5.077090 | 0.644147 | 0.500895 |
| 5 | 6 | 0 | 5.279534 | -0.611215 | -0.147362 |
| 6 | 6 | 0 | 6.569663 | -1.133211 | -0.293573 |
| 7 | 16 | 0 | 3.945511 | -1.536453 | -0.776140 |
| 8 | 6 | 0 | 2.563679 | -0.560724 | -0.396594 |
| 9 | 6 | 0 | 2.730974 | 0.710921 | 0.287977 |
| 10 | 7 | 0 | 3.871489 | 1.232632 | 0.683821 |
| 11 | 6 | 0 | 1.311429 | -1.014232 | -0.767695 |
| 12 | 6 | 0 | 0.167296 | -0.239503 | -0.489758 |
| 13 | 6 | 0 | 0.313626 | 1.027653 | 0.179085 |
| 14 | 6 | 0 | 1.536032 | 1.474404 | 0.549971 |
| 15 | 7 | 0 | -1.074509 | -0.633892 | -0.839275 |
| 16 | 6 | 0 | -1.436695 | -1.873394 | -1.462769 |
| 17 | 6 | 0 | -1.958353 | -1.851228 | -2.754286 |
| 18 | 6 | 0 | -2.335151 | -3.057073 | -3.336574 |
| 19 | 6 | 0 | -2.193157 | -4.271273 | -2.672028 |
| 20 | 6 | 0 | -1.671909 | -4.273175 | -1.379314 |
| 21 | 6 | 0 | -1.300770 | -3.078179 | -0.768438 |
| 22 | 1 | 0 | 8.648838 | -0.831203 | 0.084010 |
| 23 | 1 | 0 | 8.337518 | 1.355229 | 1.219651 |
| 24 | 1 | 0 | 6.038706 | 2.292273 | 1.478598 |
| 25 | 1 | 0 | 6.720987 | -2.085962 | -0.787717 |
| 26 | 1 | 0 | 1.204023 | -1.955577 | -1.290903 |
| 27 | 1 | 0 | -0.573088 | 1.616848 | 0.385508 |
| 28 | 1 | 0 | 1.666529 | 2.422317 | 1.056769 |
| 29 | 1 | 0 | -1.823987 | 0.030108 | -0.690692 |
| 30 | 16 | 0 | -2.993134 | -3.025807 | -5.012490 |
| 31 | 1 | 0 | -1.564073 | -5.208002 | -0.843036 |
| 32 | 1 | 0 | -0.919672 | -3.076486 | 0.246055 |
| 33 | 1 | 0 | -2.473943 | -5.195448 | -3.161625 |

| | | | | | |
|---|---|---|---|---|---|
| 34 | 1 | 0 | -2.050868 | -0.924630 | -3.307678 |
| 35 | 8 | 0 | -2.544551 | -1.796540 | -5.629486 |
| 36 | 8 | 0 | -2.796490 | -4.332148 | -5.618345 |
| 37 | 8 | 0 | -4.595910 | -2.854164 | -4.711397 |
| 38 | 1 | 0 | -5.065477 | -3.638716 | -5.036563 |

**15B**: E = -1825.3168073 a.u., ω1=9.8 $cm^{-1}$

| Center Number | Atomic Number | Atomic Type | Coordinates (Angstroms) X | Y | Z |
|---|---|---|---|---|---|
| 1 | 6 | 0 | -7.667316 | -2.629379 | 0.047766 |
| 2 | 6 | 0 | -7.829271 | -1.353240 | -0.525874 |
| 3 | 6 | 0 | -6.736830 | -0.537471 | -0.702793 |
| 4 | 6 | 0 | -5.439875 | -0.966455 | -0.313213 |
| 5 | 6 | 0 | -5.300044 | -2.263679 | 0.265675 |
| 6 | 6 | 0 | -6.419254 | -3.084723 | 0.441381 |
| 7 | 16 | 0 | -3.749584 | -2.874441 | 0.771683 |
| 8 | 6 | 0 | -2.662736 | -1.578900 | 0.382564 |
| 9 | 6 | 0 | -3.166360 | -0.353981 | -0.217851 |
| 10 | 7 | 0 | -4.420373 | -0.100476 | -0.523010 |
| 11 | 6 | 0 | -1.318605 | -1.728942 | 0.655745 |
| 12 | 6 | 0 | -0.402716 | -0.695437 | 0.357930 |
| 13 | 6 | 0 | -0.880458 | 0.518570 | -0.247409 |
| 14 | 6 | 0 | -2.199791 | 0.671286 | -0.518710 |
| 15 | 7 | 0 | 0.908236 | -0.877594 | 0.624406 |
| 16 | 6 | 0 | 1.966859 | 0.075541 | 0.481152 |
| 17 | 6 | 0 | 3.046141 | -0.243842 | -0.340925 |
| 18 | 6 | 0 | 4.088995 | 0.669256 | -0.449552 |
| 19 | 6 | 0 | 4.074895 | 1.887201 | 0.223415 |
| 20 | 6 | 0 | 2.989982 | 2.187266 | 1.044968 |
| 21 | 6 | 0 | 1.941456 | 1.282162 | 1.185865 |
| 22 | 1 | 0 | -8.530460 | -3.269890 | 0.185280 |
| 23 | 1 | 0 | -8.814689 | -1.019379 | -0.825578 |
| 24 | 1 | 0 | -6.821141 | 0.449551 | -1.139965 |
| 25 | 1 | 0 | -6.310941 | -4.069490 | 0.881065 |
| 26 | 1 | 0 | -0.953251 | -2.644874 | 1.108158 |
| 27 | 1 | 0 | -0.174304 | 1.295299 | -0.506188 |
| 28 | 1 | 0 | -2.579761 | 1.569866 | -0.988727 |
| 29 | 1 | 0 | 1.186783 | -1.801857 | 0.929730 |
| 30 | 16 | 0 | 5.472404 | 0.259825 | -1.527264 |
| 31 | 1 | 0 | 2.966627 | 3.125139 | 1.586159 |
| 32 | 1 | 0 | 1.114753 | 1.503791 | 1.850387 |
| 33 | 1 | 0 | 4.890242 | 2.588451 | 0.097199 |
| 34 | 1 | 0 | 3.069315 | -1.167068 | -0.907175 |

| | | | | | |
|---|---|---|---|---|---|
| 35 | 8 | 0 | 5.009934 | -0.741420 | -2.463577 |
| 36 | 8 | 0 | 6.124410 | 1.490433 | -1.942205 |
| 37 | 8 | 0 | 6.471402 | -0.498086 | -0.469496 |
| 38 | 1 | 0 | 7.292328 | 0.012987 | -0.388018 |

**16A**: E = -1201.4362853 a.u., ω1=26.7 cm$^{-1}$

| Center Number | Atomic Number | Atomic Type | Coordinates (Angstroms) X | Y | Z |
|---|---|---|---|---|---|
| 1 | 6 | 0 | 5.404214 | -1.621204 | -0.141055 |
| 2 | 6 | 0 | 5.822960 | -0.283365 | -0.011655 |
| 3 | 6 | 0 | 4.888824 | 0.722950 | 0.071236 |
| 4 | 6 | 0 | 3.500559 | 0.429841 | 0.029641 |
| 5 | 6 | 0 | 3.099617 | -0.932138 | -0.100408 |
| 6 | 6 | 0 | 4.058346 | -1.947755 | -0.186172 |
| 7 | 16 | 0 | 1.418168 | -1.386496 | -0.159946 |
| 8 | 6 | 0 | 0.586486 | 0.131529 | -0.029097 |
| 9 | 6 | 0 | 1.340280 | 1.370876 | 0.085042 |
| 10 | 7 | 0 | 2.649059 | 1.482396 | 0.110437 |
| 11 | 6 | 0 | -0.794153 | 0.131791 | -0.046033 |
| 12 | 6 | 0 | -1.505311 | 1.347832 | 0.042657 |
| 13 | 6 | 0 | -0.775328 | 2.586855 | 0.141159 |
| 14 | 6 | 0 | 0.577140 | 2.592697 | 0.164805 |
| 15 | 7 | 0 | -2.850682 | 1.408916 | 0.024428 |
| 16 | 6 | 0 | -3.783923 | 0.324444 | 0.025040 |
| 17 | 6 | 0 | -4.787676 | 0.303447 | -0.945711 |
| 18 | 6 | 0 | -5.732365 | -0.718812 | -0.932564 |
| 19 | 6 | 0 | -5.674168 | -1.715393 | 0.040817 |
| 20 | 6 | 0 | -4.674734 | -1.682921 | 1.012628 |
| 21 | 6 | 0 | -3.730320 | -0.660124 | 1.015091 |
| 22 | 1 | 0 | 6.142374 | -2.411702 | -0.207061 |
| 23 | 1 | 0 | 6.880027 | -0.051057 | 0.021028 |
| 24 | 1 | 0 | 5.172606 | 1.763277 | 0.169519 |
| 25 | 1 | 0 | 3.750394 | -2.982146 | -0.286790 |
| 26 | 1 | 0 | -1.339233 | -0.796984 | -0.149947 |
| 27 | 1 | 0 | -1.329835 | 3.516752 | 0.205498 |
| 28 | 1 | 0 | 1.141807 | 3.513080 | 0.245166 |
| 29 | 1 | 0 | -3.255454 | 2.335301 | -0.028110 |
| 30 | 1 | 0 | -6.510551 | -0.737387 | -1.685921 |
| 31 | 1 | 0 | -4.640894 | -2.443389 | 1.783572 |
| 32 | 1 | 0 | -2.980270 | -0.609046 | 1.795371 |
| 33 | 1 | 0 | -6.411274 | -2.509177 | 0.048102 |
| 34 | 1 | 0 | -4.821726 | 1.073928 | -1.707874 |

**16B**: E = -1201.4354139 a.u., ω1=25.8 $cm^{-1}$

| Center Number | Atomic Number | Atomic Type | Coordinates (Angstroms) X | Y | Z |
|---|---|---|---|---|---|
| 1 | 6 | 0 | -7.617478 | -2.676487 | 0.165200 |
| 2 | 6 | 0 | -7.835807 | -1.335255 | -0.202009 |
| 3 | 6 | 0 | -6.765850 | -0.486256 | -0.365611 |
| 4 | 6 | 0 | -5.437340 | -0.945965 | -0.168758 |
| 5 | 6 | 0 | -5.240218 | -2.308458 | 0.202173 |
| 6 | 6 | 0 | -6.335588 | -3.163105 | 0.366527 |
| 7 | 16 | 0 | -3.645684 | -2.962998 | 0.464022 |
| 8 | 6 | 0 | -2.603373 | -1.606302 | 0.165167 |
| 9 | 6 | 0 | -3.166509 | -0.315790 | -0.203283 |
| 10 | 7 | 0 | -4.442991 | -0.041323 | -0.349358 |
| 11 | 6 | 0 | -1.240515 | -1.770015 | 0.287905 |
| 12 | 6 | 0 | -0.357462 | -0.687294 | 0.063648 |
| 13 | 6 | 0 | -0.895346 | 0.591917 | -0.318861 |
| 14 | 6 | 0 | -2.234126 | 0.757377 | -0.444821 |
| 15 | 7 | 0 | 0.969455 | -0.883813 | 0.186040 |
| 16 | 6 | 0 | 2.015651 | 0.091637 | 0.131807 |
| 17 | 6 | 0 | 3.100225 | -0.137032 | -0.717669 |
| 18 | 6 | 0 | 4.147474 | 0.779000 | -0.752504 |
| 19 | 6 | 0 | 4.111173 | 1.917152 | 0.052347 |
| 20 | 6 | 0 | 3.029780 | 2.133949 | 0.905209 |
| 21 | 6 | 0 | 1.982021 | 1.218677 | 0.956915 |
| 22 | 1 | 0 | -8.461919 | -3.343290 | 0.293372 |
| 23 | 1 | 0 | -8.845924 | -0.976202 | -0.354119 |
| 24 | 1 | 0 | -6.893946 | 0.551586 | -0.646625 |
| 25 | 1 | 0 | -6.182876 | -4.198424 | 0.648792 |
| 26 | 1 | 0 | -0.830797 | -2.734181 | 0.570547 |
| 27 | 1 | 0 | -0.218307 | 1.408353 | -0.527726 |
| 28 | 1 | 0 | -2.659911 | 1.705973 | -0.747561 |
| 29 | 1 | 0 | 1.272301 | -1.839626 | 0.327815 |
| 30 | 1 | 0 | 4.988186 | 0.605488 | -1.413223 |
| 31 | 1 | 0 | 3.008999 | 3.006607 | 1.546865 |
| 32 | 1 | 0 | 1.162084 | 1.362623 | 1.650492 |
| 33 | 1 | 0 | 4.927723 | 2.628332 | 0.021814 |
| 34 | 1 | 0 | 3.116895 | -1.015854 | -1.352529 |
